# Effect of a lifestyle intervention in obese infertile women on cardiometabolic health and quality of life: A randomized controlled trial

**DOI:** 10.1371/journal.pone.0190662

**Published:** 2018-01-11

**Authors:** Lotte van Dammen, Vincent Wekker, Anne M. van Oers, Meike A. Q. Mutsaerts, Rebecca C. Painter, Aeilko H. Zwinderman, Henk Groen, Cornelieke van de Beek, Anneke C. Muller Kobold, Walter K. H. Kuchenbecker, Ron van Golde, Gerrit J. E. Oosterhuis, Niels E. A. Vogel, Ben Willem J. Mol, Tessa J. Roseboom, Annemieke Hoek, on behalf of the LIFEstyle study group

**Affiliations:** 1 Department of Epidemiology, University of Groningen, University Medical Centre Groningen, Groningen, the Netherlands; 2 Department of Obstetrics and Gynecology, University of Groningen, University Medical Centre Groningen, Groningen, the Netherlands; 3 Department of Obstetrics and Gynecology, Academic Medical Centre, University of Amsterdam, Amsterdam, the Netherlands; 4 Department of Clinical Epidemiology, Biostatistics and Bioinformatics, Amsterdam Public Health research institute, Academic Medical Centre, University of Amsterdam, Amsterdam, the Netherlands; 5 Department of General Practice, University Medical Centre Utrecht, University of Utrecht, Utrecht, the Netherlands; 6 Department of Laboratory Medicine, University of Groningen, University Medical Centre Groningen, Groningen, the Netherlands; 7 Department of Obstetrics and Gynecology, Isala Clinics, Zwolle, the Netherlands; 8 Department of Obstetrics and Gynecology, Maastricht University Medical Centre, Maastricht University, Maastricht, the Netherlands; 9 Department of Obstetrics and Gynecology, St. Antonius Hospital, Nieuwegein, the Netherlands; 10 Department of Obstetrics and Gynecology, Martini Hospital, Groningen, the Netherlands; 11 School of Medicine, Robinson Research Institute, University of Adelaide, Adelaide, Australia; Victoria University, AUSTRALIA

## Abstract

**Background:**

The prevalence of obesity, an important cardiometabolic risk factor, is rising in women. Lifestyle improvements are the first step in treatment of obesity, but the success depends on factors like timing and motivation. Women are especially receptive to advice about lifestyle before and during pregnancy. Therefore, we hypothesize that the pre-pregnancy period provides the perfect window of opportunity to improve cardiometabolic health and quality of life of obese infertile women, by means of a lifestyle intervention.

**Methods and findings:**

Between 2009–2012, 577 infertile women between 18 and 39 years of age, with a Body Mass Index of ≥ 29 kg/m2, were randomized to a six month lifestyle intervention preceding infertility treatment, or to direct infertility treatment. The goal of the intervention was 5–10% weight loss or a BMI < 29 kg/m^2^. Cardiometabolic outcomes included weight, waist- and hip circumference, body mass index, systolic and diastolic blood pressure, fasting glucose and insulin, HOMA-IR, hs-CRP, lipids and metabolic syndrome. All outcomes were measured by research nurses at randomization, 3 and 6 months. Self-reported quality of life was also measured at 12 months. Three participants withdrew their informed consent, and 63 participants discontinued the intervention program. Intention to treat analysis was conducted. Mixed effects regression models analyses were performed. Results are displayed as estimated mean differences between intervention and control group. Weight (-3.1 kg 95% CI: -4.0 to -2.2 kg; P < .001), waist circumference (-2.4 cm 95% CI: -3.6 to -1.1 cm; P < .001), hip circumference (-3.0 95% CI: -4.2 to -1.9 cm; P < .001), BMI (-1.2 kg/m^2^ 95% CI: -1.5 to -0.8 kg/m^2^; P < .001), systolic blood pressure (-2.8 mmHg 95% CI: -5.0 to -0.7 mmHg; P = .01) and HOMA-IR (-0.5 95% CI: -0.8 to -0.1; P = .01) were lower in the intervention group compared to controls. Hs-CRP and lipids did not differ between groups. The odds ratio for metabolic syndrome in the intervention group was 0.53 (95% CI: 0.33 to 0.85; P < .01) compared to controls. Physical QoL scores were higher in the lifestyle intervention group (2.2 95% CI: 0.9 to 3.5; P = .001) while mental QoL scores did not differ.

**Conclusions:**

In obese infertile women, a lifestyle intervention prior to infertility treatment improves cardiometabolic health and self-reported physical quality of life (LIFEstyle study: Netherlands Trial Register: NTR1530).

## Introduction

Cardiometabolic disease is the leading cause of death in women worldwide. In the United States, cardiometabolic diseases are responsible for more than 25% of mortality in women [1]. In the last forty years, the global age standardized prevalence of obesity in women, as one of the major modifiable risk factors for cardiometabolic diseases, has more than doubled; from 6.4% (5.1 to 7.8) in 1975 to 14.9% (13.6 to 16.1) in 2014 [2]. The prevalence of obesity among women of childbearing age is even higher: 16.4% in 2009. Obesity reduces fertility [3, 4] and increases the risk of atherosclerosis, hypertension, dyslipidemia, chronic inflammation, and insulin resistance [5, 6]. The clustering of these cardiometabolic risk factors is known as metabolic syndrome (MetS) [7, 8]. In women, MetS doubles the risk of cardiovascular mortality [9, 10]. Both cardiometabolic and reproductive morbidity reduce physical and mental quality of life (QoL) [11, 12]. QoL is an important outcome, as it reflects individual perception of both mental and physical wellbeing. Moreover, poor QoL has been linked to an increased risk of all-cause mortality [13].

Current international guidelines state that lifestyle improvements are the cornerstone of primary prevention and treatment of obesity and cardiometabolic diseases [14, 15]. Lifestyle improvements are difficult to achieve and the effects on weight reduction and retention are disappointing [16]. Despite that lifestyle interventions in patients at risk of type 2 diabetes led to positive effects on cardiometabolic outcomes like Body Mass Index (BMI), waist and hip circumference, fasting plasma glucose levels and blood pressure [17]. The success of a lifestyle intervention depends on several factors including intrinsic motivation, but also timing, duration, and intensity of the intervention are important [18, 19]. In general, women are especially receptive to advice about lifestyle before and during pregnancy. For example, 39% of women who are planning a pregnancy discontinue tobacco smoking, which is almost eight times higher than quitting rates in women not planning a pregnancy [20, 21]. Therefore we hypothesized that obese women might benefit from a lifestyle intervention prior to infertility treatment.

The LIFEstyle study is the first large randomized controlled trial (RCT) to investigate the effects of a six month lifestyle intervention among obese infertile women who intended to become pregnant. The effects of the intervention on reproductive outcomes were published in May 2016 [22]. Women in the intervention group had significantly more natural conceptions and a comparable rate of ongoing pregnancies, although there was no increased rate of vaginal birth of a healthy singleton at term. Here we report the effects on cardiometabolic health and QoL.

## Materials and methods

### Design

The LIFEstyle study, a multi-center RCT was conducted in 17 general and six academic medical centers across the Netherlands. Participants were included in the study between June 9, 2009, and June 22, 2012. The study was conducted following the principles of the Declaration of Helsinki and approved by the medical ethics committee of the University Medical Centre Groningen (UMCG) (METc code: 2008/284), as well as by the board of directors of the other participating hospitals (N = 22). All included participants gave written informed consent. The trial was registered in the Netherlands Trial Registry (NTR 1530) and the LIFEstyle study protocol has been published [22, 23].

### Participants

Infertile women aged between 18 and 39 years, with a BMI of ≥ 29 kg/m^2^ were eligible. Infertility was defined as chronic anovulation or unsuccessful conception for at least 12 months [24]. Women with severe endometriosis, premature ovarian insufficiency, endocrinopathy, untreated preexisting hypertension, or women with a history of hypertension related pregnancy complications were excluded from participating [23].

### Randomization

Participants were randomized 1:1 between a six month lifestyle intervention preceding infertility treatment or direct infertility treatment. Stratified randomization according to trial center and ovulatory status was performed at the Academic Medical Centre in Amsterdam with an online program.

### Lifestyle intervention

The goal of the lifestyle intervention was a 5–10% weight loss or a BMI < 29 kg/m^2^ within six months. The dietary therapy, using an online diary, aimed at caloric reduction of 600 kcal, with a minimum intake of 1200 kcal/day [25]. Participants were advised to be physically active on moderate-intensity level for at least two or three times a week. Daily physical activity was stimulated with the use of a pedometer, aimed at 10.000 steps per day. The individualized behavioral modification was focused on creating awareness of lifestyle predisposing to obesity. The lifestyle intervention was in concordance with the recommendations of the National Institute of Health [26].

The lifestyle intervention consisted of face-to-face sessions of approximately 30 minutes at the outpatient clinics, four in the first three months and two in the last three months and four sessions by telephone or e-mail. The intervention coaches had a background in nursing or nutritional science and were trained to practice motivational counselling techniques [27]. A structured software program was used to minimize the practice variation between the intervention coaches [28].

Participants discontinued the intervention if they became pregnant, but in case of a miscarriage they could re-enter the intervention. Participants who successfully reached the goal of the lifestyle intervention could proceed with their indicated infertility treatment before they had finished the six month intervention [29]. If women missed two or more consecutive sessions, they were considered to have not completed the intervention.

### Control strategy

Participants allocated to the control group were treated directly in accordance with Dutch infertility guidelines, irrespective of their BMI [29]. Anovulatory women started with ovulation induction with clomiphene citrate. If pregnancy did not occur in six to 12 cycles or if women developed clomiphene resistance, gonadotropin therapy was initiated in a low-dose step-up regimen for a maximum of 12 cycles [30]. In ovulatory women, treatment depended on the estimated probability of natural conception according to the Hunault prediction model [31]. If the probability was estimated to be < 30%, women were offered up to six cycles of intrauterine insemination (IUI). If the probability was estimated to be > 30%, expectant management was proposed for six to12 months. In vitro fertilization was initiated in women with tubal disease or after IUI had failed. Intracytoplasmic sperm injection was used in couples with severe male-factor infertility.

### Patient involvement

A single center pilot study had been performed previously to evaluate the intervention. During this pilot study, it became clear that patients preferred individual sessions with evaluation of personal goals instead of group sessions, because individual care was considered less time consuming. Otherwise, patients were not involved in the design nor in the conduct of the LIFEstyle study. The Dutch patient support group ‘Freya’ invited their peers on their website to participate in the trial. Participants received a personal letter with the results of the trial in layman’s terms.

### Outcome measures

The prespecified outcomes were weight, BMI (weight in kg divided by height in m^2^), waist- and hip circumference and ratio, systolic and diastolic blood pressure, fasting serum concentrations of glucose and insulin, and physical and mental QoL. Insulin resistance was quantified using the homeostasis model assessment of insulin resistance (HOMA-IR). This model was defined as fasting insulin concentration in μU/mL multiplied by fasting glucose concentration in mmol/L divided by 22.5 [32].

Non-prespecified outcomes were fasting serum concentrations of triglycerides, total cholesterol, low density lipoprotein cholesterol (LDL-C), high density lipoprotein cholesterol (HDL-C), and high sensitive C-reactive protein (hs-CRP). Participants were identified with MetS if they met at least three of the following criteria: (1) glucose ≥5.6 mmol/L; (2) HDL-C <1.3 mmol/L; (3) triglycerides ≥1.7 mmol/L; (4) waist circumference ≥88 cm or (5) blood pressure ≥130/85 mmHg, based on the 2001 revised criteria of the National Cholesterol Education Program ATP III [8].

The power calculation was based on the primary outcome of the LIFEstyle study: the vaginal birth of a healthy singleton at 37 weeks or more within 24 months. This outcome and the power calculations have been published previously [22].

### Study procedures

In all non-pregnant participants, during the hospital visits (i.e. at randomization, three and six months), research nurses that were not involved in the lifestyle intervention coaching, measured body weight (kg), height (cm), waist circumference (measured in cm at the narrowest part between the lower rib and iliac crest), hip circumference (measured in cm at the level of the greatest gluteal protuberance), and blood pressure (mmHg, measured manually or electronically in sitting position). Fasting blood samples were collected by venipuncture into one serum and one sodium fluoride vacutainer tube. Serum samples were kept at room temperature for a minimum of 30 minutes for coagulation, and then centrifuged at 1700 x g for 10 minutes at 4°C to obtain serum and plasma, which were stored at -80°C. The biochemical analyses were performed in the central laboratory of the University Medical Centre Groningen after the trial had been completed. Hence, results were unknown to participants and care providers until after trial completion and could not have impacted the participants’ management. Hs-CRP was measured with an immuno-turbidimetric assay. Triglycerides, total cholesterol, HDL-C, and LDL-C concentrations were measured using enzymatic colorimetric assays. Fasting plasma glucose was measured with an enzymatic UV test (hexokinase method). All previously described assays were produced by Roche^®^ Modular P (Roche^®^, Manheim, Germany). Insulin was measured with the Architect manufactured by Abbott Diagnostics (Lake Forest, Illinois, United States), using an chemiluminescent micro particle immunoassay. The intra- and interassay variation was respectively 0.5–4.0% and 1.9–6.2% for hs-CRP; 1.5% and 1.8% for triglycerides; 0.8% and 1.7% for total cholesterol; 0.6–0.95% and 1.2–1.3% for HDL-C; 0.72%-0.81% and 1.03–1.18% for LDL-C; 1.0% and 1.7% for glucose; 2.1–4.1% and 2.4–4.2% for insulin.

Participants filled in the 36-Item Short Form Health Survey (SF-36) at the time of randomization, and three, six, and 12 months later, using a web-based survey. The SF-36 is a general health related QoL measure, consisting of 36 items [33]. This questionnaire consists of a Physical Component Score (PCS) and a Mental Component Score (MCS), in which higher scores represents a better QoL. The SF-36 is sensitive to changes during lifestyle interventions [34]. The Dutch SF-36 is widely used and has demonstrated good reliability (Cronbach’s α = 0.71–0.92) [35]. Physical activity was measured with the validated Short Questionnaire to Assess Health-enhancing physical activity (SQUASH) [36]. The durations of leisure time and total moderate to vigorous physical activity time per week were calculated. A Food Frequency Questionnaire was used to assess the intake of fruit, vegetables, sugary drinks, sweet snacks and savory snacks.

### Statistical analysis

**Fig 1:**
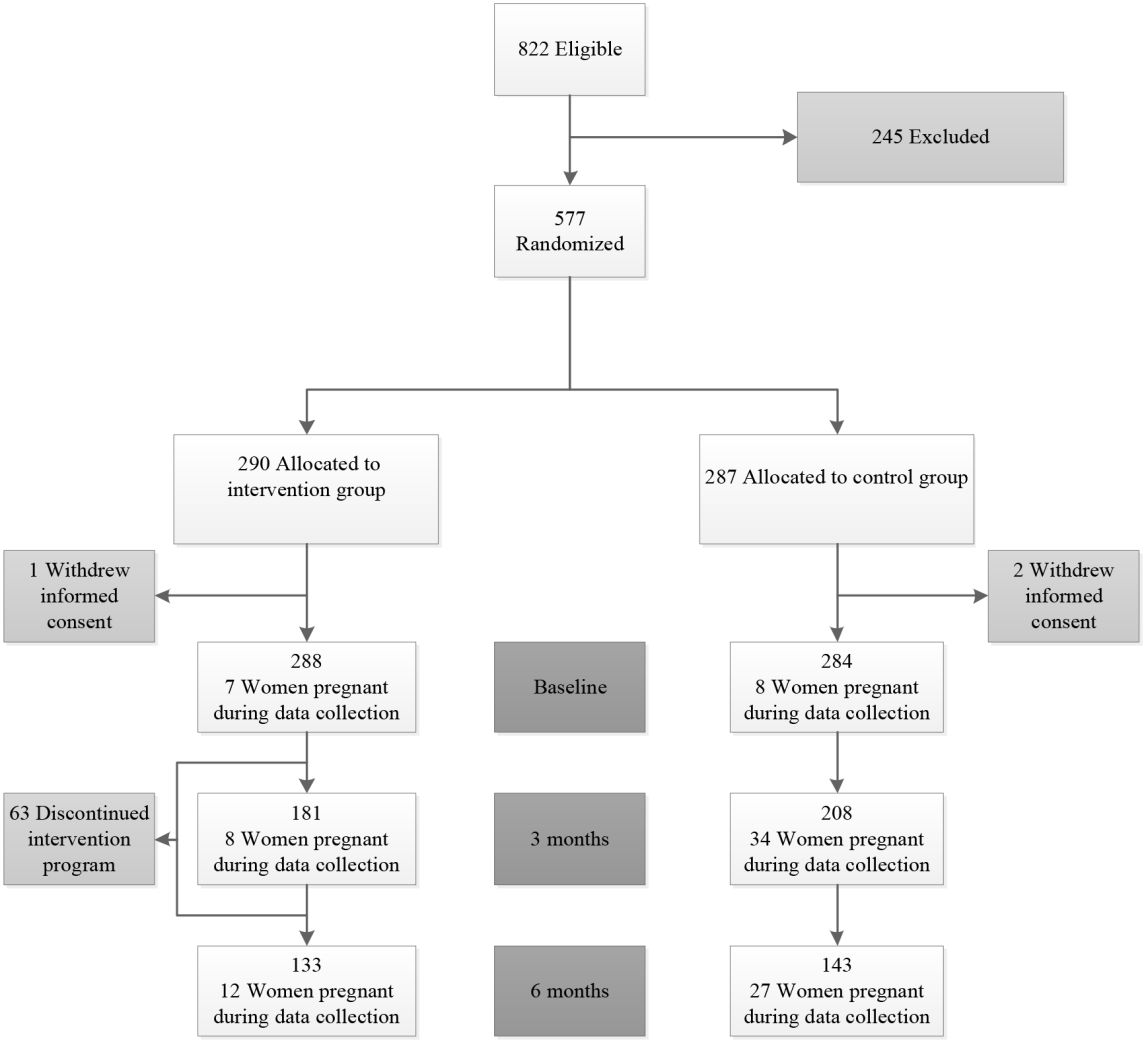
Flowchart of participants. Baseline values are based on number of participants for whom BMI was available. Due to intercurrent pregnancies, or reaching the goal of the intervention prior to 24 weeks (BMI<29 kg/m2 or >5% weight loss) or because participants did not attend the hospital visit, the numbers of participants are decreasing over time.

The analyses were performed on an intention-to-treat basis: participants were analyzed in the group they were randomly assigned to, regardless of whether they completed the intervention. Mixed effects regression model analyses were constructed with a random intercept per patient for all outcomes, and with a variance component structure. Fixed effects were follow-up time, randomization group, and their interaction. The baseline measurement was included as a covariate. The dependent variable was the cardiometabolic outcome measure or QoL score. In order to analyze the odds of metabolic syndrome, a generalized linear mixed effects regression model was used. All participants with at least one measurement were included in the analyses (Fig 1). Data collected in pregnant participants, unknown to be pregnant at time of measurement or bloodsampling, were excluded since pregnancy is known to have substantial effects on cardiometabolic outcomes. Awareness of being pregnant may influence QoL, hence QoL data collected more than two weeks after the conception date was excluded. Since infertility treatment may also influence cardiometabolic health, an additional analysis was performed adjusted for receiving any type of infertility treatment at the time of the visit. Outcomes of the mixed effects regression model analyses are presented as estimated marginal means and 95% confidence intervals (CI). For cardiometabolic outcomes that were significantly affected by the intervention at 3 months, mediation analyses were performed using the difference in scores for physical activity and dietary outcomes between randomization and three months. All statistical analyses were performed using IBM SPSS version 24.0 (Armonk, NY, USA). The mediation analyses were performed using model 4, with 5000 bootstrapped samples for the estimation of 95% confidence intervals, of the PROCESS macro (V.2.16.3) for SPSS [37].

## Results

822 women were eligible to participate, of whom 245 refused participation. 577 women were randomized (Fig 1). Three women withdrew informed consent after randomization, leaving a total of 289 in the intervention group and 285 women in the control group. A total of 63 of the 289 women discontinued the lifestyle intervention, due to lack of motivation (N = 40), relationship problems (N = 12), or other reasons (N = 11). Table 1 shows that the baseline characteristics of included women were similar between the two groups.

**Table 1:** Baseline characteristics of study participants in intervention and control group.

	Intervention group (N = 289)	Control group (N = 285)
**Age, years, mean (SD)**	29.7 (4.5)	29.8 (4.6)
**Caucasian, n (%)**	256 (88.6)	246 (86.3)
**Education, n (%)**		
- **Primary school (4–12 years)**	17 (5.9)	10 (3.5)
- **Secondary Education**	68 (23.5)	63 (22.1)
- **Intermediate Vocational Education**	135 (46.7)	131 (46.0)
- **Advanced Vocational Education or University**	56 (19.4)	69 (24.2)
- **Unknown**	13 (4.5)	12 (4.2)
**Current smoker, n (%)**	76 (26.7)	60 (21.1)
**Primary infertility, n(%)**	183 (63.3)	186 (65.5)
**Infertility assessment**		
- **Anovulatory infertility, n (%)**	128 (44.3)	141(49.5)
◯ **PCOS diagnosed by Rotterdam 2003 criteria, n/total anovulatory (%)**(Rotterdam 2003 criteria [38])	97/128 (75.8)	104/141 (73.8)

**Abbreviations:** BMI, Body Mass Index; PCOS, Polycystic Ovary Syndrome; Hs-CRP, High sensitive C-Reactive Protein; HDL-C, High Density Lipoprotein cholesterol; LDL-C, Low Density Lipoprotein cholesterol; HOMA-IR, Homeostasis Model of Insulin Resistance; N, number; SD, Standard deviation.

Cardiometabolic data were collected at time of randomization, as well as three months (median was 14 (interquartile range (IQR) 13–15) weeks), and six months (median was 27 (IQR 25–30) weeks) after randomization. The effects of the lifestyle intervention on cardiometabolic health, at three and six months, are shown in Table 2.

**Table 2:** Cardiometabolic outcomes at baseline (mean (standard error (SE))), three and six months for the intervention and control group, unless stated otherwise.

	Baseline		3 months^α^			6 months^α^		
	Intervention group (N = 289)	Control group (N = 285)	Intervention group (N = 289)	Control group (N = 285)	P value	Intervention group (N = 289)	Control group (N = 285)	P value
**Anthropometrics**								
- **Weight (kg)**	103.6 (0.8)	103.0 (0.7)	99.9 (0.3)	102.6 (0.3)	< 0.0001	98.6 (0.4)	102.2 (0.4)	< 0.001
- **BMI (kg/m**^**2**^**)**	36.1 (0.2)	36.0 (0.2)	34.8 (0.1)	35.8 (0.1)	< 0.0001	34.3 (0.1)	35.6 (0.1)	< 0.001
- **Waist circumference (cm)**	108.2 (0.6)	107.9 (0.6)	104.6 (0.5)	106.3 (0.5)	0.01	102.9 (0.5)	106.3 (0.6)	< 0.001
- **Hip circumference (cm)**	125.0 (0.5)	125.2 (0.5)	121.8 (0.4)	124.2 (0.4)	< 0.0001	120.4 (0.5)	124.6 (0.5)	< 0.001
- **Waist-hip circumference ratio**	0.87 (0.004)	0.86 (0.004)	0.86 (0.01)	0.86 (0.01)	0.975	0.86 (0.01)	0.86 (0.01)	0.960
**Blood pressure**								
- **Systolic blood pressure (mmHg)**	126.1 (0.9)	127.0 (0.8)	124.2 (0.9)	126.3 (0.9)	0.096	121.3 (1.0)	125.4 (1.0)	0.005
- **Diastolic blood pressure (mmHg)**	79.7 (0.6)	80.1 (0.5)	78.7 (0.6)	79.8 (0.6)	0.219	78.4 (0.7)	81.2 (0.7)	0.009
**Biochemical measures**								
- **Hs-CRP (mg/l)**	5.6 (0.3)	5.6 (0.3)	5.17 (0.38)	5.84 (0.40)	0.223	5.15 (0.45)	6.19 (0.47)	0.303
- **Triglycerides (mmol/l)**	1.2 (0.05)	1.4 (0.1)	1.34 (0.05)	1.29 (0.05)	0.470	1.25 (0.06)	1.45 (0.06)	0.012
- **Total cholesterol (mmol/l)**	4.8 (0.06)	4.8 (0.06)	4.79 (0.05)	4.79 (0.05)	0.354	4.75 (0.05)	4.90 (0.05)	0.046
- **HDL-C (mmol/l)**	1.2 (0.02)	1.2 (0.02)	1.16 (0.01)	1.17 (0.02)	0.624	1.19 (0.02)	1.21 (0.02)	0.346
- **LDL-C (mmol/l)**	3.1 (0.05)	3.1 (0.05)	3.02 (0.04)	3.12 (0.04)	0.062	3.08 (0.04)	3.14 (0.05)	0.329
- **Glucose (mmol/l)**	5.3 (0.04)	5.4 (0.05)	5.32 (0.05)	5.41 (0.05)	0.160	5.24 (0.05)	5.40 (0.06)	0.040
- **Insulin (pmol/L)**	96.5 (3.3)	103.5 (4.1)	89.9 (3.4)	104.6 (3.6)	0.003	89.4 (3.9)	95.4 (4.1)	0.297
- **HOMA-IR**	3.3 (0.1)	3.6 (0.2)	3.12 (0.13)	3.72 (0.14)	0.002	3.05 (0.15)	3.27 (0.16)	0.303
**Metabolic syndrome, n (%)**	121 (52.4)	133 (58.3)	102 (42.6)	137 (57.1)	0.042	79 (33.0)	120 (49.9)	0.036

^**α**^ Values are estimated means (SE) of mixed effects regression model analyses, unless stated otherwise.

**Abbreviations:** BMI, Body Mass Index; Hs-CRP, High sensitive C-Reactive Protein; HDL-C, High Density Lipoprotein cholesterol; LDL-C, Low Density Lipoprotein cholesterol; HOMA-IR, Homeostasis Model of Insulin Resistance; N, number.

The effects of the intervention on the outcomes did not significantly change over time, as demonstrated by the fact that interaction between time and randomization group did not achieve statistical significance for any of the outcomes (data not shown). The mean weight in the intervention group over the two follow-up measurements was lower, compared to the control group (-3.1 kg 95% CI: -4.0 to -2.2 kg; P < 0.001). BMI was lower in the intervention group compared to the control group, (-1.2 kg/m^2^ 95% CI: -1.5 to -0.8 kg/m^2^; P < 0.001). Waist- and hip circumference were lower in the intervention group compared to the control group respectively (-2.4 cm 95% CI: -3.6 to -1.1 cm; P < 0.001), and (-3.0 95% CI: -4.2 to -1.9 cm; P < 0.001). Waist-hip circumference ratio did not differ between groups. Systolic blood pressure was lower in the intervention group compared to the control group (-2.8 mmHg 95% CI: -5.0 to -0.7 mmHg; P = 0.01). Diastolic blood pressure was lower in the intervention group compared to the control group (-1.7 mmHg 95% CI: -3.3 to -0.1 mmHg; P = 0.04). In the intervention group hs-CRP was lower compared to the control group, (-0.8 mg/l 95% CI: -1.7 to 0.1 mg/L), but this difference was not statistically significant (P = 0.07). Triglycerides did not differ between the control and intervention group. Total cholesterol was lower in the intervention group compared to the control group (-3.9 mg/dL 95% CI: -7.7 to 0.4 mg/dL), but this difference was not statistically significant (P = 0.09). There was no difference between the groups in HDL-C. LDL-C was, compared to the control group, lower in the intervention group (-3.9 mg/dL 95% CI: -7.7 to 0.4 mg/dL), although not statistically significant (P = 0.07). In the intervention group glucose was lower compared to the control group (-1.8 mg/dL 95% CI: -3.6 to -0.2 mg/dL; P = 0.04). Insulin was lower in the intervention group compared to the control group (-11.1 pmol/L 95% CI: -20.1 to -2.8 pmol/L; P = .01). In the intervention group HOMA-IR was lower, compared to the control group (-0.5 95% CI: -0.8 to -0.1; P = 0.01).

**Fig 2:**
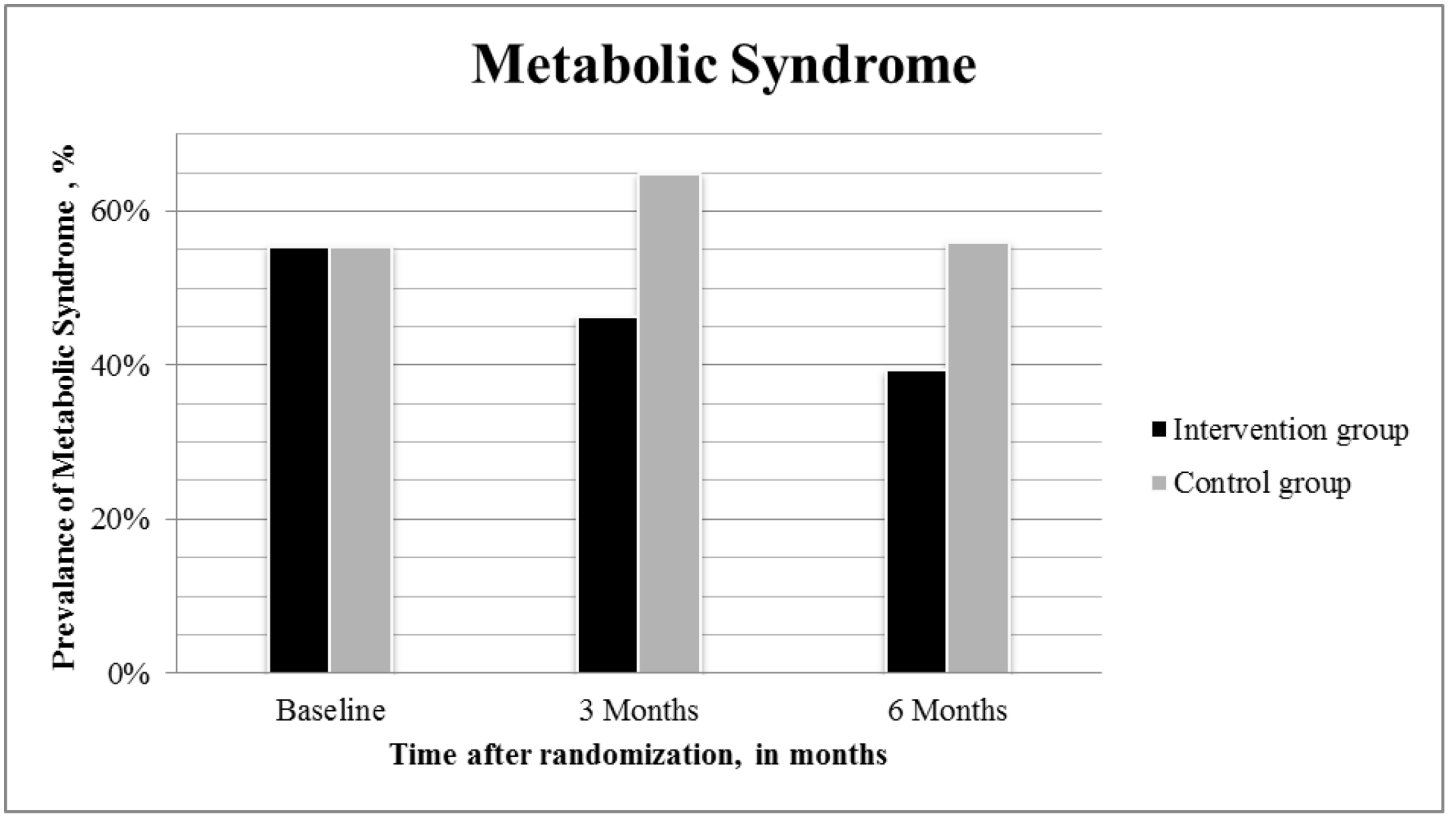
Prevalence of metabolic syndrome, at baseline, three and six months, by randomization group.

The prevalence of metabolic syndrome at baseline, three, and six months is shown in Fig 2. For women participating in the lifestyle intervention group, the odds ratio for metabolic syndrome was 0.53 (95% CI: 0.33 to 0.85; P < 0.01) compared to women in the control group, thus the probability of having metabolic syndrome was halved in the intervention group, compared to controls. The effect of the intervention on women with PCOS at baseline was not statistically different from women without PCOS at baseline.

**Fig 3:**
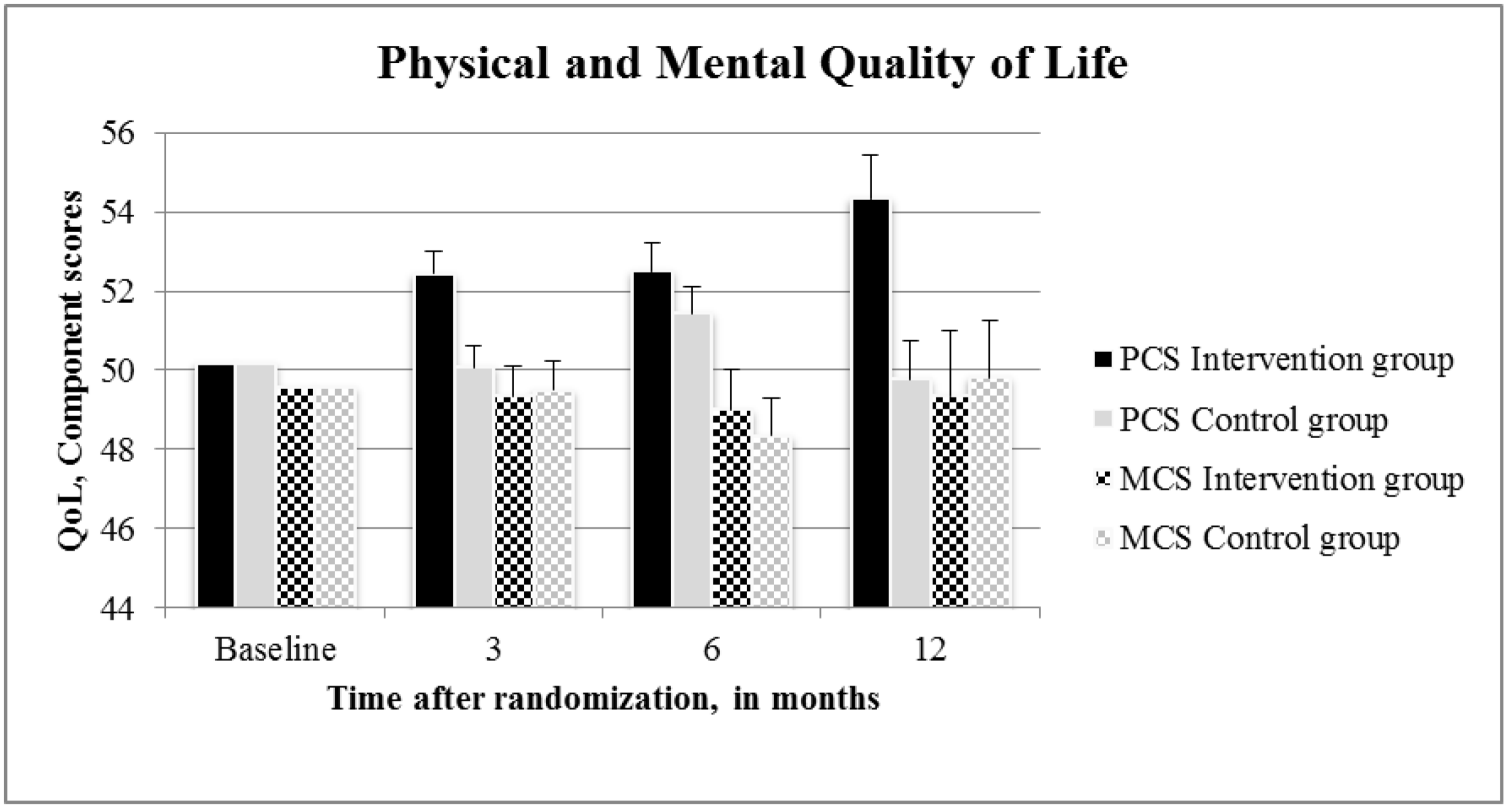
Physical and mental quality of life at baseline, three, six, and 12 months, by randomization group. **Abbreviations:** QoL, Quality of Life; PCS, Physical Component Score; MCS, Mental Component Score. Values are presented as mean±SE.

As shown in Fig 3, physical QoL scores were higher in the lifestyle intervention group, compared to the control group (2.2 95% CI: 0.9 to 3.5; P = 0.001). Mental QoL scores were not different between the groups. After we adjusted the mixed effect regression models for start of infertility treatment (Ovulation induction, intra uterine insemination, in vitro fertilization treatment, or intracytoplasmic sperm injection treatment) at either three or six months, the results did not change (data not shown). The mediation analyses showed that 24% (95% CI indirect effect: -0.9163 to -0.0754) of the total effect of the intervention on fasting insulin at three months was attributable to a decrease in sugary drinks, and 12% (95% CI indirect effect: -0.6885 to -0.0302) to a decrease in savory snacks. Of the total effect of the intervention on HOMA-IR at three months, 23% (95% CI indirect effect: -0.2528 to -0.0175) was attributable to a decrease in sugary drinks, and 12% (95% CI indirect effect: -0.1860 to -0.0087) to a decrease in savory snacks. No statistically significant mediators were found for other outcomes that were improved by the intervention (results in S2 Table).

## Discussion

A six month lifestyle intervention prior to infertility treatment improves cardiometabolic health in obese infertile women. Participants in the lifestyle intervention group had lower body weight, waist- and hip circumference, blood pressure, fasting glucose and insulin levels, insulin resistance, and a higher physical QoL compared to women who directly started infertility treatment. These relatively small but consistent effects on cardiometabolic factors resulted in halved odds of MetS.

The effect of the lifestyle intervention could be considered to have limited clinical relevance because of the modest effects on each of the separate outcomes. However, the effect of the intervention on the composite outcome for MetS is highly clinically relevant, since MetS leads to doubled risks of cardiovascular events and a 50% increase in all-cause mortality [8, 9, 39, 40]. Halving the odds of MetS could potentially greatly diminish the future cardiovascular risk of these women.

The intervention effects on cardiometabolic health were partly mediated by a reduction in the intake of sugary drinks and savory snacks. The reduced intake of sugary drinks improved insulin sensitivity, which can be explained by the association between sugar intake and insulin resistance [41]. Although increased physical activity has been associated with improved cardiometabolic outcomes, the intervention effects in the current study were not mediated by the change in physical activity [42, 43]. This is the first large multi-center RCT experimentally investigating the effects of a lifestyle intervention among obese infertile women. Previous studies in different populations have shown mixed effects of lifestyle interventions on cardiometabolic health. In contrast to our study, a recent meta-analysis including women with prior gestational diabetes showed that lifestyle interventions to prevent type 2 diabetes did not achieve weight-loss or improved fasting glucose at six months [44]. The reason an intervention effect was found in our study and was not found in the meta-analyses might be that the women in our trial were given an intervention that could improve their chances of reaching their primary goal; a healthy baby. Whereas, the women included in the meta-analysis did not have this strong intrinsic motivation. Another meta-analysis reported an intervention effect of -2.16 mmHg for systolic and -1.83 mmHg for diastolic blood pressure compared to usual care. Here, lifestyle intervention studies were included with a duration of at least 12 months. Participants were males and females with an average age of 53 years and a mean BMI of 30.5 kg/m2, without (pre)diabetes [45]. Although greater effects would be expected from lifestyle interventions of longer duration, our six month intervention led to similar lowering effects on systolic (-2.8 mmHg) and diastolic blood pressure (-1.7 mmHg). That comparable effects were achieved in only half of the time could be due to the fact that the women in our study were younger and more motivated to reduce weight in order to increase their chances on conception.

The control group also achieved some weight reduction. This might be due to the information given to all participants about the negative effects of obesity on fertility, as part of the usual care.

Approximately 35% of the participating women were diagnosed with PCOS [38]. PCOS often goes along with MetS, and both syndromes have a similar metabolic basis. Nevertheless, the increased cardiometabolic risks of PCOS are independent of but enhanced by adiposity [46]. Obese women with PCOS are more insulin resistant and at higher risk of cardiometabolic diseases including type 2 diabetes than obese women without PCOS [47, 48]. BMI is demonstrated to have a more potent extrinsic effect on insulin resistance in PCOS women compared to non-PCOS women [48]. Therefore, lifestyle interventions to reduce weight in obese women with PCOS seems even more important than in obese women without PCOS. In our study, similar intervention effects were found for women with as for women without PCOS.

Our finding of higher physical QoL is in concordance with previous weight loss trials [34]. The lack of effect of the intervention on mental QoL might be explained by the fact that women participating in this trial were infertile and their primary motivation for participating in the LIFEstyle study was to become pregnant and not to lose weight. For this reason ongoing infertility could overshadow the potential positive effect of the intervention on mental QoL [49].

Blinding of participants was not possible, because of the type of intervention. However, we consider it unlikely that the findings are unreliable due to bias as the biochemical outcomes were objectively measured, and the physical outcomes were assessed by research nurses not involved in the lifestyle intervention program.

The design of the current RCT has contributed to missing data (Fig 1), as participants who became pregnant during the first six months after randomization were excluded from further physical examination and blood sampling. Data collected during pregnancy were excluded from the statistical analyses because of the possible effects of pregnancy on the cardiometabolic health and QoL [50, 51]. Mixed effects regression model analyses were performed to deal with repeated measurements and missing data. This statistical method is able to accommodate all data available for a participant instead of excluding a participant from analysis in case of missing measurements [52].

Participants in the control group started infertility treatment directly, which might have affected cardiometabolic outcomes. However, after adjustment for infertility treatment, the effects of the lifestyle intervention on cardiometabolic health did not change. Hence, the effects can be attributed to the lifestyle intervention.

We have shown that a lifestyle intervention among obese infertile women improved cardiometabolic health. Based on these results obese infertile women should be informed about the positive effects of a lifestyle intervention on their cardiometabolic health and physical QoL. This may increase their intrinsic motivation to adjust lifestyle in the preconceptional period, apart from the increased chance of a natural conception and may facilitate sustained long-term lifestyle improvement [22]. Besides beneficial effects on health of the women, optimizing preconceptional lifestyle could lead to a healthier intrauterine environment, and improve long-term health in the offspring as well [53–55]. To evaluate the long-term effects of lifestyle intervention prior to infertility treatment, we are currently performing the 5–7 year follow-up of the LIFEstyle study in women and their offspring [56].

## Supporting information

S1 File Research protocol as approved by the ethics committee. (PDF)

S2 File Minimal data set. (SAV)

S1 Table CONSORT checklist. (PDF)

S2 Table Mediation analyses. Mediation analyses of change in physical activity and diet on outcomes that are improved by the intervention in comparison to the control group at three months based on primary mixed models analyses.
(DOC)

## References

[1] Xu J, Murphy S, Kochanek K, Bastian B. Deaths: Final data for 2013. National Vital Statistics Report. 2016;64(2). 26905861

[2] Collaboration NCDRF. Trends in adult body-mass index in 200 countries from 1975 to 2014: a pooled analysis of 1698 population-based measurement studies with 19·2 million participants. The Lancet. 2016;387(10026):1377–96. 10.1016/S0140-6736(16)30054-X. PMC7615134 27115820

[3] Gesink Law DC, Maclehose RF, Longnecker MP. Obesity and time to pregnancy. Human Reproduction. 2007;22(2):414–20. 10.1093/humrep/del400 17095518 PMC1924918

[4] van der Steeg JW, Steures P, Eijkemans MJC, Habbema JDF, Hompes PGA, Burggraaff JM, et al Obesity affects spontaneous pregnancy chances in subfertile, ovulatory women. Human Reproduction. 2008;23(2):324–8. 10.1093/humrep/dem371 18077317

[5] Brown CD, Higgins M, Donato KA, Rohde FC, Garrison R, Obarzanek E, et al Body Mass Index and the Prevalence of Hypertension and Dyslipidemia. Obesity research. 2000;8(9):605–19. 10.1038/oby.2000.79 11225709

[6] Rocha VZ, Libby P. Obesity, inflammation, and atherosclerosis. Nat Rev Cardiol. 2009;6(6):399–409. 10.1038/nrcardio.2009.55 19399028

[7] Expert Panel on D, Evaluation, and Treatment of High Blood Cholesterol in A. EXecutive summary of the third report of the national cholesterol education program (ncep) expert panel on detection, evaluation, and treatment of high blood cholesterol in adults (adult treatment panel iii). JAMA. 2001;285(19):2486–97. 10.1001/jama.285.19.2486 11368702

[8] Grundy SM, Cleeman JI, Daniels SR, Donato KA, Eckel RH, Franklin BA, et al Diagnosis and Management of the Metabolic Syndrome: An American Heart Association/National Heart, Lung, and Blood Institute Scientific Statement. Circulation. 2005;112(17):2735–52. 10.1161/CIRCULATIONAHA.105.169404 16157765

[9] Mottillo S, Filion KB, Genest J, Joseph L, Pilote L, Poirier P, et al The Metabolic Syndrome and Cardiovascular Risk: A Systematic Review and Meta-Analysis. Journal of the American College of Cardiology. 2010;56(14):1113–32. 10.1016/j.jacc.2010.05.034 20863953

[10] Malik S, Wong ND, Franklin SS, Kamath TV, L’Italien GJ, Pio JR, et al Impact of the Metabolic Syndrome on Mortality From Coronary Heart Disease, Cardiovascular Disease, and All Causes in United States Adults. Circulation. 2004;110(10):1245–50. 10.1161/01.CIR.0000140677.20606.0E 15326067

[11] Han JH, Park HS, Shin CI, Chang HM, Yun KE, Cho SH, et al Metabolic syndrome and quality of life (QOL) using generalised and obesity-specific QOL scales. International journal of clinical practice. 2009;63(5):735–41. Epub 2009/04/28. 10.1111/j.1742-1241.2009.02021.x . 19392923

[12] Hahn S, Janssen OE, Tan S, Pleger K, Mann K, Schedlowski M, et al Clinical and psychological correlates of quality-of-life in polycystic ovary syndrome. European Journal of Endocrinology. 2005;153(6):853–60. 10.1530/eje.1.02024 16322391

[13] Otero-Rodriguez A, Leon-Munoz LM, Balboa-Castillo T, Banegas JR, Rodriguez-Artalejo F, Guallar-Castillon P. Change in health-related quality of life as a predictor of mortality in the older adults. Quality of life research: an international journal of quality of life aspects of treatment, care and rehabilitation. 2010;19(1):15–23. Epub 2009/12/01. 10.1007/s11136-009-9561-4 . 19946754

[14] Apovian CM, Aronne LJ, Bessesen DH, McDonnell ME, Murad MH, Pagotto U, et al Pharmacological Management of Obesity: An Endocrine Society Clinical Practice Guideline. The Journal of Clinical Endocrinology & Metabolism. 2015;100(2):342–62. 10.1210/jc.2014-3415 . 25590212

[15] Lloyd-Jones DM, Hong Y, Labarthe D, Mozaffarian D, Appel LJ, Van Horn L, et al Defining and Setting National Goals for Cardiovascular Health Promotion and Disease Reduction. The American Heart Association’s Strategic Impact Goal Through 2020 and Beyond. 2010;121(4):586–613. 10.1161/circulationaha.109.192703 20089546

[16] Wu T, Gao X, Chen M, Van Dam RM. Long-term effectiveness of diet-plus-exercise interventions vs. diet-only interventions for weight loss: a meta-analysis. Obesity Reviews. 2009;10(3):313–23. 10.1111/j.1467-789X.2008.00547.x 19175510

[17] Orozco LJ, Buchleitner AM, Gimenez-Perez G, Roqué i Figuls M, Richter B, Mauricio D. Exercise or exercise and diet for preventing type 2 diabetes mellitus. Cochrane Database of Systematic Reviews. 2008;(3). 10.1002/14651858.CD003054.pub3 18646086

[18] Roumen C, Blaak EE, Corpeleijn E. Lifestyle intervention for prevention of diabetes: determinants of success for future implementation. Nutrition Reviews. 2009;67(3):132 10.1111/j.1753-4887.2009.00181.x 19239628

[19] Messina J, Campbell S, Morris R, Eyles E, Sanders C. A narrative systematic review of factors affecting diabetes prevention in primary care settings. PLoS ONE. 2017;12(5):e0177699 10.1371/journal.pone.0177699 28531197 PMC5439678

[20] Herzig K, Danley D, Jackson R, Petersen R, Chamberlain L, Gerbert B. Seizing the 9-month moment: Addressing behavioral risks in prenatal patients. Patient Education and Counseling. 2006;61(2):228–35. 10.1016/j.pec.2005.04.001 16256291

[21] McBride CM, Emmons KM, Lipkus IM. Understanding the potential of teachable moments: the case of smoking cessation. Health Education Research. 2003;18(2):156–70. 10.1093/her/18.2.156 12729175

[22] Mutsaerts MAQ, van Oers AM, Groen H, Burggraaff JM, Kuchenbecker WKH, Perquin DAM, et al Randomized Trial of a Lifestyle Program in Obese Infertile Women. New England Journal of Medicine. 2016;374(20):1942–53. 10.1056/NEJMoa1505297 . 27192672

[23] Mutsaerts M, Groen H, ter Bogt N, Bolster J, Land J, Bemelmans W, et al The LIFESTYLE study: costs and effects of a structured lifestyle program in overweight and obese subfertile women to reduce the need for fertility treatment and improve reproductive outcome. A randomised controlled trial. BMC Women’s Health. 2010;10(1):22 10.1186/1472-6874-10-22 20579357 PMC2907305

[24] Dhont M. WHO-classification of anovulation: background, evidence and problems. International Congress Series. 2005;1279:3–9. 10.1016/j.ics.2004.12.028.

[25] Voedingscentrum. Mijn eetmeter: scan je dagmenu. (https://mijn.voedingscentrum.nl/nl/eetmeter/).

[26] Clinical Guidelines on the Identification, Evaluation, and Treatment of Overweight and Obesity in Adults—The Evidence Report. National Institutes of Health. Obesity research. 1998;6 Suppl 2:51s–209s. Epub 1998/11/14 . 9813653

[27] Patrick K SJ, Long B, Calfas KJ, Wooten W, Heath G, Pratt M. A new tool for encouraging activity. Project PACE. The physician and sportsmedicine. 1994:45–55. 10.1080/00913847.1994.11947706 29275663

[28] ter Bogt NC, Bemelmans WJ, Beltman FW, Broer J, Smit AJ, van der Meer K. Preventing weight gain: one-year results of a randomized lifestyle intervention. American journal of preventive medicine. 2009;37(4):270–7. Epub 2009/09/22. 10.1016/j.amepre.2009.06.011 . 19765497

[29] NVOG. DSoOaG, Data sheet. http://nvog-documenten.nl/index.php?pagina=/richtlijn/pagina.php&fSelectNTG_112=113&fSelectedSub=112).

[30] Consensus on infertility treatment related to polycystic ovary syndrome. Human Reproduction. 2008;23(3):462–77. 10.1093/humrep/dem426 18308833

[31] Hunault CC, Habbema JDF, Eijkemans MJC, Collins JA, Evers JLH, te Velde ER. Two new prediction rules for spontaneous pregnancy leading to live birth among subfertile couples, based on the synthesis of three previous models. Human Reproduction. 2004;19(9):2019–26. 10.1093/humrep/deh365 15192070

[32] Matthews DR H J, Rudenski AS, Naylor BA, Treacher DF, Turner RC. Homeostasis model assessment: insulin resistance and beta-cell function from fasting plasma glucose and insulin concentrations in man. Diabetologia. 1985:412–9. 3899825 10.1007/BF00280883

[33] Aaronson NK, Muller M, Cohen PD, Essink-Bot ML, Fekkes M, Sanderman R, et al Translation, validation, and norming of the Dutch language version of the SF-36 Health Survey in community and chronic disease populations. Journal of clinical epidemiology. 1998;51(11):1055–68. Epub 1998/11/17. . 9817123 10.1016/s0895-4356(98)00097-3

[34] Danielsen KK, Sundgot-Borgen J, Maehlum S, Svendsen M. Beyond weight reduction: improvements in quality of life after an intensive lifestyle intervention in subjects with severe obesity. Annals of medicine. 2014;46(5):273–82. Epub 2014/02/05. 10.3109/07853890.2013.874660 . 24491067

[35] VanderZee KI, Sanderman R, Heyink J. A comparison of two multidimensional measures of health status: the Nottingham Health Profile and the RAND 36-Item Health Survey 1.0. Quality of life research: an international journal of quality of life aspects of treatment, care and rehabilitation. 1996;5(1):165–74. Epub 1996/02/01. . 8901380 10.1007/BF00435982

[36] Wendel-Vos GCW, Schuit AJ, Saris WHM, Kromhout D. Reproducibility and relative validity of the short questionnaire to assess health-enhancing physical activity. Journal of clinical epidemiology. 2003;56(12):1163–9. 10.1016/S0895-4356(03)00220-8 14680666

[37] AF H. Introduction to mediation, moderation, and conditional process analysis: a regression-based approach: New York: Guilford; 2013.

[38] Revised 2003 consensus on diagnostic criteria and long-term health risks related to polycystic ovary syndrome. Fertility and sterility. 2004;81(1):19–25. 10.1016/j.fertnstert.2003.10.004 14711538

[39] Cerezo C, Segura J, Praga M, Ruilope LM. Guidelines Updates in the Treatment of Obesity or Metabolic Syndrome and Hypertension. Current Hypertension Reports. 2013;15(3):196–203. 10.1007/s11906-013-0337-4 23519746

[40] National Guideline C. Cardiometabolic risk management guidelines in primary care. 2011.

[41] Schulze MB, Manson JE, Ludwig DS, et al Sugar-sweetened beverages, weight gain, and incidence of type 2 diabetes in young and middle-aged women. JAMA. 2004;292(8):927–34. 10.1001/jama.292.8.927 15328324

[42] Chomistek AK, Manson JE, Stefanick ML, Lu B, Sands-Lincoln M, Going SB, et al Relationship of Sedentary Behavior and Physical Activity to Incident Cardiovascular Disease: Results From the Women’s Health Initiative. Journal of the American College of Cardiology. 2013;61(23):2346–54. 10.1016/j.jacc.2013.03.031 23583242 PMC3676694

[43] Bird SR, Hawley JA. Update on the effects of physical activity on insulin sensitivity in humans. BMJ Open Sport & Exercise Medicine. 2017;2(1). 10.1136/bmjsem-2016-000143 PMC5569266 28879026

[44] Gilinsky AS, Kirk AF, Hughes AR, Lindsay RS. Lifestyle interventions for type 2 diabetes prevention in women with prior gestational diabetes: A systematic review and meta-analysis of behavioural, anthropometric and metabolic outcomes. Preventive medicine reports. 2015;2:448–61. Epub 2016/02/05. 10.1016/j.pmedr.2015.05.009 . 26844102 PMC4721374

[45] Zhang X, Devlin HM, Smith B, Imperatore G, Thomas W, Lobelo F, et al Effect of lifestyle interventions on cardiovascular risk factors among adults without impaired glucose tolerance or diabetes: A systematic review and meta-analysis. PLOS ONE. 2017;12(5):e0176436 10.1371/journal.pone.0176436 28493887 PMC5426619

[46] Moran LJ, Misso ML, Wild RA, Norman RJ. Impaired glucose tolerance, type 2 diabetes and metabolic syndrome in polycystic ovary syndrome: a systematic review and meta-analysis. Human Reproduction Update. 2010;16(4):347–63. 10.1093/humupd/dmq001 20159883

[47] Moran LJ, Norman RJ, Teede HJ. Metabolic risk in PCOS: phenotype and adiposity impact. Trends in Endocrinology & Metabolism. 2015;26(3):136–43. 10.1016/j.tem.2014.12.003. 25591984

[48] Stepto NK, Cassar S, Joham AE, Hutchison SK, Harrison CL, Goldstein RF, et al Women with polycystic ovary syndrome have intrinsic insulin resistance on euglycaemic—hyperinsulaemic clamp. Human Reproduction. 2013;28(3):777–84. 10.1093/humrep/des463 23315061

[49] Direkvand-Moghadam A, Delpisheh A, Direkvand-Moghadam A. Effect of Infertility on the Quality of Life, A Cross- Sectional Study. Journal of Clinical and Diagnostic Research: JCDR. 2014;8(10):OC13–OC5. 10.7860/JCDR/2014/8481.5063 25478412 PMC4253230

[50] Sanghavi M, Rutherford JD. Cardiovascular Physiology of Pregnancy. Circulation. 2014;130(12):1003–8. 10.1161/CIRCULATIONAHA.114.009029 25223771

[51] Sonagra AD, Biradar SM, K D, Murthy DSJ. Normal Pregnancy- A State of Insulin Resistance. Journal of Clinical and Diagnostic Research: JCDR. 2014;8(11):CC01–CC3. 10.7860/JCDR/2014/10068.5081 25584208 PMC4290225

[52] West BT. Analyzing Longitudinal Data With the Linear Mixed Models Procedure in SPSS. Evaluation & the Health Professions. 2009;32(3):207–28. 10.1177/0163278709338554 19679634

[53] Barker DJP, Osmond C. INFANT MORTALITY, CHILDHOOD NUTRITION, AND ISCHAEMIC HEART DISEASE IN ENGLAND AND WALES. The Lancet. 1986;327(8489):1077–81. 10.1016/S0140-6736(86)91340-1. 2871345

[54] Whincup PH, Kaye SJ, Owen CG, et al Birth weight and risk of type 2 diabetes: A systematic review. JAMA. 2008;300(24):2886–97. 10.1001/jama.2008.886 19109117

[55] Gluckman PD, Hanson MA. Living with the Past: Evolution, Development, and Patterns of Disease. Science. 2004;305(5691):1733 10.1126/science.1095292 15375258

[56] van de Beek C, Hoek A, Painter R, Gemke R, van Poppel M, Geelen A, et al Women, their Offspring and iMproving lifestyle for Better cardiovascular health of both (WOMB project): a protocol of the follow-up of a multicentre randomized controlled trial. BMJ open 2017, Accepted for publication. 10.1136/bmjopen-2017-016579 PMC5786127 29371262

