# Supplementary material for: Effect of a lifestyle intervention in obese infertile women on cardiometabolic health and quality of life: A randomized controlled trial
Source: PLoS One. 2018 Jan 11;13(1):e0190662. doi: 10.1371/journal.pone.0190662 (PMC5764284; doi:10.1371/journal.pone.0190662)
Supplement: S1 File — (PDF) [file pone.0190662.s001.pdf]

RESEARCH PROTOCOL

Costs and effects of a structured lifestyle program in overweight and obese subfertile couples to prevent unnecessary treatment and improve reproductive outcome

PROTOCOL TITLE

Costs and effects of a structured lifestyle program in overweight and obese subfertile couples to prevent unnecessary treatment and improve reproductive outcome

Protocol ID	ZonMW 50-50110-97-518
Short title	Women of Weight and Infertility
Version	1
Date	21-07-08
/project leader	*Dr. A. Hoek MD*<br>*Section of Reproductive Medicine*<br>*Dept. Obstetrics and Gynaecology UMCG*<br>*Hanzeplein 1*<br>*9700 RB Groningen*<br>***a.hoek@og.umcg.nl***<br>*T:050-3613152*<br>*F:050-3611806*
Principal investigator(s)<br>(*Multicenter research: per site)*	*Dr. A. Hoek MD*<br><br>*Prof dr. JA. Land (UMC Groningen)*<br>*Dr. H. Groen (Epidemiology, UMC Groningen)*<br>*Prof Dr. R.P. Stolk (Epidemiology UMC Groningen)*<br>*Dr. Ir. W. Bemelmans (RIVM Bilthoven)*<br>*Dr. J.W.M. Maas (UMC Maastricht)*<br>*Prof dr. F.M. Helmerhorst (UMC Leiden)*<br>*Dr. P.G.A. Hompes (Free UMC Amsterdam)*<br>*Prof. dr N.S. Macklon (UMC Utrecht)*<br>*Prof dr. J.A.M. Kremer (UMC Nijmegen)*

	Prof. dr. F. van der Veen (AMC Amsterdam)<br><br>*Prof dr. B.W. Mol (Maxima Medical Center Velthoven)*<br>*Dr. E.M. Kaaijk (OLVG Amsterdam)*<br>*Dr. G.J.E. Oosterhuis (Medisch Spectrum Twente Enschede)*<br>*Dr. P.X.J.M. Bouckaert (Atrium Medisch Centrum Heerlen)*<br>*Dr. P.J.Q. van der Linden (Deventer Ziekenhuis Deventer)*<br>*Dr. Y. M. van Kasteren (Medische centrum Alkmaar)*<br>*Drs. W.K.H. Kuchenbecker (Isala klinieken Zwolle)*<br>*Dr. L.F.M.M. Bancsi, (Ziekenhuis Rijnstate Arnhem)*<br>*Dr. JS.E. Laven, (Erasmus MC Rotterdam)*<br>*Dr. E.A. Brinkhuis, (MeanderMC, Amersfoort)*<br>*Drs. J.M. Burggraaff (Leveste ziekenhuis, Emmen)*
Sponsor	*ZonMW*<br>*Laan van Nieuw Oost Indië 334*<br>*2593 CE Den Haag*<br>*T: 07-3495111*
Independent physician(s)	*Drs. D.H. Bogchelman*<br>*Dept. Obstetrics and Section Gynaecology UMCG*<br>*Hanzeplein 1*<br>*9700RB Groningen*<br>*d.h.Bogchelman@og.UMCG.nl*

PROTOCOL SIGNATURE SHEET

Name	Signature	Date
Coordinating Investigator/Project leader/<br>Dr. A. Hoek MD, PhD.<br>Gynaecologist UMCG	Dr. A. Hoek MD PhD.<br>Gynaecologist UMCG	30-1-09

TABLE OF CONTENTS

1. INTRODUCTION AND RATIONALE 10
2. OBJECTIVES 12
3. STUDY DESIGN 13
4. STUDY POPULATION 13
4.1 Population (base) 13
4.2 Inclusion criteria 13
4.3 Exclusion criteria 13
4.4 Sample size calculation 13
5. TREATMENT OF SUBJECTS 14
5.1 Investigational product/treatment 14
6 METHODS 17
6.1 Study parameters/endpoints 17
6.1.1 Main study parameter/endpoint 17
6.1.2 Secondary study parameters/endpoints (if applicable) 17
6.1.3 Other study parameters (if applicable) 17
6.2 Randomisation, blinding and treatment allocation 18
6.3 Study procedures 18
6.4 Withdrawal of individual subjects 22
7. SAFETY REPORTING 22
7.1 Section 10 WMO event 22
7.2 Adverse and serious adverse events 23
7.3 Follow-up of adverse events 23
8 STATISTICAL ANALYSIS 23
8.1 Descriptive statistics 23
8.2 Univariate analysis 24
8.3 Multivariate analysis 24
8.4 Economic evaluation 24
9. ETHICAL CONSIDERATIONS 25
9.1 Regulation statement 26
9.2 Recruitment and consent 26
9.3 Objection by minors or incapacitated subjects (if applicable) 27
9.4 Benefits and risks assessment, group relatedness 27
9.5 Compensation for injury 27
10. ADMINISTRATIVE ASPECTS AND PUBLICATION 28
10.1 Handling and storage of data and documents 28
10.2 Amendments 28
10.3 Annual progress report 28
10.4 End of study report 29
11. REFERENCES and addendum 29-38

LIST OF ABBREVIATIONS AND RELEVANT DEFINITIONS

ABR	ABR form (General Assessment and Registration form) is the application form that is required for submission to the accredited Ethics Committee (ABR = Algemene Beoordeling en Registratie)
AE	Adverse Event
BMI	Body mass index
CA	Competent Authority
CBO	Centrale begeleidingsorgaan
CCMO	Central Committee on Research Involving Human Subjects
COH	Controlled ovarian hyperstimulation
CV	Curriculum Vitae
DEBQ	Dutch eating behaviour questionnaire
EU	European Union
FFQ	Food frequency questionnaire
FSH	follicle-stimulating hormone
FS-36	
GCP	Good clinical practice
GGD	Gemeentelijke Gezondheidsdienst
GOAL	Groninger Overweight And Lifestyle study
HCG	Human Choriongonadotrofine
HELLP	Syndrome with Haemolysis, Elevated Liver Enzymes and Low Platelets
HSG	Hysterosalpingography
HOMA	Homeostasis Model Assessment (measuring Insuline resisance)
IC	Informed Consent
ICSI I	Intra cytoplasmic sperm injection
RB	Institutional review board
IUI	Intra-uterine insemination
IVF-ET	In vitro fertilisation and embryo transfer
KCal	Kilo Calory
LH	Luteinizing hormone
METC	Medisch ethische toetsing commissie
NICE	Natinal Institute of Clinical Excellence UK
NIH	National Institute of Health USA
NVOG	Nederlanse Vereniging voor Obstetrie en Gynaecologie

OI	Ovulation Induction
PAM	Physical activity meter
PCOS	Polycystic ovary syndrome
RDC	Remote Data Capture
SMB	Safety Monitoring Board
(S)AE	Serious Adverse Event
SHBG	Sexhormone binding globulin
SUSAR	Suspected Unexpected Serious Adverse Reaction
TSH	Thyroid stimulating hormone
Wbp	Personal Data Protection Act (in Dutch: Wet Bescherming Persoonsgevens)
Wc	Waist circumference
WHO	World health organization
WHR	Waist hip ratio
WMO	Wet medisch onderzoek

SUMMARY

Rationale: Subfertility affects approximately one in ten couples planning conception. Among subfertile women, about 30% are overweight or even obese. Epidemiological data suggest that the reduction of overweight will increase the chances of conception, decrease pregnancy complications and improve perinatal outcome. In small intervention studies beneficial reproductive effects of improvement of lifestyle leading to a reduction in body weight have been demonstrated. The British Fertility Society advises that fertility treatment be withheld if the body mass index (BMI) is over 35.
In the Netherlands, there is at present no agreed standard of care for subfertile women with overweight or obesity. Some centres simply withhold treatment of couples in whom the woman is overweight or obese, but most fertility centres treat overweight or obese women irrespective of their BMI. In a few centres, support is offered to women to help them lose weight.

Objective: In view of this lack of evidence and strong practice variation we propose a randomized clinical trial in overweight and obese subfertile women, in which we compare the costs and effects of a six months structured lifestyle program, aimed at weight loss, to “usual care”. The intervention aims to prevent unnecessary fertility treatment, complications associated with fertility treatment and obesity related pregnancy complications, thus improving pregnancy chances and perinatal outcome.

Study design: Multicenter randomised clinical trial.

Study population: Subfertile women (age 18-38) with a BMI between 29 and 40. Exclusion criteria are: azoospermia, severe endometriosis, chronic anovulation due to WHO III and endocrinopathies. Women with preexistent hypertension or diabetes, or pregnancy induced hypertension, preeclampsia, eclampsia or HELLP syndrome in a previous pregnancy are also excluded.

Intervention: The intervention consists of a structured lifestyle program of six months, which aims at realistic weight loss of at least 5% to10%, achieved by the combination of a healthy diet (caloric reduction of 600kCal/day), increase of physical activity (aiming at 10.000 steps per day) and two to three times a week sporting activity, and behavioural modification. The structured lifestyle program, in which practice variation is minimized using a structured software program, has been developed, used and evaluated previously (Zon-MW project 50-50110-98-078; the Groninger Overweight And Lifestyle study (GOAL)). Following six months of lifestyle intervention patients will start fertility treatment as indicated in the “usual care” group.
In the “usual care” arm fertility treatment will be started if this is justified by the individual prognosis (guideline NVOG)
All randomised women will fill in questionnaires concerning: diet, eating behaviour, physical activity and smoking: SF-36, FFQ, DEBQ, Squash list, at inclusion, and at 12 weeks, 6 months, 12 months and 24 months after randomisation. Blood samples (10 cc) will be taken at the start, 3 months and 6 months after the start of the study

Main study parameters/endpoints:
Primary endpoint is the birth of a healthy singleton after vaginal delivery of at least 37 weeks gestation.
Secondary outcome parameters are number of fertility treatments (OI, IUI, IVF, ICSI) , clinical and ongoing pregnancy rates, perinatal outcome, complications and quality of life as well as body weight, waist circumference, behaviour influencing weight, i.e. nutritional habits and exercise pattern, smoking habits, blood pressure, glucose/insulin ratio (HOMA), hormonal profile and costs.

DATA ANALYSIS: Analysis will be by intention to treat. Randomisation will be stratified for the presence or absence of anovulation and per treatment center. We expect an improvement in vaginal singleton delivery beyond 37 weeks from 45% to 60%. Anticipating drop out of 20% in the intervention arm ad a 5% loss to follow up in the study, we need to include two groups of 285 women (two-sided test, alpha error.05, beta-error .20).

STUDY DURATION: Preparation of the study, including the training of the nurses, will take three months. Inclusion of the couples will take 24 months and the lifestyle intervention will take six months. Follow up is continued for 24 months after randomisation. Database cleaning and analysis of the study will take 6 months. Duration of the study: 57 months

Nature and extent of the burden and risks associated with participation, benefit and group relatedness:

The advantages of a weight loss and exercise program in overweight or obese subfertile women have been shown in small intervention studies. Even if the patient does not conceive during the 6 months period in the lifestyle intervention arm, the health benefits of weight loss on the long-term are clearly in the interest of the patient.

By starting this project, fertility treatment in the intervention arm will be delayed for a maximum of 6 months, there is no evidence that this delay will cause lower spontaneous or treatment dependent pregnancy chances. A more structured weight loss and exercise program will probably lead to higher spontaneous and therapy induced ongoing pregnancy chances.

The Burden: Patients that are randomized in the intervention arm will attend the clinic for six extra visits in order to improve lifestyle, this will take around 280 minutes. In between visits counselling will take place per telephone or mail four times for 15 minutes. During these visits physical examination, measuring weight, WC and WHR in week: one, three, seven, twelve, eighteen and twenty-four will take place. Patients in the intervention group will have to adapt there lifestyle towards a more healthy lifestyle, this will give a certain amount of stress since they will have to adapt their diet and physical activity. Patients will be learned how to use stepcounter PAM and wear it on a daily basis in order to monitor their physical activity during the intervention period and will fill in their activity list.

Patients who have been randomized in the "usual care" group will undergo fertility treatment when indicated by national guidelines (www.nvog.nl). Patients with overweight or obesity have additional risks during pregnancy compared to normal weight women. The risks involve: hypertension (OR: 2.2), preeclampsia (OR: 1.8-2.7), pregnancy-induced diabetes (OR: 2.2-3.4), caesarian section (OR: 1.3-2.4) (see tables of evidence in appendix). These risks are not influenced by fertility treatment. Lifestyle intervention aims to reduce the involved pregnancy risks.

An independent Safety Monitoring Board (SMB) will be installed to review major complications in both treatment groups. This board will evaluate complications after every 150 included patients and six months and 12 months after the end of inclusion of the study (6 times during the study). The SMB will differentiate between major en minor complications, blinded for treatment arm. Every case of preeclampsia, eclampsia, HELLP syndrome, maternal or infant mortality will be reported to the SMB. The SMB findings will be reported to the Ethics Committee of the UMCG

Questionnaires to be filled in by all participants in the study: SF-36, SQUASH-list, FFQ, DEBQ, concerning eating behaviour at the start, week twelve, twenty-four and fifty-two of the study. A questionnaire for details on direct and indirect costs like transportation, sporting activity and productivity loss due to lifestyle intervention programme and fertility treatment will be filled in by the participants during the study. This will take 30-45 minutes.

1. INTRODUCTION AND RATIONALE

The evidence for the adverse effects of overweight on women's reproductive health is indisputable. Overweight affects reproductive capacity in all subfertile couples. Moreover, overweight is associated with pregnancy complications and poor neonatal outcome. Pregnancy complications associated with obesity are hypertensive disorders, gestational diabetes, prolonged duration of labour, increased need of operative delivery, macrosomia, shoulder dystocia and increased blood loss (Garbaciak et al.,1985; Edwards et al.,1996). Obesity is associated with an increased risk of adverse pregnancy outcomes such as unexplained still birth (Cnattingius et al.,1998; Kristensen et al.,2005; Bergstrom et al.,1998; Linne et al.,2004, neonatal admissions (Usha Kiran et al.,2005) and five times higher costs (Linne et al.,2004).

Each year about 15.000 to 20.000 new couples present themselves with subfertility. In a large Dutch cohort study of 3.000 subfertile couples (Van der Steeg et al.,2007), 19% of the women had a BMI between 25 and 30, 7% had a BMI between 30 and 35, and 4% above 35. Thus, approximately 30% of these couples are overweight or obese.

Obesity has further been associated with reduced fertility in the general population (Ramlau-Hansen et al.,2007). Ovulatory subfertile women with a BMI of 29 kg/m2 or higher have a 4% lower pregnancy rate per kg/m^2 increase (Van der Steeg et al., 2007). Obese women also have a lower live birth rate after IVF and ICSI (Norman et al.,1998; Wang et al.,2000; Wang et al.,2002; Fedorcsak et al.,2004, Lintsen et al.,2005).
The relationship between BMI and the risk of spontaneous abortion is inconsistent (Lashen et al.,1999; Fedorczak et al.,2000; Wittemer et al.,2000; Wang et al.,2001, Lintsen et al.,2005). A meta-analysis on the effect of overweight and obesity on ART reported a lower chance of pregnancy following IVF (OR 0.71, 95% CI:0.620-0.81) and an increased miscarriage rate (OR 1.3, 95% CI:1.06-1.68). Extrapolating from available data one could argue that the combination of lower chance of pregnancy and higher miscarriage rate in overweight and obese women could result in a reduced live birth rate (Maheshwari et al.,2007).

Obesity is strongly related to polycystic ovary syndrome (PCOS), which is the cause of subfertility in 25% couples. The accompanying insulin resistance and hyperinsulinism mark this syndrome as a prediabetic state (Dunaif, 1997), leading to an increase of neonatal complications.

In reproductive medicine, modest weight reduction (5-10%) with lifestyle intervention is not only associated with restoration of ovulation and fertility in overweight women with PCOS (Hoeger et al.,2004) but is also shown to decrease insulin resistance (Pasquali et al.,1989; Knowles et al.2002). Several studies have shown that even a modest weight loss leading to a 5% loss of central fat, in women with PCOS improves, the menstrual cycle, the rate of ovulation, and the likelihood of a healthy pregnancy (Clark et al.,1995, Norman et al.,2004, Huber-Buchholz et al,.1999, Stamets et al., 2004).

In view of the evidence that overweight has a negative impact on fertility and pregnancy outcome, lifestyle intervention prior to treatment of subfertile couples could improve spontaneous fertility chances, and prevent unnecessary fertility treatment as well as complications of fertility treatment.

Several reviews showed that lifestyle interventions targeted at changing diet and physical activity can succeed in sustained decrease of average bodyweight on the longer term (McTigue, 2004; Franz 2007). A decrease of 3 to 4 kilograms can be achieved after three years of follow-up (Avenell, 2004). The adverse health effects of increased body weight on for instance development of diabetes mellitus type 2, coronary heart disease, musculoskeletal disorders and some types of cancer are well known (WHO-euro document 2007). The cost-effectiveness of lifestyle interventions was below acceptable limits (Vijgen, 2006). For the Diabetes prevention project, for instance, the costs to prevent one new case of diabetes mellitus were calculated to be around 16.000 US dollars (Herman, 2005).

The intervention we propose to evaluate is based on dietary therapy, increased physical activity and individualized behavioural modification plan. A combination of these three interventions leads to maximal weight loss and maintenance of weight loss (NIH guideline, CBO guideline 2007, Zelissen et al 2004). This behavioural modification therapy can be delegated to a nurse practitioner or trained nurse or dietician. Such an intervention program can be implemented within gynaecological care.

Although lifestyle modification is advised as a key component for the improvement of reproductive function in overweight women specifically with PCOS (NICE 2004, Balen et al., 2006 Tang et al.,2006, Pasquali et al.,1989, Hoeger et al.,2004, Clark et al.,1995 and 1998), the evidence of its effectiveness as demonstrated in clinical studies is limited. It's cost-effectiveness has never been assessed in large groups of subfertile women with respect to prevention of unnecessary fertility treatment, prevention of complications of fertility treatment and improvement of perinatal outcome.

The cost-effectiveness of such program will be investigated in the this study.
(see table Summary of studies assessing outcome after lifestyle intervention).

2. OBJECTIVES

In view of the lack of convincing evidence from large intervention studies and the strong practice variation in The Netherlands we propose a randomized clinical trial in overweight and obese subfertile women, in which we compare the costs and effects of a six months structured lifestyle program, aimed at weight loss, to "usual care" with the aim to prevent obesity related pregnancy complications, unnecessary fertility treatment and complications associated with fertility treatment, enhance pregnancy chances and improve perinatal outcome. The control group of women will receive fertility treatment "care as usual" without lifestyle intervention.

We aim to estimate the costs and effects of a structured lifestyle program in terms of costs per additional healthy singleton birth rate beyond 37 weeks gestation born after vaginal delivery in a follow up period of 24 months.

The questions that we aim to answer are:

Primary Objective:
Does a structured lifestyle program aiming at a weight reduction of 5%-10% reduce the need for fertility treatments due to an increase in spontaneous conception rates?

Secondary Objective(s):
Does a structured lifestyle program aiming at a weight reduction of 5%-10% increases the success rates of fertility treatments, including assisted reproductive technology?

Does a structured lifestyle program aiming at a weight reduction of 5%-10%, lead to less maternal complications during pregnancy, such as hypertensive disorders, premature birth, macrosomia, shoulder dystocia, operative delivery, and haemorraghia, and multiple pregancies in subfertile women with overweight or obesity?

Does a structured lifestyle program aiming at a weight reduction of 5%-10% lead to a lower percentage of women with overweight or obesity and subfertility developing gestational diabetes?

Does a structured lifestyle program aiming at a weight reduction of 5%-10% in subfertile women, lead to a better quality of life, as well as body weight reduction, reduction of waist circumference, behaviour influencing weight, i.e. nutritional habits and exercise pattern, blood pressure, serum glucose/insulin ratio (HOMA) and hormonal profile changes.

3. STUDY DESIGN

Multicenter randomised clinical trial.
Flow chart 1 gives an overview of the study procedure form randomization till end of follow up period.
Table 1 is added to give a overview of the lifestyle intervention that subjects will undergo in the course of research.

STUDY MANAGEMENT: The randomisation is performed by computer at a central randomisation centre in the AMC. Randomisation will be stratified according presence or absence of anovulation and center of treatment.
Data will be collected using Oracle Clinical Remote Data Capture (RDC), which is a new generation of application system that enables collection and cleanup of clinical trial data using the Internet. For detailed information on Oracle RDC, please visit the page of Oracle RDC products (http://www.ctc-g.co.jp/en). The expertise for this technology is already used in the study group.

4. STUDY POPULATION

4.1 Population (base)

All couples suffering from subfertility visiting the clinics participating in the study will undergo a basic fertility work-up including a semen-analysis, an assessment of tubal patency using CAT or HSG, and monitoring of the cycle to assess ovulation. After the work-up has been completed, a fertility diagnosis will be made and a prognosis for treatment independent pregnancy will be calculated (Hunault et al.,2004), followed by a management proposal for the individual couple.
Ovulatory and anovulatory women with a body mass index between 29 kg/m^2 and 40 kg/m^2 will be approached to participate in the present study. After informed consent, they will be randomly allocated to an intervention program to improve lifestyle with a duration of six months, or to “usual care”.

4.2 Inclusion criteria

We aim to include subfertile women with a BMI between 29 kg/m^2 and 40 kg/m^2. Women have to be between 18 and 38 years old.
Subfertility is defined as failure to conceive within 12 months of unprotected intercourse in couples(where the woman has an ovulatory cycle), as well as couples where the women suffers from chronic anovulation due to WHO I or II or PCO.

4.3 Exclusion criteria

Couples suffering from azoospermia, severe endometriosis AFS 3 and 4 (Revised American Fertilty Society classification), chronic anovulation due to WHO III, endocrinopathies (like: cushing syndrome, adrenal hyperplasia, hypothyroidism, diabetes mellitus type I) will not be eligible for the study. Women with preexistent hypertension or diabetes, or pregnancy

induced hypertension, preeclampsia, eclampsia or HELLP syndrome in a previous pregnancy are also excluded.
Incapacitated adults will be not asked to participate in the study.

Women eligible for the study will de referred to a research nurse. This person is dedicated to counselling eligible patients and will perform randomisation and collect follow-up data, but will not perform the experimental intervention. The lifestyle intervention programme will be done by trained intervention nurses. We will collaborate with the research nurses who are involved in the obstetric and urogynaecological consortium. In this consortium, more than 20 large randomised clinical trials are performed. Information on this infrastructure is obtainable from http://www.studies-obsgyn.nl/

4.4 Sample size calculation

SAMPLE SIZE: On the basis of the literature the cumulative live birth rate in a follow up of two years of subfertility treatment will be 45% for the "usual care" group (Dutch OFO-study, unpublished data). The lifestyle intervention group is estimated to have a life birth rate of 60% (Dutch OFO-study, unpublished data)

We expect an improvement of healthy singleton of more than 37 week gestation born after vaginal delivery from 45% to 60% in the intervention group. Drop out is expected to be 20% in the intervention arm and is included in the analysis of effectiveness.

To prove that, we need to include two groups of 272 women to be included (alpha 0.05, power 80%). To account for 5% lost to follow up 285 women will be included per group. In total 570 women will be included.

5. TREATMENT OF SUBJECTS

5.1 Investigational treatment

THE "USUAL CARE" ARM: In the "usual care" arm treatment will be started if the individual prognosis, based on the Hunault model (Hunault et al.2004) is less than 30% chance of conception within one year or when more than 3 years subfertility (guideline NVOG) justifies starting treatment. Even so, in case of chronic anovulation, ovulation induction will be started. Fertility treatment can either be IUI, IVF or ICSI whatever is indicated according to the Dutch guideline on IVF treatment (NVOG). In anovulatory patients, ovulation induction will be started using clomiphene, or gonadotrofines, which is applicable. When the Hunault model shows a prognosis of more than 30% pregnancy chance in the forthcoming year (and patients are less than three years subfertile), expectant management will be offered.

THE EXPERIMENTAL ARM: The overweight intervention is based on the lifestyle module which is developed in ZONMW project (50-50110-98-078) and will be adapted for the study

proposal in this specfic group of subfertile woman for a duration of six months. The software module developed, used and evaluated in this program will be adapted for this subfertile patient group. The use of the software program will reduce practice variation between fertility centres. The lifestyle intervention is based on the recommendations of Dutch CBO guideline "Diagnostiek en behandeling van obesitas bij volwassenen en kinderen" and consists of a structured lifestyle program targeted at:

1. Changing the dietary pattern.
2. Stimulating physical activity of moderate intensity.
3. Changing existing behaviour.
4. Self- monitoring.
5. Involvement of the partner.
6. Referral to psychologist when indicated.

ad 1. CHANGING DIETARY PATTERN

Women will be advised to adapt their dietary pattern and sustain a healthy diet with a caloric reduction of approximately 600kcal compared to their normal caloric intake (but not below 1200kcal/day). A realistic target weight will be set of 5-10% below their body weight. The diet should be based on the Dutch guideline "Gezonde voeding" in order to achieve a sustainable diet change.

ad 2. STIMULATING PHYSICAL ACTIVITY

Physical activity is necessary in order to sustain a modest weight loss and increase effect of dietary measures. Patients will use a stepcounter (PAM) to measure daily steps aiming for a daily goal of 10.000 steps. Two to three times a week sporting of at least 30 minutes of moderate intensity (60-85% of maximum hearth frequency) is advised. Sporting advice is individualized taking into account a woman's preference and possibilities (swimming, walking, stepping, cycling, cardio-fitness, etc.). Patients keep a diary to not the result of the stepcounter, and the sporting activities. These results are evaluated by the trained nurses.

ad 3. CHANGING EXISTING BEHAVIOUR

The motivation to change lifestyle is measured and evaluated during the program with the PACE score measuring stages of change (precontemplative stage, considering -preparatory stage, action stage and maintenance stage). This PACE score is part of the software module “GOAL”. Motivational counselling is individualized for these stages of change. Changing existing behaviour is achieved by motivational counselling directed at:

-Awareness of actual lifestyle leading to overweight or obesity.

-Counselling healthy lifestyle measures, the effect of healthy lifestyle in relation to subfertility and spontaneous and treatment dependent pregnancy chances as well as pregnancy complications and influence on perinatal outcome.

-Individualized goals are formulated and embedded in a "patient contract". "Patient contracting" is a tool to actually individualize the measures that the patient will take and to stick to the goals and targets that are agreed upon. During the intervention individual goals will be evaluated, feedback will be given and goals adapted when necessary. Continuous

evaluation and feedback will be maintained focussed on the target bodyweight and long term maintenance of lifestyle changes.

ad 4. SELFMONITORING
Self-monitoring is an essential tool to improve compliance during a lifestyle program. It will be implemented using a PAM "stepcounter" to measure daily physical activity and the “eetmeter” (www.voedingscentrum.nl). This webbased tool is used to give feedback on food intake, and caloric intake on a daily basis. Patients will be trained to use this device. A diary with sporting activities as well as caloric intake will be evaluated by the dedicated nurses.

ad 5. INVOLVEMENT OF THE PARTNER
Involvement of the partner and family of the women is important in order to guarantee social support and a "buddy system" during lifestyle changes.

ad 6. REFERRAL
When indicated, referral to psychologist or intervention available in the general health care system will used.

Patients undergoing the lifestyle program will be guided and supported by trained nurses or dieticians or nurse practitioners, who will be trained prior to the study. Women who miss two consecutive sessions and who are not willing to catch up in another group are excluded from the program.

The standardized lifestyle program consists of four sessions in the first three months, and than a booster of two sessions in the last three months. Telephone consultation or consultation by mail is scheduled in between these sessions four times. (see table 1)

FOLLOW-UP:
All participating couples (usual care arm and the experimental care arm) will complete several questionnaires, i.e. the SF-36: measuring satifaction, the SQUASH list: for physical activity, the FFQ: food frequency questionnaire for assessing nutrient intake): a structured questionnaire about food pattern (e.g. breakfast yes/no) and important food components (e.g. snacks, fruits and vegetables and meat), the Dutch Eating Behaviour Questionnaire (DEBQ): for assessment of restrained, emotional and external eating behaviour (van Strien 1986). Finally, several questions are completed i.t.o. history of body weight, e.g. the duration of overweight, weight fluctuations.
Questionnaires are webbased. In case a patient does not use the computer paper questionnaires will be used. Questionnaires will be completed at start, 12 weeks, 24 weeks and 52 weeks after randomisation. In addition each woman will receive a questionnaire for details on associated direct costs of professional care and on indirect costs like transportation, sporting activity and productivity loss. These questionnaires will be handed out in consultation 2, week 3 of the lifestyle module or at the start of fertility treatment for

women that were randomised for “usual care”. This will be repeated at the time points of the other questionnaires (12, 24 and 52 weeks after randomisation and at the end of follow-up).

Moreover, we will register reproductive outcome, fertility treatments as well as the course and outcome of subsequent pregnancies, including obstetrical interventions for a period up to 24 months after randomisation.

6. METHODS

6.1 Study parameters/endpoints

Background and Demographic Characteristics
To assess whether the treatment groups were balanced, the study populations will be compared for baseline measurements including female age, type of infertility (primary/secondary), duration of infertility, intoxications, body mass index, as well as sperm analysis according to WHO standards.

6.1.1 Main study parameter/endpoint

The primary endpoint will be a healthy singleton of more than 37 week gestation born after vaginal delivery. The initial analysis, will be done by intention to treat, so we will not make a difference for treatment independent pregnancies and pregnancies that occurred after treatment. Patients who will drop out of the study will by analysed according to their group of randomisation. In a per protocol analysis drop outs will be asked to provide information on the primary outcome, i.e. life birth within two years after randomisation. These drop outs will be analysed as non-compliant.

6.1.2 Secondary study parameters/endpoints

Secondary outcome parameters are:
1. pregnancy outcome and -complications: miscarriage, gestational diabetes, Pregnancy induced hypertension, preeclampsia, HELLP, prematurity<37 weeks, macrosomia> 4200 grams, induction of labour, prolonged duration of labour, operative delivery, and increased blood loss, and multiple pregnancies
2. percentage of women needing fertility treatment in both groups (OI, IUI, IVF, ICSI) and ongoing pregnancy rates in these treatments.
3. perinatal outcome: stillbirth, pregnancy duration, body weight and health of neonate, neonatal admission.
4. quality of life, pre-pregnancy body weight, weight gain during pregnancy, waist circumference, behaviour influencing weight, i.e. nutritional habits and exercise pattern, blood pressure, glucose/insulin ratio (HOMA), hormonal profile (androgens, adipokines and cytokines) and costs.

6.1.3 Other study parameters

ECONOMIC EVALUATION: The aim of the economic evaluation is to compare the costs and health effects of a lifestyle program versus “usual care”. The analysis will be a cost-effectiveness analysis with the proportion of healthy singleton of more than 37 week gestation born after vaginal delivery as the primary outcome.

6.2 Randomisation, blinding and treatment allocation

The randomisation is performed by computer at a central randomisation centre in the AMC. Randomisation will be stratified according to presence or absence of anovulation and center of treatment.
Women eligible for the study will de referred to a research nurse. This person is dedicated to counselling eligible patients and will perform randomisation and collect follow-up data, but will not perform the experimental intervention. We will collaborate with the research nurses who are involved in the obstetric and urogynaecological consortium. In this consortium, eight large randomised clinical trials are performed. Information on this infrastructure is obtainable from http://www.studies-obsgyn.nl/

6.3 Study procedures

TIME SCHEDULE LIFESTYLE PROGRAM
(see flow chart I: TIME SCHEDULE LIFESTYLE PROGRAM)

WEEK 1: BASELINE ASSESSMENT.
Consultation 1.
Counselling session 45-60 minutes.
Motivational counselling will include an extensive history of weight changes, knowledge and insight of weight related health problems.
Awareness, actual behaviour and lifestyle leading to overweight and history of body weight and slimming are discussed.
Counselling negative effect of overweight on frequencies of spontaneous and treatment dependent pregnancy chances and increase frequency of complications during pregnancy for mother and foetus.
The stages of change can be identified using the PACE score in the GOAL software.
DEBQ is evaluated to asses the type of eating behaviour.
Counselling and providing knowledge and advice about healthy lifestyle.
Evaluating diet questionnaire
Evaluating SQUASH score for physical activity
Healthy diet is advised aiming for a reduction of 600 Kcal/day (“Energy reduced diet”). Diet should be based on the Dutch guideline “Gezonde Voeding”. Food intake should not be reduced below 1200 Kcal/day in order to achieve a sustainable lifestyle change.
Handout of "eetmeter" en stepcounter and food and physical activity diary

Use of stepcounter is explained: 10.000 steps/day and two to three times a week sporting of at least 30 minutes of moderate intensity (60-85% of maximum hearth frequency).
Website www.voedingscentrum.nl/eetmeter is explained,
Eating and food diary is introduced.
Partner is involved in the counselling session
Referral to a dietician or psychologist is done in a standardized way, when appropriate.
Measuring waist circumference and weight.
A blood sample of 10 cc will be taken and serum stored.

WEEK 3: SETTING REALISTIC TARGETS AND GOALS.
Consultation 2.
Counselling session of 45-60 minutes.
Focus on agreeing on achievable targets on an individual basis regarding physical activity, diet and body weight.
Food -and physical activity diary including results of "eetmeter" and stepcounter are evaluated. Diet: reduction of 600 Kcal/day (but not below 1200 Kcal/day)is introduced.
Physical activity aiming at 10.000 steps a day.
Patient contract describing realistic goals c.q. agreements on an individual basis regarding: weight loss (…. Kg/ …. Weeks) and physical activity (…. Steps/ …. day), and sporting activity (two to three times a week),
Plan activities to reach goals.
Address barriers and promote facilitating factors to achieve sustainable lifestyle changes.
A questionnaire for details on indirect costs like transportation, sporting activity and productivity loss will be handed out.
Measuring waist circumference and weight.

WEEK 5. CONSULTATIONS VIA TELEPHONE OR MAIL (SEE BELOW)

WEEK 7: EVALUATION AND FEEDBACK
Consultation 3.
Counselling session of 30 minutes.
Regarding: motivation weight loss and physical activity.
Evaluating results regarding: bodyweight, food intake diary, "eetmeter", physical activity diary, stepcounter
Feedback on results.
Individualized advice for improvement when necessary and adjust agreements when necessary to reach realistic targets.
Discuss relapse.
Evaluating the barriers to follow the program when necessary.
Referral to a dietician or psychologist is done in a standardized way, when appropriate.
Measuring waist circumference and weight.

WEEK 8. CONSULTATIONS VIA TELEPHONE OR MAIL (SEE BELOW)

WEEK 12: EVALUATION AND FEEDBACK
Consultation 4.
Counselling session of 15-30 minutes.
Regarding: motivation weight loss and physical activity.
Evaluating results regarding: bodyweight, food intake diary, "eetmeter", physical activity diary, stepcounter.
Feedback on results.
Individualized advice for improvement when necessary and adjust agreements when necessary to reach realistic targets.
Discuss relapse.
Evaluating the barriers to follow the program when necessary.
Referral to a dietician or psychologist is done in a standardized way, when appropriate
Measuring waist circumference and weight.
A blood sample of 10 cc will be taken and serum stored

WEEK 15. CONSULTATIONS VIA TELEPHONE OR MAIL (SEE BELOW)

WEEK 18 EVALUATION AND FEEDBACK
Consultation 5.
Counselling session of 15-30 minutes.
Regarding: motivation weight loss and physical activity
Evaluating results regarding: weight, food diary, eetmeter, physical activity, stepcounter, -
Feedback on results.
Individualized advice for improvement when necessary and adjust agreements when necessary to reach realistic targets.
Discuss relapse.
Evaluating the barriers to follow the program when necessary.
Referral to a dietician or psychologist is done in a standardized way, when appropriate.
Measuring waist circumference and weight.

WEEK 21. CONSULTATIONS VIA TELEPHONE OR MAIL (SEE BELOW)

WEEK 24 EVALUATION OF TARGETS
Consultation 6.
Counselling session of 15-30 minutes.
Evaluation of targets:
Weight reduction
Physical activity
Behaviour change
Measuring waist circumference and weight.
A blood sample of 10 cc will be taken and serum stored.

CONSULTATIONS VIA TELEPHONE OR MAIL WEEK 5, 8, 15, AND 21.

In between the consultations in the outpatient fertility clinic, consultation via telephone or mail are scheduled at week 5, week, 9, week 15, and week 21 with a duration of 15 minutes. In these consultations evaluation regarding: motivation of weight loss and physical activity are discussed

Evaluating results regarding: weight, food diary, eetmeter, physical activity, stepcounter.

Feedback on results.

Individualized advise for improvement when necessary and adjust agreements when necessary to reach realistic targets.

Discuss relapse.

Evaluating the barriers to follow the program when necessary.

Questionnaires for all included women: SF36, FFQ and Squash-list will be evaluated at the start and week 12 and week 24 and 52 weeks after randomisation.

Start of fertility treatment

When in spontaneous ovulatory patients in the lifestyle intervention arm:

- have finished their six month lifestyle program or,
- when they meet their target weight reduction of 5-10% or,
- when their weight decreases < BMI 29 kg/m²,

fertility treatment will be started if the individual prognosis, based on the Hunault model (Hunault et al.2004) if less than 30% chance of conception within one year or when more than 3 years subfertility (guideline NVOG) justifies starting treatment (either IUI, IVF or ICSI (according to national guidelines NVOG).

In case of chronic anovulation, ovulation induction will be started when patients in the lifestyle intervention arm:

- have finished their six month lifestyle program or,
- when they meet their target weight reduction of 5-10% or,
- when their weight decreases < BMI 29 kg/m².

Table 1: time schedule lifestyle intervention

Week	Consultation Outpatient/ Telephone or mail	Counseling subject lifestyle program	Duration minutes
1	Outpatient 1	Baseline assessment and explanation lifestyle program; handout of “eetmeter”, stepcounter and diary . Blood sample (10 cc)	45-60
3	Outpatient 2	Setting targets and planning goals; evaluating baseline measures , hand out of cost-questionaires	45-60
5	Telephone 1	Evaluating targets	15

7	Outpatient 3	Evaluating targets	30
9	Telephone or mail 2	Evaluating targets	15
12	Outpatient 4	Evaluating targets, handout of questionnaires<br>Blood sample (10 cc)	30
15	Telephone or mail 3	Evaluating targets	15
18	Outpatient 5	Evaluating targets	30
21	Telephone or mail 4	Evaluating targets	15
24	Outpatient 6	Assessing targets and evaluation lifestyle program and handout of questionnaire	30
>24	Outpatient	Start infertility treatment when applicable<br>Blood sample (10 cc)	
52		questionnaires	

6.4 Withdrawal of individual subjects

Subjects can leave the study at any time for any reason if they wish to do so without any consequences. Patients who decide to withdraw from the study will be asked by the responsible investigator to provide information regarding the primary endpoint of the study 24 months after randomisation. Patients who drop out of the study will be treated according to the local protocols for infertility patients.
The investigator can decide to withdraw a subject from the study for medical reasons. Women who miss two consecutive counselling sessions and who are not willing to catch up are excluded from the program.

7. SAFETY REPORTING

7.1 Section 10 WMO event

In accordance to section 10, subsection 1, of the WMO, the investigator will inform the subjects and the reviewing accredited METC if anything occurs, on the basis of which it appears that the disadvantages of participation may be significantly greater than was foreseen in the research proposal. The study will be suspended pending further review by the accredited METC, except insofar as suspension would jeopardise the subjects' health. The investigator will take care that all subjects are kept informed.

7.2 Adverse and serious adverse events

Adverse and serious adverse events as a direct result of the lifestyle intervention are not anticipated in this study. However, patients with overweight or obesity have additional risks during pregnancy compared to normal weight women. As a result of both the intervention arm and of the “care as usual” arm, pregnancies may occur in patients with increased risks. The risks involve hypertension (OR: 2.2), preeclampsia (OR: 1.8-2.7), pregnancy-induced diabetes (OR: 2.2-3.4), caesarian section (OR: 1.3-2.4) (see tables of evidence in appendix). These risks are not influenced by fertility treatment. Lifestyle intervention aims to reduce the involved pregnancy risks. Participants in the study have been informed about these risks.
An independent Safety Monitoring Board (SMB) will be installed to review major complications in both treatment groups. This board will evaluate complications after every 150 included patients and six months and 12 months after the end of inclusion of the study (6 times during the study). The SMB will differentiate between major en minor complications, blinded for treatment arm. Every case of preeclampsia, eclampsia, HELLP syndrome, will be reported to the SMB. The SMB findings will be reported to the Ethics Committee of the UMCG

Adverse events are defined as any undesirable experience occurring to a subject during a clinical trial, whether or not considered related to the investigation. All adverse events reported spontaneously by the subject or observed by the investigator or his staff will be recorded.
A serious adverse event is any untoward medical occurrence or effect that at any dose results in death;

- is life threatening (at the time of the event);
- requires hospitalisation or prolongation of existing inpatients’ hospitalisation;
- results in persistent or significant disability or incapacity;
- is a congenital anomaly or birth defect;
- is a new event of the trial likely to affect the safety of the subjects, such as an unexpected outcome of an adverse reaction, lack of efficacy of an IMP used for the treatment of a life threatening disease, major safety finding from a newly completed animal study, etc.

All SAEs will be reported to the accredited METC that approved the protocol, according to the requirements of that METC.

7.3 Follow-up of adverse events

All adverse events will be followed until they have abated, or until a stable situation has been reached. Depending on the event, follow up may require additional tests or medical procedures as indicated, and/or referral to the general physician or a medical specialist.

8. STATISTICAL ANALYSIS

8.1 Descriptive statistics

To asses whether the groups were balanced the study population will be compared for baseline measurements including female age, type of subfertility (primary or secondary), duration of subfertility, intoxications, smoking, BMI, as well as sperm analysis according to WHO standards, anovulation, and subfertility treatment. Confounding factors, such as smoking will be addressed in the analysis.

8.2 Univariate analysis and multivariate analysis

The primary analysis will be by intention to treat.
The primary outcome will be analysed by comparing the pregnancy rates in both groups using a chi-square test. Relative risks and 95% confidence intervals will be calculated. Delivery rates over time will be compared using life table methods with correction for confounders (Cox-regression analysis). Exploratory subgroup analyses will be performed for spontaneous and treatment-induced pregnancies and for women with a BMI below 35 versus above 35, as well as for anovulatory versus ovulatory women and for women with a waist-hip ratio of above 0.8 versus below or equal to 0.8.
Additional analysis concerning the influence of HOMA, androgens, adipokines and cytokines on pregnancy chances and recovery of ovulations in anovulatory patients will be conducted.
Incidence of complications of treatment and during pregnancy will be compared in both groups using relative risks and 95% confidence intervals.
Quality of life will be analysed using repeated measures analysis of variance

A per protocol analysis will also be performed, in which drop out patients will be identified as non-compliant. Information on the primary outcome, i.e.lifebirth within two years after randomisation will be used whenever provided. Patients who drop out of the lifestyle intervention arm will be analysed in the "usual care" arm in this per protocol analysis.

Women who drop out of the study will be asked to provide the reason for dropping out. This reason will be recorded and patients will be asked to provide information regarding the primary outcome, i.e. pregnancy within two years after randomisation. In addition, patients will be offered the possibility to continue to complete cost questionnaires for the remainder of the study duration. For patients dropping out of the lifestyle intervention, further course will be determined by the responsible physician.

8.3 Economic evaluation

The aim of the economic evaluation is to compare the costs and health effects of a lifestyle program versus "usual care". The analysis will be a cost-effectiveness analysis with the proportion of healthy singleton of more than 37 week gestation born after vaginal delivery as the primary outcome.
The economic evaluation will be performed from a societal perspective. Direct medical and non-medical costs (intervention costs, time and travel costs) as well as indirect non-medical costs (productivity losses) will be taken into account.

We will build on the work of the ZonMW funded "Paraplu" study, in which cost data data of six cost-effectiveness studies were integrated. Dr H. Groen played an important role in this process.

The time horizon will be from randomisation to the end of follow-up. Resource use will be recorded as individual patient data in the CRF. In addition each woman will receive a questionnaire for details on associated direct costs of professional care and on indirect costs like transportation, sporting activity and productivity loss. These questionnaires will be handed out in consultation 2, week 3 of the lifestyle module or at the start of fertility treatment for women that were randomised for "usual care". This will be repeated at the time points of the other questionnaires (12, 24 and 52 weeks after randomisation and at the end of follow-up).
Resource use will be valued according to Dutch guidelines. Intervention costs will determined based on actual resource use obtained from the centers. Costs will reflect the following resources: staff, materials, equipment, housing, overhead. Productivity loss will be valued by the friction cost method according to Dutch guidelines (Oostenbrink et al. 2004). Outcome of pregnancy will be recorded and valued using tariffs or data from the literature (Lukassen et al, 2004).

Detailed information on maternal complication will be obtained from obstetricians treating the woman concerned using patient medical files. Six weeks after the expected day of delivery all women will be contacted by telephone to ask information on the delivery and on the health of the child. If the child has been hospitalised the paediatrician treating the child will be contacted for further information.

Scenario analysis will be performed to model cost-effectiveness beyond the time horizon of the study. For longer term analyses, costs and effects will be discounted at commonly accepted rates. Sensitivity analysis will be performed on costs, pregnancy rates of the two groups and the valuation of different outcomes (no child, handicapped child, twin, healthy child, obstetric complications). Uncertainty surrounding cost-effectiveness estimates will be explored by bootstrapping. Cost will be presented in euros.

9. ETHICAL CONSIDERATIONS

9.1 Regulation statement

The study will be conducted according to the principles of the Declaration of Helsinki (Adopted by the 18th WMA General Assembly, Helsinki, Finland, June 1964, and amended by the:
29th WMA General Assembly, Tokyo, Japan, October 1975
35th WMA General Assembly, Venice, Italy, October 1983
41st WMA General Assembly, Hong Kong, September 1989
48th WMA General Assembly, Somerset West, Republic of South Africa, October 1996
and the 52nd WMA General Assembly, Edinburgh, Scotland, October 2000
Note of Clarification on Paragraph 29 added by the WMA General Assembly, Washington 2002
Note of Clarification on Paragraph 30 added by the WMA General Assembly, Tokyo 2004
and in accordance with the Medical Research Involving Human Subjects Act (WMO)

9.2 Recruitment and consent

Women eligible for the study will de referred to a research nurse by their treating doctors. This person is dedicated to counselling eligible patients, randomisation and follow-up for data, but not to the experimental intervention. We will collaborate with the research nurses who are involved in the obstetric and urogynaecological consortium. In this consortium, eight large randomised clinical trials are performed. Information on this infrastructure is obtainable from http://www.studies-obsgyn.nl/
The research nurse must explain to each subject the nature of this study, its purpose, procedures, expected duration and the potential risks and benefits involved in study participation along with any discomfort it may entail. Each subject must be informed that participation in the study is voluntary and that withdrawal of consent will not affect his/her right to the most appropriate medical treatment or affect the doctor relationship.
This informed consent will be given by means of a standard written statement. It will be written so as to be easily understood by the subject. The subject will be given the time to read and understand the statement herself before signing her consent and dating the document. The subject will receive a copy of the written statement once signed.
The informed consent form must be considered as a part of the protocol with which it is to be submitted by the investigator to the IRB/Ethics committee.
Patients will be given a maximum of one week to consider their decision to participate in the study.

9.3 Objection by minors or incapacitated subjects (if applicable)

Incapacitated adults will be not asked to participating in the study. Minors are excluded as well.

9.4 Benefits and risks assessment, group relatedness

The ethical issues pertaining to this project will be discussed by considering the four ethical principles as discussed by Gillon. (37)

Autonomy: After the intake consultation and counseling session by the research nurse, the patient is given the choice to participate in the study. This process of informed consent respects her autonomy.

Beneficence: The advantages of a weight loss and exercise program in overweight or obese subfertile women (in order to improving chances of conception and eventually taking a baby home) have clearly been shown. Even if the patient does not conceive during the 6 months period, the health benefits of weight loss on the long-term are clearly in the interest of the patient.

Non-maleficence: In some reproductive medicine units, overweight or obese subfertile women are counselled and advised to loose weight before treatment is started. By starting this project, fertility treatment in the intervention arm will be delayed for a maximum of 6 months. There is no evidence that this short delay will influence the eventual pregnancy chances. The structured weight loss and exercise program will probably lead to higher spontaneous and therapy induced ongoing pregnancy chances. Patients with overweight or obesity have additional risks during pregnancy. The risks involve hypertension (OR 2.2), preeclampsia (OR 1.8-2.7) pregnancy diabetes (OR 2.2-3.4), ceasarean section (OR 1.3-2.4) compared to normal weight women (see appendix table of evidence). These risks are not additionally influenced by fertility treatment. Lifestyle intervention aims to reduce the involved pregnancy risks and is part of the analysis of this study. The lifestyle intervention perse does not involve additional risk to the patients, but reduces the risks of longterm health complications.

Before giving informed consent all eligible women are informed about the risks mentioned before (in writing in the patients' brochure). Women who are considered to be at the highest risk (i.e. those who have pre-existent hypertension or diabetes, or pregnancy induced hypertension, (pre)eclampsia or HELLP syndrome in a previous pregnancy) are excluded from participation. During the study adverse events will be monitored by a SMB.

Justice: Introducing the study will not infringe on other's rights (rights based justice). The study will mainly be financed through ZonMW and therefore no conflict distribution of scarce funds (distributive justice). Inclusion in the study respects all morally acceptable laws (legal justice).

Compensation for injury

Ingevolge artikel 7 van de Wet op medisch wetenschappelijk onderzoek met mensen (Stlb. 1998, 161) is voor de deelnemende proefpersonen een verzekering afgesloten die de door het onderzoek veroorzaakte schade door dood of letsel van de deelnemende proefpersonen dekt. Deze verzekering voldoet aan de bepalingen van het Besluit Verplichte verzekering bij medisch-wetenschappelijk onderzoek met mensen (Stlb. 2003, 266).
Aan het onderzoek deelnemende proefpersonen zullen schriftelijk worden ingelicht over deze verzekering.
Elke aan het onderzoek participerende instelling draagt zorg voor de verzekering van de in de eigen instelling te includeren proefpersonen.
Elk deelnemend centrum heeft ook een aansprakelijkheidsverzekering als bedoeld in art. 7 WMO.

10. ADMINISTRATIVE ASPECTS AND PUBLICATION

10.1 Handling and storage of data and documents

Data will be handled confidentially and anonymously. To be able to trace data to an individual subject, a subject identification code list will be used. Participating subjects will be registered by a 5-digit number that will be used to link the data to the subject. This personal code will be on all forms retrieved from participants. The key to the code is safeguarded by the investigator. The handling of personal data will comply with the Dutch Personal Data Protection Act (in Dutch: De Wet Bescherming Persoonsgegevens, Wbp).
Serum will be stored for 5 years at minus 80 degrees anonymously and coded as mentioned above.

10.2 Amendments

Amendments are changes made to the research after a favourable opinion by the accredited METC has been given. All amendments will be notified to the METC that gave a favourable opinion.

10.3 Annual progress report

The sponsor/investigator will submit a summary of the progress of the trial to the accredited METC once a year. Information will be provided on the date of inclusion of the first subject, numbers of subjects included and numbers of subjects that have completed the trial, serious adverse events/ serious adverse reactions, other problems, and amendments.

10.4 End of study report

The investigator will notify the accredited METC of the end of the study within a period of 8 weeks. The end of the study is defined as the last patient's last visit.
In case the study is ended prematurely, the investigator will notify the accredited METC, including the reasons for the premature termination.

Within one year after the end of the study, the investigator/sponsor will submit a final study report with the results of the study, including any publications/abstracts of the study, to the accredited METC.

11. REFERENCES

Al-Azemi M, Omu FE, Omu AE.The effect of obesity on the outcome of infertility management in women with polycystic ovary syndrome.Arch Gynecol Obstet. 2004 Dec;270(4):205-10.

Amer SA, Li TC, Ledger WL.Ovulation induction using laparoscopic ovarian drilling in women with polycystic ovarian syndrome: predictors of success.Hum Reprod. 2004 Aug;19(8):1719-24.

Avenell A, Broom J, Brown TJ, Poobalan A, Aucott L, Stearns SC, Smith WC, Jung RT, Campbell MK, Grant AM. Systematic review of the long-term effects and economic consequences of treatments for obesity and implications for health improvement.
Health Technol Assess. 2004 May;8(21):iii-iv, 1-182. The British Dietetic Association Ltd. J Hum Nutr Diet 2004; 17: 317-35.

Balen AH, Anderson RA. Impact of Obesity on female reproductive health: British Fertility Society, Policy and Practice Guidelines. Hum Fertil (Camb). 2007 Dec;10(4):195-206.

Balen AH, Dresner M, Scott EM, Drife JO. Should obese women with polycystic ovary syndrome receive treatment for infertility? BMJ 2006;332:434-5.

Bemelmans WJE, et al. Interventies ter preventie van overgewicht in de wijk, op school, op het werk en in de zorg. Een verkennende studie naar de effecten. RIVM report 260301005, Bilthoven, 2004.

Bemelmans WJE, et al. Leefstijladvisering door praktijkondersteuner bij mensen met overgewicht : opzet , procesevaluatie en eerste resultaten van de Groningse Overweight and Lifestyle (GOAL) study. RIVM Rapport

Bemelmans W, van Baal P, Wendel-Vos W, Schuit J, Feskens E, Ament A, Hoogenveen R.

The costs, effects and cost-effectiveness of counteracting overweight on a population level. A scientific base for policy targets for the Dutch national plan for action. Prev Med. 2008 Feb;46(2):127-32. Epub 2007 Aug 3.

Cnattingius S, Bergström R, Lipworth L, Kramer MS. Prepregnancy weight and the risk of adverse pregnancy outcomes. N Engl J Med 1998;338(3):147-52.

Bogers RP, Vijgen SMC, Bemelmans WJE.Costs of lifestyle interventions within health care and the amount of weight loss achieved. RIVM rapport 260701002/2006, Bilthoven 2006.

Bolúmar F, Olsen J, Rebagliato M, Sáez-Lloret I, Bisanti L. Body mass index and delayed conception: a European Multicenter Study on Infertility and Subfecundity. Am J Epidemiol. 2000 Jun 1;151(11):1072-9.

Bussen S, Sütterlin M, Steck T.Endocrine abnormalities during the follicular phase in women with recurrent spontaneous abortion. Hum Reprod 1999;14:18-20.

CBO guidline Diagnostiek en behandeling van obesitas bij volwassenen en kinderen. www.cbo.nl 2007

Clark AM, Ledger W, Galletly C, Tomlinson L, Blaney F, Wang X l. Weight loss results in significant improvement in pregnancy and ovulation rates in anovulatory obese women. Hum Reprod 1995;10:2705-12.

Clark AM Thornley B, Tomlinson L, Galletley C, Norman RJ . Weight loss in obese infertile women results in improvement in reproductive outcome for all forms of fertility treatment. Hum Reprod 1998;13:1502-05.

Cnattingius S, Taube Al. Stillbirth and rate of fetal death in 76,761 postterm pregnancies in Sweden 1991; a register study. Acta Obstet Gynecol Scand 1998;77:582

Clinical Guidelines on the Identification, Evaluation, and Treatment of Overweight and Obesity in Adults--The Evidence Report. National Institutes of Health. Obes Res 1998;6(S)2:51-209.

Custers IM, Steures P, van der Steeg JW, van Dessel TJ, Bernardus RE, Bourdrez P, Koks CA, Riedijk WJ, Burggraaff JM, van der Veen F, Mol BW. External validation of a prediction model for an ongoing pregnancy after intrauterine insemination. Fertil Steril. 2007 Aug;88(2):425-31..

Dechaud H, Anahory T, Reyftmann L, Loup V, Hamamah S, Hedon B. Obesity does not adversely affect results in patients who are undergoing in vitro fertilization and embryo transfer. Eur J Obstet Gynecol Reprod Biol. 2006 Jul;127(1):88-93.

Crosignani PG, Colombo M, Vegetti W, Somigliana E, Gessati A, Ragni G. Overweight and obese anovulatory patients with polycystic ovaries: parallel improvements in anthropometric indices, ovarian physiology and fertility rate induced by diet. Hum Reprod 2003 Sep;18(9):1928-32.

Dixon JB, Dixon ME, and O'Brien PE. Birth outcomes in obese women after laparoscopic adjustable gastric banding. Obstet Gynecol 2005;106:965-72.

Dodson WC, Kunselman AR, Legro RS. Association of obesity with treatment outcomes in ovulatory infertile women undergoing superovulation and intrauterine insemination. Fertil Steril. 2006 Sep;86(3):642-6..

Dokras A, Jagasia DH, Maifeld M, Sinkey CA, VanVoorhis BJ, Haynes WG.Obesity and insulin resistance but not hyperandrogenism mediates vascular dysfunction in women with polycystic ovary syndrome. Fertil Steril. 2006 Dec;86(6):1702-9.

Dunaif A. Insulin resistance and the polycystic ovary syndrome: Mechanism and implications for pathogenesis. Endocr.Rev. 1997;18:774-800.

Edwards LE, Hellerstedt WL, Alton IR, Story M, Himes JH. Pregnancy complications and birth outcomes in obese and normal-weight women: effects of gestational weight change. Obstet Gynecol. 1996 Mar;87(3):389-94.

Fedorcsák P, Storeng R, Dale PO, Tanbo T, Abyholm T. Obesity is a risk factor for early pregnancy loss after IVF or ICSI. Acta Obstet Gynecol Scand 2000;79:43-8.

Fedorcsák P, Dale PO, Storeng R, Ertzeid G, Bjercke S, Oldereid N, Omland AK, Abyholm T, Tanbo T.Impact of overweight and underweight on assisted reproduction treatment. Hum Reprod 2004;19:2523-8.

Franz MJ, VanWormer JJ, Crain L, et al. Weight loss outcomes: a systematic review and meta-analysis of weight loss clinical trials with a minimum 1-year follow-up. J Am Diet Assoc 2007; 107:1755-1767.

Garbaciak JA Jr, Richter M, Miller S, Barton JJ. Maternal weight and pregnancy complications. Am J Obstet Gynecol 1985;152(2):238-45.

Glazer NL, Hendrickson AF, Schellenbaum GD, and Mueller BA. Weight change and the risk of gestational diabetes in obese women. Epidemiology 2004;15:733-7.

Gillon, R. (1994). Medical ethics: four principles plus attention to scope. BMJ. 309, 184-188

Guzick DS, Wing R, Smith D, Berga SL, Winters SJ. Endocrine consequences of weight loss in obese, hyperandrogenic, anovulatory women. Fertil Steril 1994 Apr;61(4):598-604.

Herman WH, Hoerger TJ, Brandle M, et al. The cost-effectiveness of lifestyle modification or metformin in preventing type 2 diabetes in adults with impaired glucose tolerance. Ann Intern Med 2005; 142(5):323-32.

Hoeger KM, et al. A randomized, 48-week, placebo ontrolled trial of intensive lifestyle modification and/or metformin therapy in overweight women with polycystic ovary syndrome: a pilot study. Fertil Steril 2004;82:421-9.

Hollmann M, Runnebaum B, Gerhard I. Effects of weight loss on the hormonal profile in obese, infertile women. Hum Reprod 1996 Sep;11(9):1884-91.

Huber-Buchholz MM, Carey DG, Norman RJ. Restoration of reproductive potential by lifestyle modification in obese polycystic ovary syndrome: role of insulin sensitivity and luteinizing hormone. J Clin Endocrinol Metab 1999;84:1470-4.

Hunault CC, Laven JS, van Rooij IA, Eijkemans MJ, te Velde ER, Habbema JD. Prospective validation of two models predicting pregnancy leading to live birth among untreated subfertile couples.Hum Reprod. 2005 Jun;20(6):1636-41.

Knowler WC et al. Reduction in the incidence of type 2 diabetes with lifestyle intervention or metformin. N Engl J Med 2002;346:393-403.

Imani B, Eijkemans MJ, te Velde ER, Habbema JD, Fauser BC. A nomogram to predict the probability of live birth after clomiphene citrate induction of ovulation in normogonadotropic oligoamenorrheic infertility. Fertil Steril. 2002 Jan;77(1):91-7.

Kiddy DS, Hamilton-Fairley D, Bush A, Short F, Anyaoku V, Reed MJ, et al. Improvement in endocrine and ovarian function during dietary treatment of obese women with polycystic ovary syndrome. Clin Endocrinol (Oxf) 1992 Jan;36(1):105-11.

Knowler WC, Barrett-Connor E, Fowler SE, Hamman RF, Lachin JM, Walker EA, Nathan DM; Diabetes Prevention Program Research Group.Reduction in the incidence of type 2 diabetes with lifestyle intervention or metformin. N Engl J Med. 2002 Feb 7;346(6):393-403.

Kristensen J, Vestergaard M, Wisborg K, Kesmodel U, Secher NJ. Pre-pregnancy weight and the risk of stillbirth and neonatal death. Pregnancy weight and the risk of stillbirth and neonatal death. BJOG 2005;112:403-8.

Lashen H, Lashen H, Ledger W, Bernal AL, Barlow D.Extremes of body mass do not adversely affect the outcome of superovulation and in-vitro fertilization.

Hum Reprod. 1999 Mar;14(3):712-5

Linne Y . 2004 Effects of obesity on women 's reproduction and complications during pregnancy. Obesity reviews 2004;5:137-43.

Lintsen AM, Pasker-de Jong PC, de Boer EJ, Burger CW, Jansen CA, Braat DD. Effects of subfertility cause, smoking and body weight on the success rate of IVF. Hum Reprod 2005;20:1867-75.

Lukassen HG, Schönbeck Y, Adang EM, Braat DD, Zielhuis GA, Kremer JA.
Cost analysis of singleton versus twin pregnancies after in vitro fertilization.
Fertil Steril. 2004 May;81(5):1240-6.

Maheshwari A, Stofberg L, Bhattacharya S. Effect of overweight and obesity on assisted reproductive technology –a systematic review. Human Reproduction Update update Hum Reprod Update. 2007 Sep-Oct;13(5):433-44.

McTigue KM, Harris R, Hemphill B, Lux L, Sutton S, Bunton AJ, Lohr KN.Screening and interventions for obesity in adults: summary of the evidence for the U.S. Preventive Services Task Force.Ann Intern Med. 2003 Dec 2;139(11):933-49

Moran LJ, Noakes M, Clifton PM, Tomlinson L, Galletly C, Norman RJ. Dietary composition in restoring reproductive and metabolic physiology in overweight women with polycystic ovary syndrome. J Clin Endocrinol Metab 2003 Feb;88(2):812-9.

Mulders AG, Eijkemans MJ, Imani B, Fauser BC.Prediction of chances for success or complications in gonadotrophin ovulation induction in normogonadotrophic anovulatory infertility. Reprod Biomed Online. 2003 Sep;7(2):170-8.

Mulders AG, Laven JS, Eijkemans MJ, Hughes EG, Fauser BC.Patient predictors for outcome of gonadotrophin ovulation induction in women with normogonadotrophic anovulatory infertility: a meta-analysis.Hum Reprod Update. 2003 Sep-Oct;9(5):429-49.

National Institute for Clinical Excellence. Fertility assessment and treatment for people withfertility problems. A clinical guideline. London: RCOG Press, 2004

Nichols JE, Crane MM, Higdon HL, Miller PB, Boone WR. Extremes of body mass index reduce in vitro fertilization pregnancy rates.Fertil Steril. 2003 Mar;79(3):645-7.

Norman RJ, Clark AM. Obesity and reproductive disorders, a review. Reprod Fertil Dev 1998;10:55-63.

Norman RJ, Noakes M, Wu R, Davies MJ, Moran L, Wang JX. Improving reproductive performance in overweight/obese women with effective weight management. Hum Reprod Update 2004;10:267-80.

Oers van JAM. Gezondheid op koers? Volksgezondheid Toekomst Verkenning 2002. Rijksinstituut voor Vokksgezondheid en Milieu, rapport nr. 270551001, Bilthoven 2002.

Oostenbrink JB, Koopmanschap MA, Rutten FF. Standardisation of costs: the Dutch Manual for Costing in economic evaluations. Pharmacoeconomics. 2002;20(7):443-54.

Oostenbrink JB Manual for costing : methods and standard costs for economic evaluation in helath care (In Dutch). Amstelveen, The Netherlands: Health Insurance Council ; Update version 2004.

Palomba S, Giallauria F, Falbo A, Russo T, Oppedisano R, Tolino A, et al. Structured exercise training programme versus hypocaloric hyperproteic diet in obese polycystic ovary syndrome patients with anovulatory infertility: a 24-week pilot study. Hum Reprod 2007 Dec 23.

Pasquali R, Pasquali R, Antenucci D, Casimirri F, Venturoli S, Paradisi R, Fabbri R, Balestra V, Melchionda N, Barbara L.Clinical and hormonal characteristics of obese amenorrheic hyperandrogenic women before and after weight loss.J Clin Endocrinol Metab. 1989 Jan;68(1):173-9.

Powell K, Pratt M.. Physical activity and health. BMJ 1996;313:126-7.

Ramlau-Hansen CH Thulstrup AM, Nohr EA, Bonde JP, Sorensen TIA, Olsen J. Subfecundity in overweight and obese couples. Human Reprod. 2007;22:1634-7

Sebire NJ, Jolly M, Harris JP, Wadsworth J, Joffe M, Beard RW, Regan L, Robinson S.Maternal obesity and pregnancy outcome: a study of 287,213 pregnancies in London. Int J Obes Relat Metab Disord. 2001 Aug;25(8):1175

Stamets K, Taylor DS, Kunselman A, Demers LM, Pelkman CL, Legro RS. A randomized trial of the effects of two types of short-term hypocaloric diets on weight loss in women with polycystic ovary syndrome. Fertil Steril 2004;81:630-7.

Tang T, Glanville J, Hayden CJ, White D, Barth JH, Balen AH. Combined lifestyle modification and metformin in obese patients with polycystic ovary syndrome. A randomized placebo-controlled, doube-blind multicentre study. Human Reprod 2006;21:80-9.

Usha Kiran TS, Hemmadi S, Bethel J, Evans J. Outcome of pregnancy in a woman with an increased body mass index.. BJOG 2005;112:768-72

Van der Steeg, Steures P, Eijkmans MJC, Habbema JDH, Hompes PGA, Broekmans FJ, van Dessel HJHM, Bossuyt PMM, van der Veen F, Mol BWJ. Pregnancy prediction is predictable: a large scale prospective external validation of prediction of spontaneous pregnancy in subfertile couples. Human Reprod. 2007;22;536-542.

van der Steeg JW JW, Steures P, Eijkemans MJ, Habbema JD, Hompes PG, Burggraaff JM, Oosterhuis GJ, Bossuyt PM, van der Veen F, Mol BW. Obesity affects spontaneous pregnancy chances in subfertile, ovulatory women.Hum Reprod. 2008 Feb;23(2):324-8.

Van Strien T, Rookus MA, Bergers GP, Frijters JE, Defares PB.Life events, emotional eating and change in body mass index. International Journal of Eating Disorders,5, 747-755. Life events, emotional eating and change in body mass index.Int J Obes. 1986;10(1):29-35

Vijgen SMC, Hoogendoorn M, Baan CA, de Wit GA, Limburg W, Feenstra TL. Cost effectiveness of preventive interventions in Type 2 Diabetes Mellitus. A systematic literature review. Pharmacoeconomics 2006; 24(5): 425-441.

Waller DK, Mills JL, Simpson JL, Cunningham GC, Conley MR, Lassman MR, and Rhoads GG. Are obese women at higher risk for producing malformed offspring? Am J Obstet Gynecol 1994;170:541-548.

Wang JX, Davis MJ, Norman RJ . Body mass and probability of pregnancy during assisted reproduction treatment. Brit Med J 2000; 321:1320-1.

Wang JX, Davis MJ, Norman RJ. Polycystic ovarian syndrome and the risk of spontaneous abortion following assisted reproductive technology treatment. Hum Reprod 2001;16:2606-9.

Wang JX, et al. Obesity increases the risk of spontaneous abortion during infertility treatment. Obes Res 2002;10:551-4.

Weiss JL, Malone FD, Emig D, Ball RH, Nyberg DA, Comstock CH, Saade G, Eddleman K, Carter SM, Craigo SD, Carr SR, D'Alton ME; FASTER Research Consortium
Obesity, obstetric complications and cesarean delivery rate--a population-based screening study. Am J Obstet Gynecol. 2004 Apr;190(4):1091-7.

Werler MM, Louik C, Shapiro S, and Mitchell AA. Prepregnant weight in relation to risk of neural tube defects. JAMA 1996,275:1089-92.

Wittemer C, Ohl J, Bailly M, Bettahar-Lebugle K, Nisand I.Does body mass index of infertile women have an impact on IVF procedure and outcome?J Assist Reprod Genet. 2000 Nov;17(10):547-52

WHO document ‘Ministerial conference on counteracting obesity’. WW.euro.who.int/document/E90143.pdf

Zelissen PM, Mathus-Vliegen EM. Treatment of overweight and obesity in adults: proposal for a guideline]Ned Tijdschr Geneeskd. 2004;16;148(42):2060-6 .

Summary of studies assessing outcome after lifestyle intervention

**Anovulatory patients**							
**article**	**subjects**	**Intervention kCal/day**	**duration in weeks**	**inclusion BMI**	**ovulation**	**Pregnancy**	**resumption regular cycles**
Kiddy 1992(1)	13	1000 kcal/day	26-30 weeks	>25		5	9 of 11 with >5% weight loss resumed regular menses
Guzick 1994(2)	RCT<br>6 diet<br>6 waiting list	8 weeks 400kcal/day then<br>4 weeks "refeeding"	12 weeks	Mean BMI 40,6	4 in diet<br>0 in waiting list		
Clark 1995(3)	13	lifestyle program	26 weeks	≥ 30	12	5	
Hollmann 1996(4)	35	5000-10000 cal/ week reduction	32 (+/- 14 weeks)	>30	Not assessed	10	28
Huber-Buchholz 1999(5)	18<br>3 drop-outs	Diet and exercise program	24	>27	9	2	
Hoeger 2004 (6)	11 lifestyle only	500-1000 kcal/day deficit	24	>25	OR 8,97 at > 3% weight loss	2	
Tang 2006(7)	74<br>6 drop-outs	Dietary advice with<br>500 kcal reduction/day	24	>30		4	58% improvement in menses
Palomba 2007(8)	20	800 kcal deficit/day	24	30 - 35	5	2	

**Anovulatory(ov-) and ovulatory patients(ov+)**							
**article**	**subjects**	**intervention Kcal/day**	**duration in weeks**	**inclusion BMI**	**ovulation**	**pregnancy**	**resumption regular cycles**
Clark 1998(9)	87<br>20 drop-outs	lifestyle program	24	>35	60 of 67	-18 spontaneous<br>-34 after fertility treatment	
Crosignani 2003 (10)	33 -<br>27 ov-<br>6 ov+	1200 kcal/day	28	>25	15 of 27ov-	10	18 of 27ov-
Moran 2003(11)	45<br>14 drop-outs	1500 kcal/day	12	>35		3	11of 25 ov-

**Pre-pregnancy weight loss and pregnancy outcome**			
**article**	**subjects**	**intervention**	**outcome**
Glazer 2004(12)	702 with weight 200-249 lbs	Weight loss	Weight loss of > 10 lbs RR 0,5(95% CI 0.28-0.92) of developing gestational diabetes in subsequent pregnancy
Dixon 2005 (13)	79 –pre-operative weight 125kg ±20	Laparoscopic gastric bypass	After an average weight loss of 28kg - significant decrease in gestational diabetes, PIH and pre-eclampsia compared to matched obese cohort

RESEARCH PROTOCOL

Costs and effects of a structured lifestyle program in overweight and obese subfertile couples to prevent unnecessary treatment and improve reproductive outcome

PROTOCOL TITLE

Costs and effects of a structured lifestyle program in overweight and obese subfertile couples to prevent unnecessary treatment and improve reproductive outcome

**Protocol ID**	**ZonMW 50-50110-97-518**
**Short title**	**Women of Weight and Infertility**
**Version**	**5**
**Date**	**25-9-2013**
**/project leader**	***Dr. A. Hoek MD***<br>***Section of Reproductive Medicine***<br>***Dept. Obstetrics and Gynaecology UMCG***<br>***Hanzeplein 1***<br>***9700 RB Groningen***<br>***a.hoek@og.umcg.nl***<br>***T:050-3613152***<br>***F:050-3611806***
**Principal investigator(s)**<br>**(*Multicenter research: per site)***	***Dr. A. Hoek MD***<br>***Prof dr. B.W. Mol (AMC Amsterdam)***<br>***Prof dr. JA. Land (UMC Groningen)***<br>***Dr. H. Groen (Epidemiology, UMC Groningen)***<br>***Prof Dr. R.P. Stolk (Epidemiology UMC Groningen)***<br>***Dr. Ir. W. Bemelmans (RIVM Bilthoven)***<br>***Dr. P.G.A. Hompes (Free UMC Amsterdam)***<br>***Prof. dr N.S. Macklon (UMC Utrecht)***<br>***Dr. F.J.M. Broekmans (UMC Utrecht)***<br>***Prof. dr. F. van der Veen (AMC Amsterdam)***<br>***Drs. W.K.H. Kuchenbecker (Isala klinieken Zwolle)***

	Prof dr. F.M. Helmerhorst (UMC Leiden)<br>***Dr. J.W.M. Maas (UMC Maastricht)***<br>***Dr. E.M. Kaaijk (OLVG Amsterdam)***<br>***Dr. G.J.E. Oosterhuis (Medisch Spectrum Twente Enschede)***<br>***Dr. P.X.J.M. Bouckaert (Atrium Medisch Centrum Heerlen)***<br>***Drs. J. Schierbeek (Deventer Ziekenhuis Deventer)***<br>***Dr. Y. M. van Kasteren (Medische centrum Alkmaar)***<br>***dr. A.W. Nap, (Ziekenhuis Rijnstate Arnhem)***<br>***Dr. E.A. Brinkhuis, (MeanderMC, Amersfoort)***<br>***Drs. J.M. Burggraaff (Leveste ziekenhuis, Emmen)***<br>***Dr. C. Koks (Maxima Medisch Centrum)***<br>***Drs. A.S. Blankhart (st Antonius Ziekenhuis Nieuwegein)***<br>***Dr. D.A.M. Perquin (Medisch Centrum Leeuwarden)***<br>***Drs. R.J.A.B. Mulder (Laurentius Ziekenhuis Roermond)***<br>***Drs. E.T.C.M. Gondrie (Orbis Medisch Centrum Sittard)***<br>***Dr. J.M. Sikkema (ZGT Almelo)***<br>***Dr. J.P. de Bruin (Jeroen Bosch Ziekenhuis)***
**Sponsor**	***ZonMW***<br>***Laan van Nieuw Oost Indië 334***<br>***2593 CE Den Haag***<br>***T: 07-3495111***

**Independent physician(s)**	***Drs. D.H. Bogchelman***<br><br>***Dept. Obstetrics and Section Gynaecology UMCG***<br><br>***Hanzeplein 1***<br><br>***9700RB Groningen***<br><br>***d.h.Bogchelman@og.UMCG.nl***

PROTOCOL SIGNATURE SHEET

**Name**	**Signature**	**Date**
**Coordinating Investigator/Project leader/**<br>**Dr. A. Hoek MD, PhD.**<br>**Gynaecologist UMCG**	**Dr. A. Hoek MD PhD.**<br>**Gynaecologist UMCG**	**02-03-2010**

TABLE OF CONTENTS

1. INTRODUCTION AND RATIONALE 10
2. OBJECTIVES 12
3. STUDY DESIGN 13
4. STUDY POPULATION 13
4.1 Population (base) 13
4.2 Inclusion criteria 13
4.3 Exclusion criteria 13
4.4 Sample size calculation 13
5. TREATMENT OF SUBJECTS 14
5.1 Investigational product/treatment 14
6 METHODS 17
6.1 Study parameters/endpoints 17
6.1.1 Main study parameter/endpoint 17
6.1.2 Secondary study parameters/endpoints (if applicable) 17
6.1.3 Other study parameters (if applicable) 17
6.2 Randomisation, blinding and treatment allocation 18
6.3 Study procedures 18
6.4 Withdrawal of individual subjects 22
7. SAFETY REPORTING 22
7.1 Section 10 WMO event 22
7.2 Adverse and serious adverse events 23
7.3 Follow-up of adverse events 23
8 STATISTICAL ANALYSIS 23
8.1 Descriptive statistics 23
8.2 Univariate analysis 24
8.3 Multivariate analysis 24
8.4 Economic evaluation 24
9. ETHICAL CONSIDERATIONS 25
9.1 Regulation statement 26
9.2 Recruitment and consent 26
9.3 Objection by minors or incapacitated subjects (if applicable) 27
9.4 Benefits and risks assessment, group relatedness 27
9.5 Compensation for injury 27
10. ADMINISTRATIVE ASPECTS AND PUBLICATION 28
10.1 Handling and storage of data and documents 28
10.2 Amendments 28
10.3 Annual progress report 28
10.4 End of study report 29
11. REFERENCES and addendum 29-38

LIST OF ABBREVIATIONS AND RELEVANT DEFINITIONS

**ABR**	**ABR form (General Assessment and Registration form) is the application form that is required for submission to the accredited Ethics Committee (ABR = Algemene Beoordeling en Registratie)**
**AE**	**Adverse Event**
**BMI**	**Body mass index**
**CA**	**Competent Authority**
**CBO**	**Centrale begeleidingsorgaan**
**CCMO**	**Central Committee on Research Involving Human Subjects**
**COH**	**Controlled ovarian hyperstimulation**
**CV**	**Curriculum Vitae**
**DEBQ**	**Dutch eating behaviour questionnaire**
**EU**	**European Union**
**FFQ**	**Food frequency questionnaire**
**FSH**	**follicle-stimulating hormone**
**FS-36**	
**GCP**	**Good clinical practice**
**GGD**	**Gemeentelijke Gezondheidsdienst**
**GOAL**	**Groninger Overweight And Lifestyle study**
**HCG**	**Human Choriongonadotrofine**
**HELLP**	**Syndrome with Haemolysis, Elevated Liver Enzymes and Low Platelets**
**HSG**	**Hysterosalpingography**
**HOMA**	**Homeostasis Model Assessment (measuring Insuline resisance)**
**IC**	**Informed Consent**
**ICSI I**	**Intra cytoplasmic sperm injection**
**RB**	**Institutional review board**
**IUI**	**Intra-uterine insemination**
**IVF-ET**	**In vitro fertilisation and embryo transfer**
**KCal**	**Kilo Calory**
**LH**	**Luteinizing hormone**
**METC**	**Medisch ethische toetsing commissie**
**NICE**	**Natinal Institute of Clinical Excellence UK**
**NIH**	**National Institute of Health USA**
**NVOG**	**Nederlanse Vereniging voor Obstetrie en Gynaecologie**

**OI**	**Ovulation Induction**
**PAM**	**Physical activity meter**
**PCOS**	**Polycystic ovary syndrome**
**RDC**	**Remote Data Capture**
**SMB**	**Safety Monitoring Board**
**(S)AE**	**Serious Adverse Event**
**SHBG**	**Sexhormone binding globulin**
**SUSAR**	**Suspected Unexpected Serious Adverse Reaction**
**TSH**	**Thyroid stimulating hormone**
**Wbp**	**Personal Data Protection Act (in Dutch: Wet Bescherming Persoonsgevens)**
**Wc**	**Waist circumference**
**WHO**	**World health organization**
**WHR**	**Waist hip ratio**
**WMO**	**Wet medisch onderzoek**

SUMMARY

Rationale: Subfertility affects approximately one in ten couples planning conception. Among subfertile women, about 30% are overweight or even obese. Epidemiological data suggest that the reduction of overweight will increase the chances of conception, decrease pregnancy complications and improve perinatal outcome. In small intervention studies beneficial reproductive effects of improvement of lifestyle leading to a reduction in body weight have been demonstrated. The British Fertility Society advises that fertility treatment be withheld if the body mass index (BMI) is over 35.
In the Netherlands, there is at present no agreed standard of care for subfertile women with overweight or obesity. Some centres simply withhold treatment of couples in whom the woman is overweight or obese, but most fertility centres treat overweight or obese women irrespective of their BMI. In a few centres, support is offered to women to help them lose weight.

Objective: In view of this lack of evidence and strong practice variation we propose a randomized clinical trial in overweight and obese subfertile women, in which we compare the costs and effects of a six months structured lifestyle program, aimed at weight loss, to “usual care”. The intervention aims to prevent unnecessary fertility treatment, complications associated with fertility treatment and obesity related pregnancy complications, thus improving pregnancy chances and perinatal outcome.

Study design: Multicenter randomised clinical trial.

Study population: Subfertile women (age 18-39) with a BMI of 29 kg/m² or above. Exclusion criteria are: azoospermia or donor semen, severe endometriosis, chronic anovulation due to WHO III and endocrinopathies. Women with an untreated preexistent hypertension or diabetes type 1, or pregnancy induced hypertension, preeclampsia, eclampsia or HELLP syndrome in a previous pregnancy are also excluded.

Intervention: The intervention consists of a structured lifestyle program of six months, which aims at realistic weight loss of at least 5% to10%, achieved by the combination of a healthy diet (caloric reduction of 600kCal/day), increase of physical activity (aiming at 10.000 steps per day) and two to three times a week sporting activity, and behavioural modification. The structured lifestyle program, in which practice variation is minimized using a structured software program, has been developed, used and evaluated previously (Zon-MW project 50-50110-98-078; the Groninger Overweight And Lifestyle study (GOAL)). Following six months of lifestyle intervention patients will start fertility treatment as indicated in the “usual care” group.
In the “usual care” arm fertility treatment will be started if this is justified by the individual prognosis (guideline NVOG)
All randomised women will fill in questionnaires concerning: diet, eating behaviour, physical activity and smoking: SF-36, FFQ, DEBQ, Squash list, at inclusion, and at 12 weeks, 6 months, 12 months and 24 months after randomisation. Blood samples (10 cc) will be taken at the start, 3 months and 6 months after the start of the study. Cord blood will be taken from approximately 150 patients after childbirth to assess the epigenetic and metabolic profile. The data will also be used in the follow-up study in case women give informed consent to participate in the follow up study of her child: “the preconception weight loss; does it affect infant health study” (METc: 2011/134), (the so-called “Lifestyle-kids” study).

Main study parameters/endpoints:
Primary endpoint is the birth of a healthy singleton after vaginal delivery of at least 37 weeks gestation.
Secondary outcome parameters are number of fertility treatments (OI, IUI, IVF, ICSI), quality and amount of oocytes and embryos and cryo embryos derived in an IVF or ICSI procedure, the amount of embryos per transfer, implantation rate of transferred embryos, complications of fertility treatment, clinical and ongoing pregnancy rates, perinatal outcome, complications and quality of life as well as body weight, waist circumference, behaviour influencing weight, i.e. nutritional habits and exercise pattern, smoking habits, blood pressure, glucose/insulin ratio (HOMA), hormonal profile and costs.

DATA ANALYSIS: Analysis will be by intention to treat. Randomisation will be stratified for the presence or absence of anovulation and per treatment center. We expect an

improvement in vaginal singleton delivery beyond 37 weeks from 45% to 60%. Anticipating drop out of 20% in the intervention arm ad a 5% loss to follow up in the study, we need to include two groups of 285 women (two-sided test, alpha error.05, beta-error .20).

STUDY DURATION: Preparation of the study, including the training of the nurses, will take three months. Inclusion of the couples will take 24 months and the lifestyle intervention will take six months. Follow up is continued for 24 months after randomisation. Database cleaning and analysis of the study will take 6 months. Duration of the study: 57 months

Nature and extent of the burden and risks associated with participation, benefit and group relatedness:

The advantages of a weight loss and exercise program in overweight or obese subfertile women have been shown in small intervention studies. Even if the patient does not conceive during the 6 months period in the lifestyle intervention arm, the health benefits of weight loss on the long-term are clearly in the interest of the patient.

By starting this project, fertility treatment in the intervention arm will be delayed for a maximum of 6 months, there is no evidence that this delay will cause lower spontaneous or treatment dependent pregnancy chances. A more structured weight loss and exercise program will probably lead to higher spontaneous and therapy induced ongoing pregnancy chances.

The Burden: Patients that are randomized in the intervention arm will attend the clinic for six extra visits in order to improve lifestyle, this will take around 280 minutes. In between visits counselling will take place per telephone or mail four times for 15 minutes. During these visits physical examination, measuring weight, WC and WHR in week: one, three, seven, twelve, eighteen and twenty-four will take place. Patients in the intervention group will have to adapt there lifestyle towards a more healthy lifestyle, this will give a certain amount of stress since they will have to adapt their diet and physical activity. Patients will be learned how to use stepcounter PAM and wear it on a daily basis in order to monitor their physical activity during the intervention period and will fill in their activity list.

Patients who have been randomized in the “usual care” group will undergo fertility treatment when indicated by national guidelines (www.nvog.nl). Patients with overweight or obesity have additional risks during pregnancy compared to normal weight women. The risks involve: hypertension (OR: 2.2), preeclampsia (OR: 1.8-2.7), pregnancy-induced diabetes (OR: 2.2-3.4), caesarian section (OR: 1.3-2.4) (see tables of evidence in appendix). These risks are not influenced by fertility treatment. Lifestyle intervention aims to reduce the involved pregnancy risks.

An independent Safety Monitoring Board (SMB) will be installed to review major complications in both treatment groups. This board will evaluate complications after every 150 included patients and six months and 12 months after the end of inclusion of the study (6 times during the study). The SMB will differentiate between major en minor complications, blinded for treatment arm. Every case of preeclampsia, eclampsia, HELLP syndrome, maternal or infant mortality will be reported to the SMB. The SMB findings will be reported to

the Ethics Committee of the UMCG

Questionnaires to be filled in by all participants in the study: SF-36, SQUASH-list, FFQ, DEBQ, concerning eating behaviour at the start, week twelve, twenty-four and fifty-two of the study. A questionnaire for details on direct and indirect costs like transportation, sporting activity and productivity loss due to lifestyle intervention programme and fertility treatment will be filled in by the participants during the study. This will take 30-45 minutes.

1. INTRODUCTION AND RATIONALE

The evidence for the adverse effects of overweight on women's reproductive health is indisputable. Overweight affects reproductive capacity in all subfertile couples. Moreover, overweight is associated with pregnancy complications and poor neonatal outcome. Pregnancy complications associated with obesity are hypertensive disorders, gestational diabetes, prolonged duration of labour, increased need of operative delivery, macrosomia, shoulder dystocia and increased blood loss (Garbaciak et al.,1985; Edwards et al.,1996). Obesity is associated with an increased risk of adverse pregnancy outcomes such as unexplained still birth (Cnattingius et al.,1998; Kristensen et al.,2005; Bergstrom et al.,1998; Linne et al.,2004, neonatal admissions (Usha Kiran et al.,2005) and five times higher costs (Linne et al.,2004).

Each year about 15.000 to 20.000 new couples present themselves with subfertility. In a large Dutch cohort study of 3.000 subfertile couples (Van der Steeg et al.,2007), 19% of the women had a BMI between 25 and 30, 7% had a BMI between 30 and 35, and 4% above 35. Thus, approximately 30% of these couples are overweight or obese.

Obesity has further been associated with reduced fertility in the general population (Ramlau-Hansen et al.,2007). Ovulatory subfertile women with a BMI of 29 kg/m2 or higher have a 4% lower pregnancy rate per kg/m^2 increase (Van der Steeg et al., 2007). Obese women also have a lower live birth rate after IVF and ICSI (Norman et al.,1998; Wang et al.,2000; Wang et al.,2002; Fedorcsak et al.,2004, Lintsen et al.,2005).
The relationship between BMI and the risk of spontaneous abortion is inconsistent (Lashen et al.,1999; Fedorczak et al.,2000; Wittemer et al.,2000; Wang et al.,2001, Lintsen et al.,2005). A meta-analysis on the effect of overweight and obesity on ART reported a lower chance of pregnancy following IVF (OR 0.71, 95% CI:0.620-0.81) and an increased miscarriage rate (OR 1.3, 95% CI:1.06-1.68). Extrapolating from available data one could argue that the combination of lower chance of pregnancy and higher miscarriage rate in overweight and obese women could result in a reduced live birth rate (Maheshwari et al.,2007).

Obesity is strongly related to polycystic ovary syndrome (PCOS), which is the cause of subfertility in 25% couples. The accompanying insulin resistance and hyperinsulinism mark this syndrome as a prediabetic state (Dunaif, 1997), leading to an increase of neonatal complications.

In reproductive medicine, modest weight reduction (5-10%) with lifestyle intervention is not only associated with restoration of ovulation and fertility in overweight women with PCOS (Hoeger et al.,2004) but is also shown to decrease insulin resistance (Pasquali et al.,1989; Knowles et al.2002). Several studies have shown that even a modest weight loss leading to a 5% loss of central fat, in women with PCOS improves, the menstrual cycle, the rate of ovulation, and the likelihood of a healthy pregnancy (Clark et al.,1995, Norman et al.,2004, Huber-Buchholz et al,.1999, Stamets et al., 2004).

In view of the evidence that overweight has a negative impact on fertility and pregnancy outcome, lifestyle intervention prior to treatment of subfertile couples could improve spontaneous fertility chances, and prevent unnecessary fertility treatment as well as complications of fertility treatment.

Several reviews showed that lifestyle interventions targeted at changing diet and physical activity can succeed in sustained decrease of average bodyweight on the longer term (McTigue, 2004; Franz 2007). A decrease of 3 to 4 kilograms can be achieved after three years of follow-up (Avenell, 2004). The adverse health effects of increased body weight on for instance development of diabetes mellitus type 2, coronary heart disease, musculoskeletal disorders and some types of cancer are well known (WHO-euro document 2007). The cost-effectiveness of lifestyle interventions was below acceptable limits (Vijgen, 2006). For the Diabetes prevention project, for instance, the costs to prevent one new case of diabetes mellitus were calculated to be around 16.000 US dollars (Herman, 2005).

The intervention we propose to evaluate is based on dietary therapy, increased physical activity and individualized behavioural modification plan. A combination of these three interventions leads to maximal weight loss and maintenance of weight loss (NIH guideline, CBO guideline 2007, Zelissen et al 2004). This behavioural modification therapy can be delegated to a nurse practitioner or trained nurse or dietician. Such an intervention program can be implemented within gynaecological care.

Although lifestyle modification is advised as a key component for the improvement of reproductive function in overweight women specifically with PCOS (NICE 2004, Balen et al., 2006 Tang et al.,2006, Pasquali et al.,1989, Hoeger et al.,2004, Clark et al.,1995 and 1998), the evidence of its effectiveness as demonstrated in clinical studies is limited. It's cost-effectiveness has never been assessed in large groups of subfertile women with respect to prevention of unnecessary fertility treatment, prevention of complications of fertility treatment and improvement of perinatal outcome.

The cost-effectiveness of such program will be investigated in the this study.
(see table Summary of studies assessing outcome after lifestyle intervention).

2. OBJECTIVES

In view of the lack of convincing evidence from large intervention studies and the strong practice variation in The Netherlands we propose a randomized clinical trial in overweight and obese subfertile women, in which we compare the costs and effects of a six months structured lifestyle program, aimed at weight loss, to "usual care" with the aim to prevent obesity related pregnancy complications, unnecessary fertility treatment and complications associated with fertility treatment, enhance pregnancy chances and improve perinatal outcome. The control group of women will receive fertility treatment "care as usual" without lifestyle intervention.

We aim to estimate the costs and effects of a structured lifestyle program in terms of costs per additional healthy singleton birth rate beyond 37 weeks gestation born after vaginal delivery in a follow up period of 24 months.

The questions that we aim to answer are:

Primary Objective:
Does a structured lifestyle program aiming at a weight reduction of 5%-10% reduce the need for fertility treatments due to an increase in spontaneous conception rate and treatment dependent or treatment independent life birth rate?

Secondary Objective(s):
Does a structured lifestyle program aiming at a weight reduction of 5%-10% increases the success rates of fertility treatments, including assisted reproductive technology?

Does a structured lifestyle program aiming at a weight reduction of 5%-10%, lead to less maternal complications during fertility treatment or pregnancy, such as hypertensive disorders, premature birth, macrosomia, shoulder dystocia, operative delivery, and haemorraghia, and multiple pregancies in subfertile women with overweight or obesity?

Does a structured lifestyle program aiming at a weight reduction of 5%-10% increases the quality and number of oocytes and embryos and cryo embryos resulting from an IVF or ICSI procedure and increases the amount of embryos per transfer and the implantation rate of transferred embryos ?

Does a structured lifestyle program aiming at a weight reduction of 5%-10% lead to a lower percentage of women with overweight or obesity and subfertility developing gestational diabetes?

Does a structured lifestyle program aiming at a weight reduction of 5%-10% in subfertile women, lead to a better quality of life, as well as body weight reduction, reduction of waist

circumference, behaviour influencing weight, i.e. nutritional habits and exercise pattern, blood pressure, serum glucose/insulin ratio (HOMA) and hormonal profile changes.

3. STUDY DESIGN

Multicenter randomised clinical trial.
Flow chart 1 gives an overview of the study procedure form randomization till end of follow up period.
Table 1 is added to give an overview of the lifestyle intervention that subjects will undergo in the course of research.

STUDY MANAGEMENT: The randomisation is performed by computer at a central randomisation centre in the AMC. Randomisation will be stratified according presence or absence of anovulation and center of treatment.
Data will be collected using Oracle Clinical Remote Data Capture (RDC), which is a new generation of application system that enables collection and cleanup of clinical trial data using the Internet. For detailed information on Oracle RDC, please visit the page of Oracle RDC products (http://www.ctc-g.co.jp/en). The expertise for this technology is already used in the study group.

4. STUDY POPULATION

4.1 Population (base)

All couples suffering from subfertility visiting the clinics participating in the study will undergo a basic fertility work-up including a semen-analysis, an assessment of tubal patency using CAT or HSG, and monitoring of the cycle to assess ovulation. After the work-up has been completed, a fertility diagnosis will be made and a prognosis for treatment independent pregnancy will be calculated (Hunault et al.,2004), followed by a management proposal for the individual couple.
Ovulatory and anovulatory women with a body mass index between 29 kg/m² and 40 kg/m² will be approached to participate in the present study. After informed consent, they will be randomly allocated to an intervention program to improve lifestyle with a duration of six months, or to “usual care”.

4.2 Inclusion criteria

We aim to include subfertile women with a BMI of 29 kg/m² or above. Women have to be between 18 and 39 years old.
Subfertility is defined as failure to conceive within 12 months of unprotected intercourse in couples(where the woman has an ovulatory cycle), as well as couples where the women suffers from chronic anovulation due to WHO I or II or PCO.

4.3 Exclusion criteria

Couples suffering from azoospermia or donor semen, severe endometriosis AFS 3 and 4 (Revised American Fertilty Society classification), chronic anovulation due to WHO III, endocrinopathies (like: cushing syndrome, adrenal hyperplasia, hypothyroidism, diabetes mellitus type I) will not be eligible for the study. Women with an untreated preexistent

hypertension or diabetes, or pregnancy induced hypertension, preeclampsia, eclampsia or HELLP syndrome in a previous pregnancy are also excluded.
Incapacitated adults will be not asked to participate in the study.

Women eligible for the study will be referred to a research nurse. This person is dedicated to counselling eligible patients and will perform randomisation and collect follow-up data, but will not perform the experimental intervention. The lifestyle intervention programme will be done by trained intervention nurses. We will collaborate with the research nurses who are involved in the obstetric and urogynaecological consortium. In this consortium, more than 20 large randomised clinical trials are performed. Information on this infrastructure is obtainable from http://www.studies-obsgyn.nl/

4.4 Sample size calculation

SAMPLE SIZE: On the basis of the literature the cumulative live birth rate in a follow up of two years of subfertility treatment will be 45% for the “usual care” group (Dutch OFO-study, unpublished data). The lifestyle intervention group is estimated to have a life birth rate of 60% (Dutch OFO-study, unpublished data)

We expect an improvement of healthy singleton of more than 37 week gestation born after vaginal delivery from 45% to 60% in the intervention group. Drop out is expected to be 20% in the intervention arm and is included in the analysis of effectiveness.

To prove that, we need to include two groups of 272 women to be included (alpha 0.05, power 80%). To account for 5% lost to follow up 285 women will be included per group. In total 570 women will be included.

5. TREATMENT OF SUBJECTS

5.1 Investigational treatment

THE “USUAL CARE” ARM: In the “usual care” arm treatment will be started if the individual prognosis, based on the Hunault model (Hunault et al.2004) is less than 30% chance of conception within one year or when more than 3 years subfertility (guideline NVOG) justifies starting treatment. Even so, in case of chronic anovulation, ovulation induction will be started. Fertility treatment can either be IUI, IVF or ICSI whatever is indicated according to the Dutch guideline on IVF treatment (NVOG). In anovulatory patients, ovulation induction will be started using clomiphene, or gonadotrofines, which is applicable. When the Hunault model shows a prognosis of more than 30% pregnancy chance in the forthcoming year (and patients are less than three years subfertile), expectant management will be offered.

THE EXPERIMENTAL ARM: The overweight intervention is based on the lifestyle module which is developed in ZONMW project (50-50110-98-078) and will be adapted for the study

proposal in this specfic group of subfertile woman for a duration of six months. The software module developed, used and evaluated in this program will be adapted for this subfertile patient group. The use of the software program will reduce practice variation between fertility centres. The lifestyle intervention is based on the recommendations of Dutch CBO guideline "Diagnostiek en behandeling van obesitas bij volwassenen en kinderen" and consists of a structured lifestyle program targeted at:

1. Changing the dietary pattern.
2. Stimulating physical activity of moderate intensity.
3. Changing existing behaviour.
4. Self- monitoring.
5. Involvement of the partner.
6. Referral to psychologist when indicated.

ad 1. CHANGING DIETARY PATTERN

Women will be advised to adapt their dietary pattern and sustain a healthy diet with a caloric reduction of approximately 600kcal compared to their normal caloric intake (but not below 1200kcal/day). A realistic target weight will be set of 5-10% below their body weight. The diet should be based on the Dutch guideline "Gezonde voeding" in order to achieve a sustainable diet change.

ad 2. STIMULATING PHYSICAL ACTIVITY

Physical activity is necessary in order to sustain a modest weight loss and increase effect of dietary measures. Patients will use a stepcounter (PAM) to measure daily steps aiming for a daily goal of 10.000 steps. Two to three times a week sporting of at least 30 minutes of moderate intensity (60-85% of maximum hearth frequency) is advised. Sporting advice is individualized taking into account a woman's preference and possibilities (swimming, walking, stepping, cycling, cardio-fitness, etc.). Patients keep a diary to not the result of the stepcounter, and the sporting activities. These results are evaluated by the trained nurses.

ad 3. CHANGING EXISTING BEHAVIOUR

The motivation to change lifestyle is measured and evaluated during the program with the PACE score measuring stages of change (precontemplative stage, considering -preparatory stage, action stage and maintenance stage). This PACE score is part of the software module “GOAL”. Motivational counselling is individualized for these stages of change. Changing existing behaviour is achieved by motivational counselling directed at:

-Awareness of actual lifestyle leading to overweight or obesity.

-Counselling healthy lifestyle measures, the effect of healthy lifestyle in relation to subfertility and spontaneous and treatment dependent pregnancy chances as well as pregnancy complications and influence on perinatal outcome.

-Individualized goals are formulated and embedded in a "patient contract". "Patient contracting" is a tool to actually individualize the measures that the patient will take and to stick to the goals and targets that are agreed upon. During the intervention individual goals will be evaluated, feedback will be given and goals adapted when necessary. Continuous

evaluation and feedback will be maintained focussed on the target bodyweight and long term maintenance of lifestyle changes.

ad 4. SELFMONITORING

Self-monitoring is an essential tool to improve compliance during a lifestyle program. It will be implemented using a PAM "stepcounter" to measure daily physical activity and the "eetmeter" (www.voedingscentrum.nl). This webbased tool is used to give feedback on food intake, and caloric intake on a daily basis. Patients will be trained to use this device. A diary with sporting activities as well as caloric intake will be evaluated by the dedicated nurses.

ad 5. INVOLVEMENT OF THE PARTNER

Involvement of the partner and family of the women is important in order to guarantee social support and a "buddy system" during lifestyle changes.

ad 6. REFERRAL

When indicated, referral to psychologist or intervention available in the general health care system will used.

Patients undergoing the lifestyle program will be guided and supported by trained nurses or dieticians or nurse practitioners, who will be trained prior to the study. Women who miss two consecutive sessions and who are not willing to catch up in another group are excluded from the program.

The standardized lifestyle program consists of four sessions in the first three months, and than a booster of two sessions in the last three months. Telephone consultation or consultation by mail is scheduled in between these sessions four times. (see table 1)

FOLLOW-UP:

All participating couples (usual care arm and the experimental care arm) will complete several questionnaires, i.e. the SF-36: measuring satifaction, the SQUASH list: for physical activity, the FFQ: food frequency questionnaire for assessing nutrient intake): a structured questionnaire about food pattern (e.g. breakfast yes/no) and important food components (e.g. snacks, fruits and vegetables and meat), the Dutch Eating Behaviour Questionnaire (DEBQ): for assessment of restrained, emotional and external eating behaviour (van Strien 1986). Finally, several questions are completed i.t.o. history of body weight, e.g. the duration of overweight, weight fluctuations.

Questionnaires are webbased. In case a patient does not use the computer paper questionnaires will be used. Questionnaires will be completed at start, 12 weeks, 24 weeks and 52 weeks after randomisation. In addition each woman will receive a questionnaire for details on associated direct costs of professional care and on indirect costs like transportation, sporting activity and productivity loss. These questionnaires will be handed out in consultation 1, week 1 of the lifestyle module or at the start of fertility treatment for

women that were randomised for “usual care”. This will be repeated at the time points of the other questionnaires (12, 24 and 52 weeks after randomisation and at the end of follow-up).

Finally, patients who get pregnant during the period up to 24 months after randomisation will receive a questionnaire in which they can register their (self-reported) weight-gain during pregnancy. To assess gestational diabetes, an oral glucose tolerance test is advised at 28-30 weeks of gestation. Cord blood will be taken from approximately 150 patients after childbirth to assess the epigenetic and metabolic profile. The data will also be used in the follow-up study in case women give informed consent to participate in the follow up study of her child: “the preconception weight loss; does it affect infant health study” (METc: 2011/134), (the so-called “Lifestyle-kids” study).

Moreover, we will register reproductive outcome, fertility treatments as well as the course and outcome of subsequent pregnancies, including obstetrical interventions for a period up to 24 months after randomisation.

6. METHODS

6.1 Study parameters/endpoints

Background and Demographic Characteristics
To assess whether the treatment groups were balanced, the study populations will be compared for baseline measurements including female age, education, ethnicity, type of infertility (primary/secondary), duration of infertility, intoxications, body mass index, as well as sperm analysis according to WHO standards.

6.1.1 Main study parameter/endpoint

The primary endpoint will be a healthy singleton of more than 37 week gestation born after vaginal delivery. The initial analysis, will be done by intention to treat, so we will not make a difference for treatment independent pregnancies and pregnancies that occurred after treatment. Patients who will drop out of the study will by analysed according to their group of randomisation. In a per protocol analysis drop outs will be asked to provide information on the primary outcome, i.e. life birth within two years after randomisation. These drop outs will be analysed as non-compliant.

6.1.2 Secondary study parameters/endpoints

Secondary outcome parameters are:
1. pregnancy outcome and -complications: miscarriage, gestational diabetes, Pregnancy induced hypertension, preeclampsia, HELLP, prematurity<37 weeks, macrosomia (>p90

(Large for Gestational Age), induction of labour, prolonged duration of labour, operative delivery, assisted delivery and increased blood loss, and multiple pregnancies
2. percentage of women needing fertility treatment in both groups (OI, IUI, IVF, ICSI) and ongoing pregnancy rates in these treatments.
3. Quality and number of oocytes and embryos and cryo embryos resulting from an IVF or ICSI procedure and the amount of embryos per transfer and implantation rate of transferred embryos
4. complications during fertility treatment: ovarian hyperstimulation syndrome, infection, tubal pregnancies, torsion (etc).
5. perinatal outcome: stillbirth, pregnancy duration, body weight, Apgar scores, arterial pH, congenital anomalies and neonatal admission to a neonatal medium, high or intensive care unit..

6. quality of life, pre-pregnancy body weight, weight gain during pregnancy, waist circumference, behaviour influencing weight, i.e. nutritional habits and exercise pattern, blood pressure, glucose/insulin ratio (HOMA), hormonal profile (androgens, adipokines and cytokines) and costs.

6.1.3 Other study parameters

ECONOMIC EVALUATION: The aim of the economic evaluation is to compare the costs and health effects of a lifestyle program versus “usual care”. The analysis will be a cost-effectiveness analysis with the proportion of healthy singleton of more than 37 week gestation born after vaginal delivery as the primary outcome.

6.2 Randomisation, blinding and treatment allocation

The randomisation is performed by computer at a central randomisation centre in the AMC. Randomisation will be stratified according to presence or absence of anovulation and center of treatment.
Women eligible for the study will de referred to a research nurse. This person is dedicated to counselling eligible patients and will perform randomisation and collect follow-up data, but will not perform the experimental intervention. We will collaborate with the research nurses who are involved in the obstetric and urogynaecological consortium. In this consortium, eight large randomised clinical trials are performed. Information on this infrastructure is obtainable from http://www.studies-obsgyn.nl/

6.3 Study procedures

RANDOMISATION BY RESEARCH NURSE
Baseline measurement of body weight, length, waist, hip and blood pressure for all patients.
A blood sample of 10 cc will be taken and serum stored of all patients.

Handout and introduction of the "eetmeter" and stepcounter and food and physical activity diary for the patients randomised for lifestyle intervention.
Website www.voedingscentrum.nl/ eetmeter is explained.

TIME SCHEDULE LIFESTYLE PROGRAM
(see flow chart I: TIME SCHEDULE LIFESTYLE PROGRAM)

WEEK 1: BASELINE EVALUATION.
Consultation 1.
Counselling session 45-60 minutes.
Motivational counselling will include an extensive history of weight changes, knowledge and insight of weight related health problems.
Awareness, actual behaviour and lifestyle leading to overweight and history of body weight and slimming are discussed.
Counselling negative effect of overweight on frequencies of spontaneous and treatment dependent pregnancy chances and increase frequency of complications during pregnancy for mother and foetus.
The stages of change can be identified using the PACE score in the GOAL software.
DEBQ is evaluated to asses the type of eating behaviour.
Counselling and providing knowledge and advice about healthy lifestyle.
Evaluating diet questionnaire
Evaluating SQUASH score for physical activity
Healthy diet is advised aiming for a reduction of 600 Kcal/day (“Energy reduced diet”). Diet should be based on the Dutch guideline “Gezonde Voeding”. Food intake should not be reduced below 1200 Kcal/day in order to achieve a sustainable lifestyle change.
Use of stepcounter is explained: 10.000 steps/day and two to three times a week sporting of at least 30 minutes of moderate intensity (60-85% of maximum hearth frequency).
A questionnaire for details on indirect costs like transportation, sporting activity and productivity loss will be handed out.
Partner is involved in the counselling session
Referral to a dietician or psychologist is done in a standardized way, when appropriate.
Measuring waist circumference and weight.

WEEK 3: SETTING REALISTIC TARGETS AND GOALS.
Consultation 2.
Counselling session of 45-60 minutes.
Focus on agreeing on achievable targets on an individual basis regarding physical activity, diet and body weight.
Food -and physical activity diary including results of "eetmeter" and stepcounter are evaluated. Diet: reduction of 600 Kcal/day (but not below 1200 Kcal/day)is introduced.
Physical activity aiming at 10.000 steps a day.
Patient contract describing realistic goals c.q. agreements on an individual basis regarding: weight loss (…. Kg/ …. Weeks) and physical activity (…. Steps/ …. day), and sporting activity (two to three times a week),
Plan activities to reach goals.
Address barriers and promote facilitating factors to achieve sustainable lifestyle changes.
Measuring waist circumference and weight.

<u>WEEK 5. CONSULTATIONS VIA TELEPHONE OR MAIL (SEE BELOW)</u>

WEEK 7: EVALUATION AND FEEDBACK
Consultation 3.
Counselling session of 30 minutes.
Regarding: motivation weight loss and physical activity.
Evaluating results regarding: bodyweight, food intake diary, "eetmeter", physical activity diary, stepcounter
Feedback on results.
Individualized advice for improvement when necessary and adjust agreements when necessary to reach realistic targets.
Discuss relapse.
Evaluating the barriers to follow the program when necessary.
Referral to a dietician or psychologist is done in a standardized way, when appropriate.
Measuring waist circumference and weight.

<u>WEEK 8. CONSULTATIONS VIA TELEPHONE OR MAIL (SEE BELOW)</u>

WEEK 12: EVALUATION AND FEEDBACK
Consultation 4.
Counselling session of 15-30 minutes.
Regarding: motivation weight loss and physical activity.
Evaluating results regarding: bodyweight, food intake diary, "eetmeter", physical activity diary, stepcounter.
Feedback on results.
Individualized advice for improvement when necessary and adjust agreements when necessary to reach realistic targets.
Discuss relapse.
Evaluating the barriers to follow the program when necessary.
Referral to a dietician or psychologist is done in a standardized way, when appropriate
Measuring waist circumference and weight.
A blood sample of 10 cc will be taken and serum stored

<u>WEEK 15. CONSULTATIONS VIA TELEPHONE OR MAIL (SEE BELOW)</u>

WEEK 18 EVALUATION AND FEEDBACK
Consultation 5.
Counselling session of 15-30 minutes.
Regarding: motivation weight loss and physical activity
Evaluating results regarding: weight, food diary, eetmeter, physical activity, stepcounter, -
Feedback on results.

Individualized advice for improvement when necessary and adjust agreements when necessary to reach realistic targets.
Discuss relapse.
Evaluating the barriers to follow the program when necessary.
Referral to a dietician or psychologist is done in a standardized way, when appropriate.
Measuring waist circumference and weight.

WEEK 21. CONSULTATIONS VIA TELEPHONE OR MAIL (SEE BELOW)

WEEK 24 EVALUATION OF TARGETS
Consultation 6.
Counselling session of 15-30 minutes.
Evaluation of targets:
Weight reduction
Physical activity
Behaviour change
Measuring waist circumference and weight.
A blood sample of 10 cc will be taken and serum stored.

CONSULTATIONS VIA TELEPHONE OR MAIL WEEK 5, 8, 15, AND 21.
In between the consultations in the outpatient fertility clinic, consultation via telephone or mail are scheduled at week 5, week, 9, week 15, and week 21 with a duration of 15 minutes. In these consultations evaluation regarding: motivation of weight loss and physical activity are discussed
Evaluating results regarding: weight, food diary, eetmeter, physical activity, stepcounter.
Feedback on results.
Individualized advise for improvement when necessary and adjust agreements when necessary to reach realistic targets.
Discuss relapse.
Evaluating the barriers to follow the program when necessary.

Questionnaires for all included women: SF36, FFQ and Squash-list will be evaluated at the start and week 12 and week 24 and 52 weeks after randomisation.

Start of fertility treatment
When in spontaneous ovulatory patients in the lifestyle intervention arm:

- have finished their six month lifestyle program or,
- when they meet their target weight reduction of 5-10% or,
- when their weight decreases < BMI 29 kg/m²,

fertility treatment will be started if the individual prognosis, based on the Hunault model (Hunault et al.2004) if less than 30% chance of conception within one year or when more than 3 years subfertility (guideline NVOG) justifies starting treatment (either IUI, IVF or ICSI (according to national guidelines NVOG).

In case of chronic anovulation, ovulation induction will be started when patients in the lifestyle intervention arm:

- have finished their six month lifestyle program or,
- when they meet their target weight reduction of 5-10% or,
- when their weight decreases < BMI 29 kg/m^2.

Table 1: time schedule lifestyle intervention

Week	Consultation Outpatient/ Telephone or mail	Counseling subject lifestyle program	Duration minutes
	Randomisation	Baseline assessment and explanation lifestyle program; handout of “eetmeter”, stepcounter and diary . Blood sample (10 cc)	30
1	Outpatient 1	Setting targets and planning goals; evaluating baseline measures , hand out of cost-questionnaires	45-60
3	Outpatient 2	Evaluating targets;	45-60
5	Telephone 1	Evaluating targets	15
7	Outpatient 3	Evaluating targets	30
9	Telephone or mail 2	Evaluating targets	15
12	Outpatient 4	Evaluating targets, handout of questionnaires Blood sample (10 cc)	30
15	Telephone or mail 3	Evaluating targets	15
18	Outpatient 5	Evaluating targets	30
21	Telephone or mail 4	Evaluating targets	15
24	Outpatient 6	Assessing targets and evaluation lifestyle program and handout of questionnaire	30
>24	Outpatient	Start infertility treatment when applicable Blood sample (10 cc)	
52		questionnaires	

6.4 Withdrawal of individual subjects

Subjects can leave the study at any time for any reason if they wish to do so without any consequences. Patients who decide to withdraw from the study will be asked by the responsible investigator to provide information regarding the primary endpoint of the study 24 months after randomisation. Patients who drop out of the study will be treated according to the local protocols for infertility patients.
The investigator can decide to withdraw a subject from the study for medical reasons. Women who miss two consecutive counselling sessions and who are not willing to catch up are excluded from the program.

7. SAFETY REPORTING

7.1 Section 10 WMO event

In accordance to section 10, subsection 1, of the WMO, the investigator will inform the subjects and the reviewing accredited METC if anything occurs, on the basis of which it appears that the disadvantages of participation may be significantly greater than was foreseen in the research proposal. The study will be suspended pending further review by the accredited METC, except insofar as suspension would jeopardise the subjects' health. The investigator will take care that all subjects are kept informed.

7.2 Adverse and serious adverse events

Adverse and serious adverse events as a direct result of the lifestyle intervention are not anticipated in this study. However, patients with overweight or obesity have additional risks during pregnancy compared to normal weight women. As a result of both the intervention arm and of the "care as usual" arm, pregnancies may occur in patients with increased risks. The risks involve hypertension (OR: 2.2), preeclampsia (OR: 1.8-2.7), pregnancy-induced diabetes (OR: 2.2-3.4), caesarian section (OR: 1.3-2.4) (see tables of evidence in appendix). These risks are not influenced by fertility treatment. Lifestyle intervention aims to reduce the involved pregnancy risks. Participants in the study have been informed about these risks.
An independent Safety Monitoring Board (SMB) will be installed to review major complications in both treatment groups. This board will evaluate complications after every 150 included patients and six months and 12 months after the end of inclusion of the study (6 times during the study). The SMB will differentiate between major en minor complications, blinded for treatment arm. Every case of preeclampsia, eclampsia, HELLP syndrome, will be reported to the SMB. The SMB findings will be reported to the Ethics Committee of the UMCG

Adverse events are defined as any undesirable experience occurring to a subject during a clinical trial, whether or not considered related to the investigation. All adverse events reported spontaneously by the subject or observed by the investigator or his staff will be recorded.
A serious adverse event is any untoward medical occurrence or effect that at any dose results in death;

- is life threatening (at the time of the event);
- requires hospitalisation or prolongation of existing inpatients' hospitalisation;
- results in persistent or significant disability or incapacity;
- is a congenital anomaly or birth defect;

- is a new event of the trial likely to affect the safety of the subjects, such as an unexpected outcome of an adverse reaction, lack of efficacy of an IMP used for the treatment of a life threatening disease, major safety finding from a newly completed animal study, etc.

All SAEs will be reported to the accredited METC that approved the protocol, according to the requirements of that METC.

7.3 Follow-up of adverse events

All adverse events will be followed until they have abated, or until a stable situation has been reached. Depending on the event, follow up may require additional tests or medical procedures as indicated, and/or referral to the general physician or a medical specialist.

8. STATISTICAL ANALYSIS

8.1 Descriptive statistics

To asses whether the groups were balanced the study population will be compared for baseline measurements including female age, type of subfertility (primary or secondary), duration of subfertility, intoxications, smoking, BMI, as well as sperm analysis according to WHO standards, anovulation, and subfertility treatment. Confounding factors, such as smoking, education, ethnicity will be addressed in the analysis.

8.2 Univariate analysis and multivariate analysis

The primary analysis will be by intention to treat.
The primary outcome will be analysed by comparing the pregnancy rates in both groups using Kaplan-Meier analysis of time to pregnancy using the Log-rank test. In this analysis, patients will be censured at the time they discontinue or complete the study without getting pregnant. In addition, pregnancy rates and 95% confidence intervals per group will be calculated based on the Kaplan-Meier estimates at various time points. Further analysis of delivery rates over time will be performed using Cox-regression analysis with correction for the stratification variables ovulatory status and treatment centers as well as for confounders. These analyses will also be performed for spontaneous and treatment-induced pregnancies separately. The same analysis will be performed for the secondary outcome ongoing pregnancies. This will include ongoing pregnancies, that have not yet resulted in the birth of a child. Exploratory subgroup analyses of the primary outcome will be performed for spontaneous and treatment-induced pregnancies and for women with a BMI below 35 versus above 35, as well as for anovulatory versus ovulatory women and for women with a waist-hip ratio of above 0.8 versus below or equal to 0.8 and for women who are 36 years or older versus younger than 36 years based on tests of interaction with treatment group.

Additional analysis concerning the influence of HOMA, androgens, adipokines and cytokines on pregnancy chances and recovery of ovulations in anovulatory patients will be assessed using multivariate Cox-regression**.**
Incidence of complications of treatment and during pregnancy will be compared in both groups using relative risks and 95% confidence intervals**.**
Quality of life will be analysed using repeated measures analysis of variance

A per protocol analysis will also be performed, in which drop out patients will be identified as non-compliant. Information on the primary outcome, i.e.lifebirth within two years after randomisation will be used whenever provided. Patients who drop out of the lifestyle intervention arm will be analysed in the "usual care" arm in this per protocol analysis.

Women who drop out of the study will be asked to provide the reason for dropping out. This reason will be recorded and patients will be asked to provide information regarding the primary outcome, i.e. pregnancy within two years after randomisation. In addition, patients will be offered the possibility to continue to complete cost questionnaires for the remainder of the study duration. For patients dropping out of the lifestyle intervention, further course will be determined by the responsible physician.

8.3 Economic evaluation

The aim of the economic evaluation is to compare the costs and health effects of a lifestyle program versus "usual care". The analysis will be a cost-effectiveness analysis with the proportion of healthy singleton of more than 37 week gestation born after vaginal delivery as the primary outcome.
The economic evaluation will be performed from a societal perspective. Direct medical and non-medical costs (intervention costs, time and travel costs) as well as indirect non-medical costs (productivity losses) will be taken into account.

We will build on the work of the ZonMW funded "Paraplu" study, in which cost data data of six cost-effectiveness studies were integrated. Dr H. Groen played an important role in this process.

The time horizon will be from randomisation to the end of follow-up. Resource use will be recorded as individual patient data in the CRF. In addition each woman will receive a questionnaire for details on associated direct costs of professional care and on indirect costs like transportation, sporting activity and productivity loss. These questionnaires will be handed out in consultation 2, week 3 of the lifestyle module or at the start of fertility treatment for women that were randomised for "usual care". This will be repeated at the time points of the other questionnaires (12, 24 and 52 weeks after randomisation and at the end of follow-up).
Resource use will be valued according to Dutch guidelines. Intervention costs will determined based on actual resource use obtained from the centers. Costs will reflect the following

resources: staff, materials, equipment, housing, overhead. Productivity loss will be valued by the friction cost method according to Dutch guidelines (Oostenbrink et al. 2004). Outcome of pregnancy will be recorded and valued using tariffs or data from the literature (Lukassen et al, 2004).

Detailed information on maternal complication will be obtained from obstetricians treating the woman concerned using patient medical files. Six weeks after the expected day of delivery all women will be contacted by telephone to ask information on the delivery and on the health of the child. If the child has been hospitalised the paediatrician treating the child will be contacted for further information.

Scenario analysis will be performed to model cost-effectiveness beyond the time horizon of the study. For longer term analyses, costs and effects will be discounted at commonly accepted rates. Sensitivity analysis will be performed on costs, pregnancy rates of the two groups and the valuation of different outcomes (no child, handicapped child, twin, healthy child, obstetric complications). Uncertainty surrounding cost-effectiveness estimates will be explored by bootstrapping. Cost will be presented in euros.

9. ETHICAL CONSIDERATIONS

9.1 Regulation statement

The study will be conducted according to the principles of the Declaration of Helsinki (Adopted by the 18th WMA General Assembly, Helsinki, Finland, June 1964, and amended by the:
29th WMA General Assembly, Tokyo, Japan, October 1975
35th WMA General Assembly, Venice, Italy, October 1983
41st WMA General Assembly, Hong Kong, September 1989
48th WMA General Assembly, Somerset West, Republic of South Africa, October 1996
and the 52nd WMA General Assembly, Edinburgh, Scotland, October 2000
Note of Clarification on Paragraph 29 added by the WMA General Assembly, Washington 2002
Note of Clarification on Paragraph 30 added by the WMA General Assembly, Tokyo 2004
and in accordance with the Medical Research Involving Human Subjects Act (WMO)

9.2 Recruitment and consent

Women eligible for the study will de referred to a research nurse by their treating doctors. This person is dedicated to counselling eligible patients, randomisation and follow-up for data, but not to the experimental intervention. We will collaborate with the research nurses who are involved in the obstetric and urogynaecological consortium. In this consortium, eight large randomised clinical trials are performed. Information on this infrastructure is obtainable from http://www.studies-obsgyn.nl/
The research nurse must explain to each subject the nature of this study, its purpose, procedures, expected duration and the potential risks and benefits involved in study participation along with any discomfort it may entail. Each subject must be informed that

participation in the study is voluntary and that withdrawal of consent will not affect his/her right to the most appropriate medical treatment or affect the doctor relationship.
This informed consent will be given by means of a standard written statement. It will be written so as to be easily understood by the subject. The subject will be given the time to read and understand the statement herself before signing her consent and dating the document. The subject will receive a copy of the written statement once signed.
The informed consent form must be considered as a part of the protocol with which it is to be submitted by the investigator to the IRB/Ethics committee.
Patients will be given a maximum of one week to consider their decision to participate in the study.

9.3 Objection by minors or incapacitated subjects (if applicable)

Incapacitated adults will be not asked to participating in the study. Minors are excluded as well.

9.4 Benefits and risks assessment, group relatedness

The ethical issues pertaining to this project will be discussed by considering the four ethical principles as discussed by Gillon. (37)

Autonomy: After the intake consultation and counseling session by the research nurse, the patient is given the choice to participate in the study. This process of informed consent respects her autonomy.

Beneficence: The advantages of a weight loss and exercise program in overweight or obese subfertile women (in order to improving chances of conception and eventually taking a baby home) have clearly been shown. Even if the patient does not conceive during the 6 months period, the health benefits of weight loss on the long-term are clearly in the interest of the patient.

Non-maleficence: In some reproductive medicine units, overweight or obese subfertile women are counselled and advised to loose weight before treatment is started. By starting this project, fertility treatment in the intervention arm will be delayed for a maximum of 6 months. There is no evidence that this short delay will influence the eventual pregnancy chances. The structured weight loss and exercise program will probably lead to higher spontaneous and therapy induced ongoing pregnancy chances. Patients with overweight or obesity have additional risks during pregnancy. The risks involve hypertension (OR 2.2), preeclampsia (OR 1.8-2.7) pregnancy diabetes (OR 2.2-3.4), ceasarean section (OR 1.3-2.4) compared to normal weight women (see appendix table of evidence). These risks are not additionally influenced by fertility treatment. Lifestyle intervention aims to reduce the

involved pregnancy risks and is part of the analysis of this study. The lifestyle intervention perse does not involve additional risk to the patients, but reduces the risks of longterm health complications.

Before giving informed consent all eligible women are informed about the risks mentioned before (in writing in the patients' brochure). Women who are considered to be at the highest risk (i.e. those who have pre-existent hypertension or diabetes, or pregnancy induced hypertension, (pre)eclampsia or HELLP syndrome in a previous pregnancy) are excluded from participation. During the study adverse events will be monitored by a SMB.

Justice: Introducing the study will not infringe on other's rights (rights based justice). The study will mainly be financed through ZonMW and therefore no conflict distribution of scarce funds (distributive justice). Inclusion in the study respects all morally acceptable laws (legal justice).

Compensation for injury
Ingevolge artikel 7 van de Wet op medisch wetenschappelijk onderzoek met mensen (Stlb. 1998, 161) is voor de deelnemende proefpersonen een verzekering afgesloten die de door het onderzoek veroorzaakte schade door dood of letsel van de deelnemende proefpersonen dekt. Deze verzekering voldoet aan de bepalingen van het Besluit Verplichte verzekering bij medisch-wetenschappelijk onderzoek met mensen (Stlb. 2003, 266).
Aan het onderzoek deelnemende proefpersonen zullen schriftelijk worden ingelicht over deze verzekering.
Elke aan het onderzoek participerende instelling draagt zorg voor de verzekering van de in de eigen instelling te includeren proefpersonen.
Elk deelnemend centrum heeft ook een aansprakelijkheidsverzekering als bedoeld in art. 7 WMO.

10. ADMINISTRATIVE ASPECTS AND PUBLICATION

10.1 Handling and storage of data and documents

Data will be handled confidentially and anonymously. To be able to trace data to an individual subject, a subject identification code list will be used. Participating subjects will be registered by a 5-digit number that will be used to link the data to the subject. This personal code will be on all forms retrieved from participants. The key to the code is safeguarded by the investigator. The handling of personal data will comply with the Dutch Personal Data Protection Act (in Dutch: De Wet Bescherming Persoonsgegevens, Wbp).
Serum and cordblood will be stored for 5 years at minus 80 degrees anonymously and coded as mentioned above.

10.2 Amendments

Amendments are changes made to the research after a favourable opinion by the accredited METC has been given. All amendments will be notified to the METC that gave a favourable opinion.

10.3 Annual progress report

The sponsor/investigator will submit a summary of the progress of the trial to the accredited METC once a year. Information will be provided on the date of inclusion of the first subject, numbers of subjects included and numbers of subjects that have completed the trial, serious adverse events/ serious adverse reactions, other problems, and amendments.

10.4 End of study report

The investigator will notify the accredited METC of the end of the study within a period of 8 weeks. The end of the study is defined as the last patient's last visit.
In case the study is ended prematurely, the investigator will notify the accredited METC, including the reasons for the premature termination.

Within one year after the end of the study, the investigator/sponsor will submit a final study report with the results of the study, including any publications/abstracts of the study, to the accredited METC.

11. REFERENCES

Al-Azemi M, Omu FE, Omu AE.The effect of obesity on the outcome of infertility management in women with polycystic ovary syndrome.Arch Gynecol Obstet. 2004 Dec;270(4):205-10.

Amer SA, Li TC, Ledger WL.Ovulation induction using laparoscopic ovarian drilling in women with polycystic ovarian syndrome: predictors of success.Hum Reprod. 2004 Aug;19(8):1719-24.

Avenell A, Broom J, Brown TJ, Poobalan A, Aucott L, Stearns SC, Smith WC, Jung RT, Campbell MK, Grant AM. Systematic review of the long-term effects and economic consequences of treatments for obesity and implications for health improvement.
Health Technol Assess. 2004 May;8(21):iii-iv, 1-182. The British Dietetic Association Ltd. J Hum Nutr Diet 2004; 17: 317-35.

Balen AH, Anderson RA. Impact of Obesity on female reproductive health: British Fertility Society, Policy and Practice Guidelines. Hum Fertil (Camb). 2007 Dec;10(4):195-206.

Balen AH, Dresner M, Scott EM, Drife JO. Should obese women with polycystic ovary syndrome receive treatment for infertility? BMJ 2006;332:434-5.

Bemelmans WJE, et al. Interventies ter preventie van overgewicht in de wijk, op school, op het werk en in de zorg. Een verkennende studie naar de effecten. RIVM report 260301005, Bilthoven, 2004.

Bemelmans WJE, et al. Leefstijladvisering door praktijkondersteuner bij mensen met overgewicht : opzet , procesevaluatie en eerste resultaten van de Groningse Overweight and Lifestyle (GOAL) study. RIVM Rapport

Bemelmans W, van Baal P, Wendel-Vos W, Schuit J, Feskens E, Ament A, Hoogenveen R. The costs, effects and cost-effectiveness of counteracting overweight on a population level. A scientific base for policy targets for the Dutch national plan for action. Prev Med. 2008 Feb;46(2):127-32. Epub 2007 Aug 3.

Cnattingius S, Bergström R, Lipworth L, Kramer MS. Prepregnancy weight and the risk of adverse pregnancy outcomes. N Engl J Med 1998;338(3):147-52.

Bogers RP, Vijgen SMC, Bemelmans WJE.Costs of lifestyle interventions within health care and the amount of weight loss achieved. RIVM rapport 260701002/2006, Bilthoven 2006.

Bolúmar F, Olsen J, Rebagliato M, Sáez-Lloret I, Bisanti L. Body mass index and delayed conception: a European Multicenter Study on Infertility and Subfecundity. Am J Epidemiol. 2000 Jun 1;151(11):1072-9.

Bussen S, Sütterlin M, Steck T.Endocrine abnormalities during the follicular phase in women with recurrent spontaneous abortion. Hum Reprod 1999;14:18-20.

CBO guidline Diagnostiek en behandeling van obesitas bij volwassenen en kinderen. www.cbo.nl 2007

Clark AM, Ledger W, Galletly C, Tomlinson L, Blaney F, Wang X l. Weight loss results in significant improvement in pregnancy and ovulation rates in anovulatory obese women. Hum Reprod 1995;10:2705-12.

Clark AM Thornley B, Tomlinson L, Galletley C, Norman RJ . Weight loss in obese infertile women results in improvement in reproductive outcome for all forms of fertility treatment. Hum Reprod 1998;13:1502-05.

Cnattingius S, Taube Al. Stillbirth and rate of fetal death in 76,761 postterm pregnancies in Sweden 1991; a register study. Acta Obstet Gynecol Scand 1998;77:582

Clinical Guidelines on the Identification, Evaluation, and Treatment of Overweight and Obesity in Adults--The Evidence Report. National Institutes of Health. Obes Res 1998;6(S)2:51-209.

Custers IM, Steures P, van der Steeg JW, van Dessel TJ, Bernardus RE, Bourdrez P, Koks CA, Riedijk WJ, Burggraaff JM, van der Veen F, Mol BW. External validation of a prediction model for an ongoing pregnancy after intrauterine insemination. Fertil Steril. 2007 Aug;88(2):425-31..

Dechaud H, Anahory T, Reyftmann L, Loup V, Hamamah S, Hedon B. Obesity does not adversely affect results in patients who are undergoing in vitro fertilization and embryo transfer. Eur J Obstet Gynecol Reprod Biol. 2006 Jul;127(1):88-93.

Crosignani PG, Colombo M, Vegetti W, Somigliana E, Gessati A, Ragni G. Overweight and obese anovulatory patients with polycystic ovaries: parallel improvements in anthropometric indices, ovarian physiology and fertility rate induced by diet. Hum Reprod 2003 Sep;18(9):1928-32.

Dixon JB, Dixon ME, and O'Brien PE. Birth outcomes in obese women after laparoscopic adjustable gastric banding. Obstet Gynecol 2005;106:965-72.

Dodson WC, Kunselman AR, Legro RS. Association of obesity with treatment outcomes in ovulatory infertile women undergoing superovulation and intrauterine insemination. Fertil Steril. 2006 Sep;86(3):642-6..

Dokras A, Jagasia DH, Maifeld M, Sinkey CA, VanVoorhis BJ, Haynes WG.Obesity and insulin resistance but not hyperandrogenism mediates vascular dysfunction in women with polycystic ovary syndrome. Fertil Steril. 2006 Dec;86(6):1702-9.

Dunaif A. Insulin resistance and the polycystic ovary syndrome: Mechanism and implications for pathogenesis. Endocr.Rev. 1997;18:774-800.

Edwards LE, Hellerstedt WL, Alton IR, Story M, Himes JH. Pregnancy complications and birth outcomes in obese and normal-weight women: effects of gestational weight change. Obstet Gynecol. 1996 Mar;87(3):389-94.

Fedorcsák P, Storeng R, Dale PO, Tanbo T, Abyholm T. Obesity is a risk factor for early pregnancy loss after IVF or ICSI. Acta Obstet Gynecol Scand 2000;79:43-8.

Fedorcsák P, Dale PO, Storeng R, Ertzeid G, Bjercke S, Oldereid N, Omland AK, Abyholm T, Tanbo T.Impact of overweight and underweight on assisted reproduction treatment. Hum Reprod 2004;19:2523-8.

Franz MJ, VanWormer JJ, Crain L, et al. Weight loss outcomes: a systematic review and meta-analysis of weight loss clinical trials with a minimum 1-year follow-up. J Am Diet Assoc 2007; 107:1755-1767.

Garbaciak JA Jr, Richter M, Miller S, Barton JJ. Maternal weight and pregnancy complications. Am J Obstet Gynecol 1985;152(2):238-45.

Glazer NL, Hendrickson AF, Schellenbaum GD, and Mueller BA. Weight change and the risk of gestational diabetes in obese women. Epidemiology 2004;15:733-7.

Gillon, R. (1994). Medical ethics: four principles plus attention to scope. BMJ. 309, 184-188

Guzick DS, Wing R, Smith D, Berga SL, Winters SJ. Endocrine consequences of weight loss in obese, hyperandrogenic, anovulatory women. Fertil Steril 1994 Apr;61(4):598-604.

Herman WH, Hoerger TJ, Brandle M, et al. The cost-effectiveness of lifestyle modification or metformin in preventing type 2 diabetes in adults with impaired glucose tolerance. Ann Intern Med 2005; 142(5):323-32.

Hoeger KM, et al. A randomized, 48-week, placebo ontrolled trial of intensive lifestyle modification and/or metformin therapy in overweight women with polycystic ovary syndrome: a pilot study. Fertil Steril 2004;82:421-9.

Hollmann M, Runnebaum B, Gerhard I. Effects of weight loss on the hormonal profile in obese, infertile women. Hum Reprod 1996 Sep;11(9):1884-91.

Huber-Buchholz MM, Carey DG, Norman RJ. Restoration of reproductive potential by lifestyle modification in obese polycystic ovary syndrome: role of insulin sensitivity and luteinizing hormone. J Clin Endocrinol Metab 1999;84:1470-4.

Hunault CC, Laven JS, van Rooij IA, Eijkemans MJ, te Velde ER, Habbema JD. Prospective validation of two models predicting pregnancy leading to live birth among untreated subfertile couples.Hum Reprod. 2005 Jun;20(6):1636-41.

Knowler WC et al. Reduction in the incidence of type 2 diabetes with lifestyle intervention or metformin. N Engl J Med 2002;346:393-403.

Imani B, Eijkemans MJ, te Velde ER, Habbema JD, Fauser BC. A nomogram to predict the probability of live birth after clomiphene citrate induction of ovulation in normogonadotropic oligoamenorrheic infertility. Fertil Steril. 2002 Jan;77(1):91-7.

Kiddy DS, Hamilton-Fairley D, Bush A, Short F, Anyaoku V, Reed MJ, et al. Improvement in endocrine and ovarian function during dietary treatment of obese women with polycystic ovary syndrome. Clin Endocrinol (Oxf) 1992 Jan;36(1):105-11.

Knowler WC, Barrett-Connor E, Fowler SE, Hamman RF, Lachin JM, Walker EA, Nathan DM; Diabetes Prevention Program Research Group.Reduction in the incidence of type 2 diabetes with lifestyle intervention or metformin. N Engl J Med. 2002 Feb 7;346(6):393-403.

Kristensen J, Vestergaard M, Wisborg K, Kesmodel U, Secher NJ. Pre-pregnancy weight and the risk of stillbirth and neonatal death. Pregnancy weight and the risk of stillbirth and neonatal death. BJOG 2005;112:403-8.

Lashen H, Lashen H, Ledger W, Bernal AL, Barlow D.Extremes of body mass do not adversely affect the outcome of superovulation and in-vitro fertilization.
Hum Reprod. 1999 Mar;14(3):712-5

Linne Y . 2004 Effects of obesity on women ‘s reproduction and complications during pregnancy. Obesity reviews 2004;5:137-43.

Lintsen AM, Pasker-de Jong PC, de Boer EJ, Burger CW, Jansen CA, Braat DD. Effects of subfertility cause, smoking and body weight on the success rate of IVF. Hum Reprod 2005;20:1867-75.

Lukassen HG, Schönbeck Y, Adang EM, Braat DD, Zielhuis GA, Kremer JA.
Cost analysis of singleton versus twin pregnancies after in vitro fertilization.
Fertil Steril. 2004 May;81(5):1240-6.

Maheshwari A, Stofberg L, Bhattacharya S. Effect of overweight and obesity on assisted reproductive technology –a systematic review. Human Reproduction Update update Hum Reprod Update. 2007 Sep-Oct;13(5):433-44.

McTigue KM, Harris R, Hemphill B, Lux L, Sutton S, Bunton AJ, Lohr KN.Screening and interventions for obesity in adults: summary of the evidence for the U.S. Preventive Services Task Force.Ann Intern Med. 2003 Dec 2;139(11):933-49

Moran LJ, Noakes M, Clifton PM, Tomlinson L, Galletly C, Norman RJ. Dietary composition in restoring reproductive and metabolic physiology in overweight women with polycystic ovary syndrome. J Clin Endocrinol Metab 2003 Feb;88(2):812-9.

Mulders AG, Eijkemans MJ, Imani B, Fauser BC.Prediction of chances for success or complications in gonadotrophin ovulation induction in normogonadotrophic anovulatory infertility. Reprod Biomed Online. 2003 Sep;7(2):170-8.

Mulders AG, Laven JS, Eijkemans MJ, Hughes EG, Fauser BC.Patient predictors for outcome of gonadotrophin ovulation induction in women with normogonadotrophic anovulatory infertility: a meta-analysis.Hum Reprod Update. 2003 Sep-Oct;9(5):429-49.

National Institute for Clinical Excellence. Fertility assessment and treatment for people withfertility problems. A clinical guideline. London: RCOG Press, 2004

Nichols JE, Crane MM, Higdon HL, Miller PB, Boone WR. Extremes of body mass index reduce in vitro fertilization pregnancy rates.Fertil Steril. 2003 Mar;79(3):645-7.

Norman RJ, Clark AM. Obesity and reproductive disorders, a review. Reprod Fertil Dev 1998;10:55-63.

Norman RJ, Noakes M, Wu R, Davies MJ, Moran L, Wang JX. Improving reproductive performance in overweight/obese women with effective weight management. Hum Reprod Update 2004;10:267-80.

Oers van JAM. Gezondheid op koers? Volksgezondheid Toekomst Verkenning 2002. Rijksinstituut voor Vokksgezondheid en Milieu, rapport nr. 270551001, Bilthoven 2002.

Oostenbrink JB, Koopmanschap MA, Rutten FF. Standardisation of costs: the Dutch Manual for Costing in economic evaluations. Pharmacoeconomics. 2002;20(7):443-54.

Oostenbrink JB Manual for costing : methods and standard costs for economic evaluation in helath care (In Dutch). Amstelveen, The Netherlands: Health Insurance Council ; Update version 2004.

Palomba S, Giallauria F, Falbo A, Russo T, Oppedisano R, Tolino A, et al. Structured exercise training programme versus hypocaloric hyperproteic diet in obese polycystic ovary syndrome patients with anovulatory infertility: a 24-week pilot study. Hum Reprod 2007 Dec 23.

Pasquali R, Pasquali R, Antenucci D, Casimirri F, Venturoli S, Paradisi R, Fabbri R, Balestra V, Melchionda N, Barbara L.Clinical and hormonal characteristics of obese amenorrheic hyperandrogenic women before and after weight loss.J Clin Endocrinol Metab. 1989 Jan;68(1):173-9.

Powell K, Pratt M.. Physical activity and health. BMJ 1996;313:126-7.

Ramlau-Hansen CH Thulstrup AM, Nohr EA, Bonde JP, Sorensen TIA, Olsen J. Subfecundity in overweight and obese couples. Human Reprod. 2007;22:1634-7

Sebire NJ, Jolly M, Harris JP, Wadsworth J, Joffe M, Beard RW, Regan L, Robinson S.Maternal obesity and pregnancy outcome: a study of 287,213 pregnancies in London. Int J Obes Relat Metab Disord. 2001 Aug;25(8):1175

Stamets K, Taylor DS, Kunselman A, Demers LM, Pelkman CL, Legro RS. A randomized trial of the effects of two types of short-term hypocaloric diets on weight loss in women with polycystic ovary syndrome. Fertil Steril 2004;81:630-7.

Tang T, Glanville J, Hayden CJ, White D, Barth JH, Balen AH. Combined lifestyle modification and metformin in obese patients with polycystic ovary syndrome. A randomized placebo-controlled, doube-blind multicentre study. Human Reprod 2006;21:80-9.

Usha Kiran TS, Hemmadi S, Bethel J, Evans J. Outcome of pregnancy in a woman with an increased body mass index.. BJOG 2005;112:768-72

Van der Steeg, Steures P, Eijkmans MJC, Habbema JDH, Hompes PGA, Broekmans FJ, van Dessel HJHM, Bossuyt PMM, van der Veen F, Mol BWJ. Pregnancy prediction is predictable: a large scale prospective external validation of prediction of spontaneous pregnancy in subfertile couples. Human Reprod. 2007;22;536-542.

van der Steeg JW JW, Steures P, Eijkemans MJ, Habbema JD, Hompes PG, Burggraaff JM, Oosterhuis GJ, Bossuyt PM, van der Veen F, Mol BW. Obesity affects spontaneous pregnancy chances in subfertile, ovulatory women.Hum Reprod. 2008 Feb;23(2):324-8.

Van Strien T, Rookus MA, Bergers GP, Frijters JE, Defares PB.Life events, emotional eating and change in body mass index. International Journal of Eating Disorders,5, 747-755. Life events, emotional eating and change in body mass index.Int J Obes. 1986;10(1):29-35

Vijgen SMC, Hoogendoorn M, Baan CA, de Wit GA, Limburg W, Feenstra TL. Cost effectiveness of preventive interventions in Type 2 Diabetes Mellitus. A systematic literature review. Pharmacoeconomics 2006; 24(5): 425-441.

Waller DK, Mills JL, Simpson JL, Cunningham GC, Conley MR, Lassman MR, and Rhoads GG. Are obese women at higher risk for producing malformed offspring? Am J Obstet Gynecol 1994;170:541-548.

Wang JX, Davis MJ, Norman RJ . Body mass and probability of pregnancy during assisted reproduction treatment. Brit Med J 2000; 321:1320-1.

Wang JX, Davis MJ, Norman RJ. Polycystic ovarian syndrome and the risk of spontaneous abortion following assisted reproductive technology treatment. Hum Reprod 2001;16:2606-9.

Wang JX, et al. Obesity increases the risk of spontaneous abortion during infertility treatment. Obes Res 2002;10:551-4.

Weiss JL, Malone FD, Emig D, Ball RH, Nyberg DA, Comstock CH, Saade G, Eddleman K, Carter SM, Craigo SD, Carr SR, D'Alton ME; FASTER Research Consortium
Obesity, obstetric complications and cesarean delivery rate--a population-based screening study. Am J Obstet Gynecol. 2004 Apr;190(4):1091-7.

Werler MM, Louik C, Shapiro S, and Mitchell AA. Prepregnant weight in relation to risk of neural tube defects. JAMA 1996,275:1089-92.

Wittemer C, Ohl J, Bailly M, Bettahar-Lebugle K, Nisand I.Does body mass index of infertile women have an impact on IVF procedure and outcome?J Assist Reprod Genet. 2000 Nov;17(10):547-52

WHO document ‘Ministerial conference on counteracting obesity’.
WW.euro.who.int/document/E90143.pdf

Zelissen PM, Mathus-Vliegen EM. Treatment of overweight and obesity in adults: proposal for a guideline]Ned Tijdschr Geneeskd. 2004;16;148(42):2060-6 .

Summary of studies assessing outcome after lifestyle intervention

**Anovulatory patients**							
**article**	**subjects**	**Intervention kCal/day**	**duration in weeks**	**inclusion BMI**	**ovulation**	**Pregnancy**	**resumption regular cycles**
Kiddy 1992(1)	13	1000 kcal/day	26-30 weeks	>25		5	9 of 11 with >5% weight loss resumed regular menses
Guzick 1994(2)	RCT<br>6 diet<br>6 waiting list	8 weeks 400kcal/day then<br>4 weeks "refeeding"	12 weeks	Mean BMI 40,6	4 in diet<br>0 in waiting list		
Clark 1995(3)	13	lifestyle program	26 weeks	≥ 30	12	5	
Hollmann 1996(4)	35	5000-10000 cal/ week reduction	32 (+/- 14 weeks)	>30	Not assessed	10	28
Huber-Buchholz 1999(5)	18<br>3 drop-outs	Diet and exercise program	24	>27	9	2	
Hoeger 2004 (6)	11 lifestyle only	500-1000 kcal/day deficit	24	>25	OR 8,97 at > 3% weight loss	2	
Tang 2006(7)	74<br>6 drop-outs	Dietary advice with<br>500 kcal reduction/day	24	>30		4	58% improvement in menses
Palomba 2007(8)	20	800 kcal deficit/day	24	30 - 35	5	2	

**Anovulatory(ov-) and ovulatory patients(ov+)**							
**article**	**subjects**	**intervention Kcal/day**	**duration in weeks**	**inclusion BMI**	**ovulation**	**pregnancy**	**resumption regular cycles**
Clark 1998(9)	87<br>20 drop-outs	lifestyle program	24	>35	60 of 67	-18 spontaneous<br>-34 after fertility treatment	
Crosignani 2003 (10)	33 -<br>27 ov-<br>6 ov+	1200 kcal/day	28	>25	15 of 27ov-	10	18 of 27ov-
Moran 2003(11)	45<br>14 drop-outs	1500 kcal/day	12	>35		3	11of 25 ov-

**Pre-pregnancy weight loss and pregnancy outcome**			
**article**	**subjects**	**intervention**	**outcome**
Glazer 2004(12)	702 with weight 200-249 lbs	Weight loss	Weight loss of > 10 lbs RR 0,5(95% CI 0.28-0.92) of developing gestational diabetes in subsequent pregnancy
Dixon 2005 (13)	79 –pre-operative weight 125kg ±20	Laparoscopic gastric bypass	After an average weight loss of 28kg - significant decrease in gestational diabetes, PIH and pre-eclampsia compared to matched obese cohort

Amendments made to the study protocol

Protocol version 2 was approved by the Medical Ethics Committee (MEC) of the University Medical Center Groningen at 26-2-2009. The first patient was included and randomized at 9-6-2009. Participating clinics were University Medical Center Groningen, Academic Medical Center, VU Medical Center, Leids University Medical Center, Erasmus Medical Center, Maastricht University Medical Center, University Medical Center Utrecht, Radboud University Medical Center Nijmegen, Deventer Hospital, Rijnstate Hospital, Maximal Medical Center, Onze Lieve Vrouwe Gasthuis, Medical Spectrum Twente, Atrium Medical Center, Isala Clinics, Medical Center Alkmaar, Martini Hospital, Meander Medical Center and Leveste Hospital.

In protocol version 3 the following changes in the study protocol were made (after including 86 patients). The MEC approved version 3 of the protocol at 2-6-2010.

1. Kaplan-Meier survival analyses instead of Chi² analyses will be used for statistical analyses. Log-rank tests or Cox analysis can reveal differences in the two study arms.
2. Number of oocytes, embryos and cryo preserved embryos after IVF-ICSI treatment were added as study endpoints.
3. Apgar score, arterial pH and congenital anomalies were added as study endpoints.
4. The upper limit of age was changed from 38 years to 39 years.
5. Women with moderate hypertension (systolic tension 140-159 mmHg and diastolic tension 90-109 mmHg) became eligible if there hypertension was treated.
6. Couples who use donor semen were not eligible.
7. Baseline measurements will be taken by the nurse who randomizes the patient and not by the nurse who provides the lifestyle intervention.
8. We added a questionnaire of weight gain during pregnancy
9. We advised all care providers in case of pregnancy to request an oral glucose tolerance test.
10. Orbis Medical Center, Laurentius Hospital, st. Antonius Hospital, Medical Center Leeuwarden, ZGT Almelo and Jeroen Bosch Hospital were added as participating hospitals.
11. The Radboud UMCN withdrew from study participation. They did not include any patients.

In protocol version 4 (approved at 21-10-10), after inclusion of 188 patients, the following changes in the study protocol were made:

1. Women with a BMI of 40 kg/m2 or above became eligible for study participation.
2. Number of instrumental deliveries, implementation chance per embryo transfer during the IVF/ICSI procedure were added as study endpoints.
3. We started with collection of cord blood after delivery.
4. The Erasmus MC withdrew from study participation. They did not include any patients.

In protocol version 5, after inclusion of all 577 patients (follow-up was still ongoing), the following changes were made (approved at 13-11-2013).

1. Kaplan-Meier analysis will be used for the secondary endpoint ‘ongoing pregnancies’.
2. Complications of fertility treatment were added as study endpoints.
3. We added education and ethnicity to our possible confounding factors.
4. We asked for permission to use the data of this study also in the study of the follow-up of the children born in our trial.

Costs and effects of a structured lifestyle program in overweight and obese subfertile couples to prevent unnecessary treatment and improve reproductive outcome

STATISTICAL ANALYSIS PLAN

Authors: Henk Groen, epidemiologist
Meike Mutsaerts, PhD candidate
Anne van Oers, PhD candidate

Date of document: December 20, 2013

TABLE OF CONTENTS

1. AUTHORIZATION OF STATISTICAL ANALYSIS PLAN 3
2. LIST OF ABBREVIATIONS AND DEFINITION OF TERMS 4
3. INTRODUCTION 5
4. PROTOCOL SUMMARY 6
4.1 Study background and rationale 6
4.2 Study design 6
4.3 Study flow chart 7
5. STUDY OBJECTIVES 8
5.1 Endpoints 8
5.1.1 General considerations 8
5.1.2 Primary efficacy endpoint 8
5.1.3 Secondary efficacy endpoints 8
6. STATISTICAL METHODS AND REPORTING 10
6.1 Sample size 10
6.2 Level of significance 10
6.3 Randomization, database lock. 10
6.4 Study analysis 10
6.5 Study participants characteristics 11
6.6 Efficacy parameters 11
6.7 Lifestyle program effectiveness 13
7. REFERENCES 14

1. AUTHORIZATION OF STATISTICAL ANALYSIS PLAN

Title: "Lifestyle study in subfertile women"

Study code: NA

EudraCT: NA

Author: Henk Groen, MD PhD, epidemiologist

2. LIST OF ABBREVIATIONS AND DEFINITION OF TERMS

SAP	Statistical Analysis Plan
TCC	Trial Coordination Center

3. INTRODUCTION

The purpose of the Statistical Analysis Plan (SAP) is to provide a comprehensive and detailed description of the rationale, methods and presentation of the data analysis for the LIFEstyle study. In this study, efficacy, cost-effectiveness and safety of fertility treatment with and without a lifestyle intervention will be assessed in subfertile obese women.

4. PROTOCOL SUMMARY

4.1 Study background and rationale

Subfertility affects approximately one in ten couples planning conception. Among subfertile women, about 30% are overweight or even obese. Epidemiological data suggest that the reduction of overweight will increase the chances of conception, decrease pregnancy complications and improve perinatal outcome. In small intervention studies beneficial reproductive effects of improvement of lifestyle leading to a reduction in body weight have been demonstrated. The British Fertility Society advises that fertility treatment be withheld if the body mass index (BMI) is over 35.

In the Netherlands, there is at present no agreed standard of care for subfertile women with overweight or obesity. Some centers simply withhold treatment of couples in whom the woman is overweight or obese, but most fertility centers treat overweight or obese women irrespective of their BMI. In a few centers, support is offered to women to help them lose weight.

In view of this lack of evidence and strong practice variation we propose a randomized clinical trial in overweight and obese subfertile women, in which we compare the costs and effects of a six months structured lifestyle program, aimed at weight loss, to “usual care”. The intervention aims to prevent unnecessary fertility treatment and prevent complications associated with fertility treatment and obesity-related pregnancy complications, thus improving pregnancy chances and perinatal outcome.

4.2 Study design

The study is a multicenter randomized clinical trial. Subfertile women (age 18-39) with a BMI of 29 kg/m² or above will be included. Exclusion criteria are: azoospermia or donor semen, severe endometriosis, chronic anovulation due to WHO III and endocrinopathies. Women with an untreated pre-existent hypertension or diabetes type 1, or pregnancy-induced hypertension, preeclampsia, eclampsia or HELLP syndrome in a previous pregnancy are excluded.

The intervention consists of a structured lifestyle program of six months, which aims at realistic weight loss of at least 5% to 10%, achieved by the combination of a healthy diet (caloric reduction of 600kCal/day), increase of physical activity (aiming at 10.000 steps per day) and two to three times a week sporting activity, and behavioral modification. The structured lifestyle program, in which practice variation is minimized using a structured software program, has been developed, used and evaluated previously (Zon-MW project 50-

50110-98-078; the Groninger Overweight And Lifestyle study (GOAL)). After six months of lifestyle intervention patients will start fertility treatment as indicated.

In the “usual care” arm fertility treatment will be started if this is justified by the individual prognosis (guideline NVOG)

All randomized women will fill in questionnaires concerning: diet, eating behavior, physical activity and smoking: SF-36, FFQ, DEBQ, Squash list, at inclusion, and at 12 weeks, 6 months, 12 months and 24 months after randomization. Blood samples (10 cc) will be taken at the start, 3 months and 6 months after the start of the study.

4.3 Study flow chart

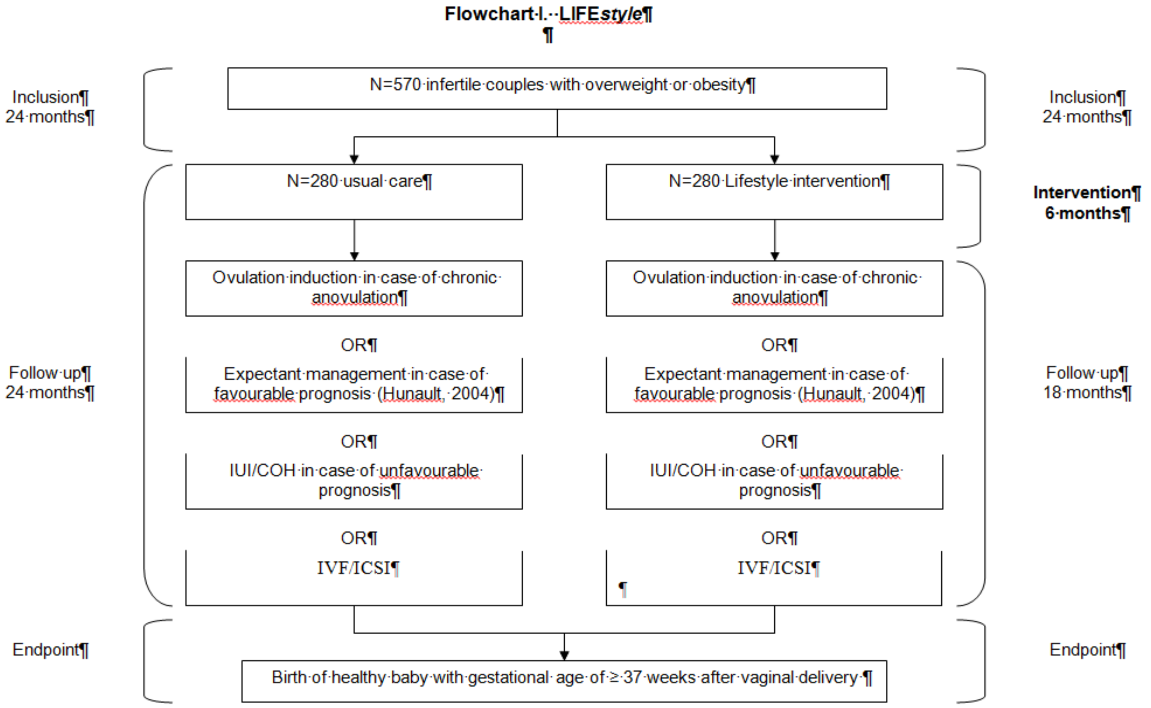

5. STUDY OBJECTIVES

The objective of the study is to compare the costs and effects of a six months structured lifestyle program, aimed at weight loss, to “usual care” with the aim to prevent obesity-related pregnancy complications, unnecessary fertility treatment and complications associated with fertility treatment, enhance pregnancy chances and improve perinatal outcome. The control group of women will receive fertility treatment “care as usual” without lifestyle intervention.

5.1 Endpoints

5.1.1 General considerations

We aim to estimate the costs and effects of a structured lifestyle program in terms of costs per additional healthy singleton birth rate beyond 37 weeks gestation born after vaginal delivery in a follow-up period of 24 months.

5.1.2 Primary efficacy endpoint

The primary endpoint will be a healthy singleton of more than 37 week gestation born after vaginal delivery. The initial analysis will be done by intention-to-treat, so we will not make a difference for treatment-independent pregnancies and pregnancies that occurred after treatment. Patients who drop out of the study will be analyzed according to their group of randomization. In a per-protocol analysis drop-outs from the lifestyle intervention will be analyzed as non-compliant. For the conventional treatment no difference will be made between the per-protocol and the intention-to-treat analysis.

5.1.3 Secondary efficacy endpoints

Secondary outcome parameters are:

1. Pregnancy outcome and -complications: miscarriage, gestational diabetes, Pregnancy induced hypertension, preeclampsia, HELLP, prematurity<37 weeks, macrosomia (>p90 (Large for Gestational Age), induction of labour, prolonged duration of labour, operative delivery, assisted delivery and increased blood loss, and multiple pregnancies
2. Percentage of women needing fertility treatment in both groups (OI, IUI, IVF, ICSI) and ongoing pregnancy rates in these treatments.

3. Quality and number of oocytes and embryos and cryo embryos resulting from an IVF or ICSI procedure and the amount of embryos per transfer and implantation rate of transferred embryos
4. Complications during fertility treatment: ovarian hyperstimulation syndrome, infection, tubal pregnancies, torsion (etc).
5. Perinatal outcome: stillbirth, pregnancy duration, body weight, Apgar scores, arterial pH, congenital anomalies and neonatal admission to a neonatal medium, high or intensive care unit.
6. Quality of life, pre-pregnancy body weight, weight gain during pregnancy, waist circumference, behaviour influencing weight, i.e. nutritional habits and exercise pattern, blood pressure, glucose/insulin ratio (HOMA), hormonal profile (androgens, adipokines and cytokines) and costs.

6. STATISTICAL METHODS AND REPORTING

6.1 Sample size

On the basis of the literature the cumulative live birth rate in a follow-up of two years of subfertility treatment will be 45% for the “usual care” group. The lifestyle intervention group is estimated to have a live birth rate of 60%. We expect an improvement of healthy singleton of more than 37 week gestation born after vaginal delivery from 45% to 60% in the intervention group.[1,2,3] Drop-out is expected to be 20% in the intervention arm [4] and is included in the analysis of effectiveness. To prove that, we need to include two groups of 272 women to be included (alpha 0.05, power 80%). To account for 5% lost to follow up 285 women will be included per group. In total, 570 women will be included.

6.2 Level of significance

For all hypothesis testing performed in the Lifestyle study, a p-value of less than 0.05 will be considered to indicate statistical significance.

6.3 Randomization, database lock.

The randomization is performed by computer at a central randomization center in the AMC. Randomization will be stratified according to presence or absence of anovulation and center of treatment. Women eligible for the study will be referred to a research nurse. This person is dedicated to counsel eligible patients and will perform randomization and collect follow-up data, but will not perform the experimental intervention. We will collaborate with the research nurses who are involved in the obstetric and urogynaecological consortium. In this consortium large randomized clinical trials are performed. Information on this infrastructure is obtainable from http://www.studies-obsgyn.nl/

The study analysis will be performed on the final data that will become available after the database is formally locked.

6.4 Study analysis

The primary analysis will be by intention-to-treat. The dataset for this analysis includes all randomized subjects not violating any of the in- or exclusion criteria, who participated in study activities, and for whom post-baseline data is present.

A per-protocol analysis will also be performed, in which drop-out patients will be identified as non-compliant. Information on the primary outcome, i.e. live birth within two years after randomization will be used whenever provided. Patients who drop out of the lifestyle intervention arm will be analyzed in the “usual care” arm in this per protocol analysis.

6.5 Study participants characteristics

Baseline characteristics include female age, ethnicity, education, current smoking, parity, duration and type of subfertility (anovulation, tubal factor, male factor, unexplained, other), biometrics (BMI, waist circumference, waist/hip ratio, systolic and diastolic blood pressure), partner characteristics (age, BMI, current smoker), fertility characteristics (ovulation/anovulation, PCO yes/no, sperm analysis results according to WHO standards, tubal patency), and Hunault prognostic index.

6.6 Efficacy parameters

Intention-to-treat analysis

The outcomes with respect to pregnancy will be tabulated for both groups. Counts will be presented and percentages will be calculated in relation to the number of started participants. The following outcomes will be described:

- Vaginal delivery of a healthy singleton >37 weeks of gestation (primary outcome, twin or higher order births counted as failure).
- Live births irrespective of term or health status (multiples counted as 1).
- Ongoing and clinical pregnancies (multiples counted as 1)
- Fetal loss: ectopic pregnancy, miscarriage (<16 weeks), still birth (before or after 24 weeks)
- Type of pregnancies: singleton or multiple
- Mode of conception: spontaneous versus fertility treatment (OI, IUI, IVF/ICSI); number of fertility treatment cycles
- Complications related to fertility treatment
- Gestational complications
- Labor and delivery characteristics (including maternal complications)
- Neonatal outcome

Per-protocol analysis:

The outcomes with respect to pregnancy will be tabulated for both groups. Counts will be presented and percentages will be calculated in relation to the number of started participants. The following outcomes will be described:

- Vaginal delivery of a healthy singleton >37 weeks of gestation (primary outcome, twin or higher order births counted as failure).
- Live births irrespective of term or health status (multiples counted as 1).
- Ongoing and clinical pregnancies (multiples counted as 1)
- Fetal loss: ectopic pregnancy, miscarriage (<16 weeks), still birth (before or after 24 weeks)
- Type of pregnancies: singleton or multiple
- Mode of conception: spontaneous versus fertility treatment (OI, IUI, IVF/ICSI); number of fertility treatment cycles
- Complications related to fertility treatment
- Gestational complications
- Labor and delivery characteristics (including maternal complications)
- Neonatal outcome

Differences between groups will be expressed as relative risks and 95% confidence intervals.

The primary analysis will be by intention-to-treat.
The main analysis of the primary outcome will also consist of a Kaplan-Meier analysis of time to pregnancy leading to a vaginal delivery at term for both groups. The Log-rank test will be used to determine statistical significance. In this analysis, patients will be censured at the time they discontinue or complete the study without getting pregnant.
In addition, pregnancy rates and 95% confidence intervals per group will be calculated based on the Kaplan-Meier estimates at various time points. Further analysis of delivery rates over time will be performed using Cox-regression analysis with correction for the stratification variables ovulatory status and treatment centers as well as for confounders. These analyses will also be performed for spontaneous and treatment-induced pregnancies separately. The same analysis will be performed for the secondary outcome ongoing pregnancies. This will include ongoing pregnancies, which have not yet resulted in the birth of a child. The results of these analyses will be presented in a separate manuscript.
Exploratory subgroup analyses of the primary outcome and ongoing pregnancy rates will be performed for spontaneous and treatment-induced pregnancies and for women with a BMI below 35 versus above 35, as well as for anovulatory versus ovulatory women and for women with a waist-hip ratio of above 0.8 versus below or equal to 0.8 and for women who are 36 years or older versus younger than 36 years based on tests of interaction with treatment group. The results of these analyses will be presented in a separate manuscript.

Additional analysis concerning the influence of HOMA, androgens, adipokines and cytokines on pregnancy chances and recovery of ovulations in anovulatory patients will be assessed using multivariate Cox-regression. The results of these analyses will be presented in a separate manuscript.

6.7 Lifestyle program effectiveness

The effects of the lifestyle program will be presented as the changes in body weight, BMI, systolic and diastolic blood pressure, waist circumference and waist/hip ratio for both groups, presented as mean or median with standard deviation or interquartiele range as appropriate from randomization until the occurrence of a pregnancy. In addition, the change in percentage of ovulatory women will be compared between the groups. The results of these analyses will be presented in a separate manuscript.

7. References

1. Bayram N, van Wely M, Kaaijk EM, Bossuyt PM, van der Veen F. Using an electrocautery strategy or recombinant follicle stimulating hormone to induce ovulation in polycystic ovary syndrome: randomised controlled trial. BMJ 2004;328:192.

2. Custers IM, van Rumste MM, van der Steeg JW, et al. Long-term outcome in couples with unexplained subfertility and an intermediate prognosis initially randomized between expectant management and immediate treatment. Hum Reprod 2012;27:444-50.

3. van der Steeg JW, Steures P, Eijkemans MJ, et al. Obesity affects spontaneous pregnancy chances in subfertile, ovulatory women. Hum Reprod 2008;23:324-8.

4. Mutsaerts MA, Kuchenbecker WK, Mol BW, Land JA, Hoek A. Dropout is a problem in lifestyle intervention programs for overweight and obese infertile women: a systematic review. Human reproduction 2013;28:979-86.
