# Supplementary material for: Effect of a lifestyle intervention in obese infertile women on cardiometabolic health and quality of life: A randomized controlled trial
Source: PLoS One. 2018 Jan 11;13(1):e0190662. doi: 10.1371/journal.pone.0190662 (PMC5764284; doi:10.1371/journal.pone.0190662)
Supplement: S2 Table — Mediation analyses of change in physical activity and diet on outcomes that are improved by the intervention in comparison to the control group at three months based on primary mixed models analyses. (DOC) [file pone.0190662.s004.doc]

**Supporting information: S2 Table.**

**Table 1: Mediation analyses of change in physical activity and diet on change in weight at three months after randomisation.**

| **Mediator** | **Number** | **Indirect/Total effect** | **95% CI indirect effect** |
| --- | --- | --- | --- |
| **Δ Total MVPA ^α^** | 235 | 0.0639/-3.2362 = -0.0197 | -0.0487 to 0.3075 |
| **Δ Total LT ^β^** | 235 | 0.0461/-3.2362 = -0.0143 | -0.0131 to 0.2592 |
| **Δ Fruit intake** | 240 | -0.0142/-2.9283 = 0.0049 | -0.1647 to 0.0299 |
| **Δ Vegetable intake** | 238 | -0.0123/-2.9798 = 0.0041 | -0.1839 to 0.0302 |
| **Δ Sugary drink intake** | 192 | -0.0703/-3.1538 = 0.0223 | -0.3041 to 0.0159 |
| **Δ Sweet snack intake** | 220 | 0.0093/-3.1384 = -0.0030 | -0.0254 to 0.1439 |
| **Δ Savory snack intake** | 222 | -0.1694/-3.0265 = 0.0560 | -0.5577 to 0.0044 |

^α^ MVPA = Moderate to vigorous physical activity. ^β^ LT = Leisure time

**Table 2: Mediation analyses of change in physical activity and diet on change in BMI at three months after randomisation**.

| **Mediator** | **Number** | **Indirect/Total effect** | **95% CI indirect effect** |
| --- | --- | --- | --- |
| **Δ Total MVPA** | 226 | 0.0166/-1.1445 = -0.0145 | -0.0227 to 0.1002 |
| **Δ Total LT** | 226 | 0.0117/-1.1445 = -0.0102 | -0.0062 to 0.0886 |
| **Δ Fruit intake** | 230 | -0.0020/-1.0113 = 0.0020 | -0.0415 to 0.0161 |
| **Δ Vegetable intake** | 228 | -0.0042/-1.0268 = 0.0040 | -0.0634 to 0.0144 |
| **Δ Sugary drink intake** | 182 | -0.0260/-1.1194 = 0.0233 | -0.1082 to 0.0097 |
| **Δ Sweet snack intake** | 210 | 0.0015/-1.1024 = -0.0014 | -0.0087 to 0.0486 |
| **Δ Savory snack intake** | 212 | -0.0673/-1.0619 = 0.0634 | -0.2107 to 0.0004 |

^α^ MVPA = Moderate to vigorous physical activity. ^β^ LT = Leisure time

**Table 3: Mediation analyses of change in physical activity and diet on change in waist circumference at three months after randomisation.**

| **Mediator** | **Number** | **Indirect/Total effect** | **95% CI indirect effect** |
| --- | --- | --- | --- |
| **Δ Total MVPA** | 227 | -0.1287/-2.2861 = 0.0563 | -0.5189 to 0.0726 |
| **Δ Total LT** | 227 | -0.0811/-2.2861 = 0.0355 | -0.3854 to 0.0546 |
| **Δ Fruit intake** | 232 | 0.0090/-1.9401 = -0.0046 | -0.1069 to 0.2451 |
| **Δ Vegetable intake** | 230 | -0.0290/-2.0263 = 0.0143 | -0.3250 to 0.0482 |
| **Δ Sugary drink intake** | 187 | -0.1233/-2.3562 = 0.0523 | -0.6503 to 0.1980 |
| **Δ Sweet snack intake** | 214 | 0.0655/-2.2973 = -0.0285 | -0.0546 to 0.3617 |
| **Δ Savory snack intake** | 216 | -0.0861/-2.2283 = 0.0386 | -0.4092 to 0.1649 |

^α^ MVPA = Moderate to vigorous physical activity. ^β^ LT = Leisure time

**Table 4: Mediation analyses of change in physical activity and diet on change in hip circumference at three months after randomisation**.

| **Mediator** | **Number** | **Indirect/Total effect** | **95% CI indirect effect** |
| --- | --- | --- | --- |
| **Δ Total MVPA** | 224 | -0.0805/-3.0674 = 0.0262 | -0.4857 to 0.0623 |
| **Δ Total LT** | 224 | -0.0491/-3.0674 = 0.0160 | -0.4125 to 0.0351 |
| **Δ Fruit intake** | 229 | 0.0069/-2.6907 = -0.0026 | -0.0938 to 0.1965 |
| **Δ Vegetable intake** | 227 | 0.0066/-2.7877 = -0.0024 | -0.0790 to 0.1914 |
| **Δ Sugary drink intake** | 184 | -0.1943/-2.6335 = 0.0738 | -0.6033 to 0.0237 |
| **Δ Sweet snack intake** | 211 | 0.0230/-2.8061 = -0.0082 | -0.0710 to 0.2533 |
| **Δ Savory snack intake** | 213 | 0.0017/-2.6002 = -0.0007 | -0.2407 to 0.2705 |

^α^ MVPA = Moderate to vigorous physical activity. ^β^ LT = Leisure time

**Table 5: Mediation analyses of change in physical activity and diet on change in insulin concentration at three months after randomisation**.

| **Mediator** | **Number** | **Indirect/Total effect** | **95% CI indirect effect** |
| --- | --- | --- | --- |
| **Δ Total MVPA** | 206 | 0.0366/-2.1086 = -0.0174 | -0.1507 to 0.3103 |
| **Δ Total LT** | 206 | 0.0057/-2.1086 = -0.0027 | -0.0940 to 0.2171 |
| **Δ Fruit intake** | 210 | -0.0683/-2.1621 = 0.0316 | -0.4366 to 0.0414 |
| **Δ Vegetable intake** | 208 | 0.0061/-2.1432 = -0.0028 | -0.1900 to 0.2728 |
| **Δ Sugary drink intake** | 168 | -0.3998/-1.6762 = 0.2385 * | -0.9163 to -0.0754 |
| **Δ Sweet snack intake** | 193 | -0.0156/-2.3272 = 0.0067 | -0.2273 to 0.0650 |
| **Δ Savory snack intake** | 198 | -0.2618/-2.1663 = 0.1208 * | -0.6885 to -0.0302 |

^α^ MVPA = Moderate to vigorous physical activity. ^β^ LT = Leisure time * statistically significant indirect effect of mediator on outcome (p=<0.05).

**Table 6: Mediation analyse**s of change in physical activity and diet on change in HOMA-IR at

| **Mediator** | **Number** | **Indirect/Total effect** | **95% CI indirect effect** |
| --- | --- | --- | --- |
| **Δ Total MVPA** | 199 | 0.0166/-0.5706 = -0.0290 | -0.0207 to 0.1173 |
| **Δ Total LT** | 199 | 0.0044/-0.5706 = -0.0077 | -0.0143 to 0.0851 |
| **Δ Fruit intake** | 201 | -0.0261/-0.5960 = 0.0438 | -0.1331 to 0.0103 |
| **Δ Vegetable intake** | 200 | 0.0082/-0.5718 = -0.0144 | -0.0360 to 0.1021 |
| **Δ Sugary drink intake** | 160 | -0.1069/-0.4631 = 0.2308 * | -0.2528 to -0.0175 |
| **Δ Sweet snack intake** | 184 | -0.0116/-0.5904 = 0.0197 | -0.0772 to 0.0084 |
| **Δ Savory snack intake** | 189 | -0.0654/-0.5392 = 0.1214 * | -0.1860 to -0.0087 |

**three months after randomisation.**

^α^ MVPA = Moderate to vigorous physical activity. ^β^ LT = Leisure time * statistically significant indirect effect of mediator on outcome (p=<0.05).

**Table 7: Mediation analyses of change in physical activity and diet on metabolic syndrome status at three months after randomisation.**

| **Mediator** | **Number** | **Indirect/Total effect** | **95% CI indirect effect** |
| --- | --- | --- | --- |
| **Δ Total MVPA** | 182 | -0.0170/1 = -0.0170 | -0.1892 to 0.0799 |
| **Δ Total LT** | 182 | -0.0091/1 = -0.0091 | -0.1486 to 0.0400 |
| **Δ Fruit intake** | 184 | -0.0026/1 = -0.0026 | -0.0901 to 0.0429 |
| **Δ Vegetable intake** | 182 | 0.0014/1 = 0.0014 | -0.0616 to 0.0787 |
| **Δ Sugary drink intake** | 147 | 0.0214/1 = 0.0214 | -0.1260 to 0.2106 |
| **Δ Sweet snack intake** | 169 | -0.0118/1 = -0.0118 | -0.1975 to 0.0132 |
| **Δ Savory snack intake** | 172 | -0.0651/1 = -0.0651 | -0.2865 to 0.0338 |

^α^ MVPA = Moderate to vigorous physical activity. ^β^ LT = Leisure time
